# Physical activity simultaneously improves working memory and ripple-spindle coupling

**DOI:** 10.1038/s42003-025-08618-3

**Published:** 2025-08-08

**Authors:** Xinyun Che, Benedikt Auer, Paul Schmid, Christoph Reichert, Annemarie Scholz, Tom Weischner, Robert T. Knight, Stefan Dürschmid

**Affiliations:** 1 Leibniz Institute for Neurobiology, Magdeburg, Germany; 2 Departments of Psychology and Neuroscience, University of California Berkeley, Berkeley, CA USA; 3 Helen Wills Neuroscience Institute, University of California Berkeley, Berkeley, CA USA

**Keywords:** Cognitive neuroscience, Human behaviour

## Abstract

Ripples, representing the compressed reactivation of environmental information, provide a mechanism for retaining memory information in chronological order and are also crucial for working memory (WM) during wakefulness. Brief sessions of physical activity (PA) are proposed to boost WM. In concurrent EEG/MEG sessions, we investigated the role of PA in WM performance and high-frequency-ripple to wake spindle coupling. Ripples, identified in MEG sensors covering the medial temporal lobe (MTL) region, predicted individual WM performance. Ripples were locked to robust oscillatory patterns in the EEG defined spindle band. Wake spindle activity and ripples decrease during initial stimulus presentation and rebound after 1 sec. Behaviorally, PA enhanced WM performance. Neurophysiologically, PA scaled the ripple rate with the number of items to be kept in WM and strengthened the coupling between ripple events and wake spindle events. These findings reveal that PA modulates WM by coordinating ripple-spindle interaction.

## Abstract (web summary)

After short-term Physical activity (PA), working memory (WM) performance improved. Ripple activity increased with memory load, and ripple to awake-spindle coupling strengthened, suggesting PA boosts WM via hippocampal-thalamocortical dynamics.

## Introduction

Working memory (WM) allows information to be stored in a sequential order for future retrieval. A common method to test working memory capacity in humans is the N-back task requiring subjects to match items that appeared in previous trials. In the N-back task, target detection performance decreases with an increasing amount of information to be retained due to the finite capacity of WM. Brief sessions of physical activity (PA) like walking^1,2^ or cycling^3^ enhance working memory performance^3–7^ likely due to increased hippocampus-cortex coupling^3^. However, the neurophysiological mechanism for retaining information in chronological order within WM and the impact of PA on this mechanism remain unclear. Sleep research suggests a specific interaction between high-frequency ripples and thalamic generated spindle activity as a key mechanism for successful integration of information into memory^8–10^. High-frequency ripple events, manifesting as transient bursts (~80–150 Hz in humans^11^), are considered a compressed reactivation of sequential environmental information during cued recall. Spindle activity is assumed to establish a time frame for ripples occurrence^11^. However, whether ripple-spindle coupling during wakefulness is a mechanism for memory formation is unknown.

In simultaneous EEG/MEG recordings, we assessed whether performing an N-back WM task is tracked by modulation in wake spindle activity, ripple rate and spindle-ripple coupling. Specifically, we tested whether PA improved both WM capacity and MEG-ripple – EEG-spindle coupling. In rest session, participants remained inactive in short experimental breaks (2 min) of typical EEG/MEG recordings. In PA session, participants were engaged in movement during breaks (2 min) using an MEG-compatible pedal trainer designed to facilitate independent forward and backward movements, resembling a walking motion.

Physical activity (PA) enhanced working memory performance. High-frequency ripples, key for organizing information into working memory, were detected in MEG sensors covering the medial temporal lobe (MTL) region. Both EEG-spindle and MEG-ripple rates decreased during stimulus presentation but increased after 1 sec. EEG-wake spindle activity was higher in the PA session compared to rest during rebound. The rebound MEG-ripple rate predicted individual WM performance and scaled with the number of items retained in WM. Critically, PA increased coupling of MEG-ripples to EEG-spindle activity and also modulated WM performance.

## Results

### Procedure

**Fig. 1:**
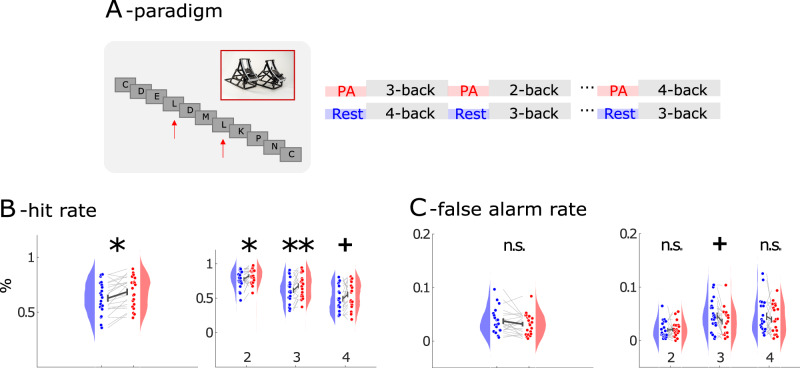
Behavioral performance. **A** Task design. Subjects were instructed to rest or use a pedal trainer before the N-back task. **B** (left): shows that hit rate increased across all conditions. (right): Hit rate across different N-back conditions. Error bars indicate the standard error of the mean. (*n* = 21; +: *P* < 0.05, *: *P* < 0.017, **: *P* < 0.003, Bonferroni-corrected). **C** (left): Individual false alarm rate. (right): False alarms across different N-back conditions. Red indicates PA and blue rest session. (*n* = 21; +: *P* < 0.05, n.s.: not statistically significant).

21 participants participated in the experiment with simultaneous EEG and MEG recording. The experiment was conducted in two sessions, with and without PA, on two different days. The order of PA and rest was counterbalanced across subjects. Each session consisted of 12 blocks. During breaks between blocks subjects were instructed to rest or use an MEG compatible pedal trainer for two minutes. The pedal trainer permitted independent forward and backward movements (see Fig. 1A and Methods). Participants were directed to pedal at a moderate speed, adjusting their pace individually, akin to a walking motion. Within each block, an item (one of the 26 letters of the Latin alphabet) was a target if it matched the item from *N* = 2, *N* = 3, or *N* = 4 trials ago (N-back task), requiring subjects to hold a varying number of items in working memory in each block (see Fig. 1 A and Methods for a detailed description of the procedure).

### Behavioral performance

First, we evaluated target detection accuracy (hit rate; H; see Fig. 1B) and false alarms (see Fig. 1C). When we compared hit rate, we found a main effect of PA (F_(1,120)_ = 4.04; *p* = 0.0042) and N-back condition (F_(2,120)_ = 31.7; *p* < 0.00001) compared against a surrogate distribution (see Methods). Performance declined with number of items to be held in working memory (H_2_ = 80.07%; H_3_ = 64.68%; H_4_ = 52.12%). However, hit rate was higher after PA than after rest (H_PA_ = 68.51%; H_rest_ = 62.74%; t_20_ = 3.62; *p* = 0.0017) in 2 and 3 back conditions at the Bonferroni-corrected level (2-back: t_20_ = 2.78; *p* = 0.012; 3-back: t_20_ = 3.39; *p* = 0.003; 4-back: t_20_ = 2.21; *p* = 0.039, see Fig. 1B). When we compared false alarm rates, no significant effect of PA was found (F_(1,120)_ = 1.56; *p* = 0.214, see Fig. 1C).

### EEG spindle activity

**Fig. 2:**
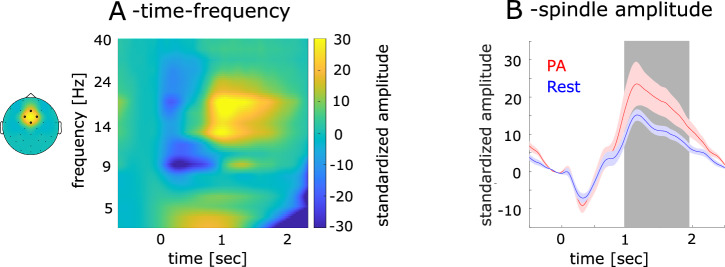
Frequency amplitude of EEG low frequency, standardized spindle and theta amplitude and ripples. **A** EEG amplitudes modulation in frequencies 1–40 Hz as a function of time. **B** The amplitude modulation of the spindle band (14–18 Hz) across time for physical activity (red line) and rest (blue line). Shaded areas represent the standard error of the mean across subjects. The gray shading indicates the interval displaying significant differences between rest and PA. Time 0 represents stimulus onset.

Spindle activity provides a temporal framework for ripple occurrence^11^. In the EEG, thalamic spindle activity is maximal at fronto-central electrodes^12^. We found a consistent temporal pattern across both sessions, with spindle activity decreasing immediately after stimulus onset, followed by a subsequent increase at central EEG electrodes (see Fig. 2A, left), peaking at 1150 msec. Additionally, enhanced activity between 1 and 2 sec after stimulus presentation was observed in the PA conditions (t_20_ = 2.20; *p* = 0.04, see Fig. 2B).

### Ripple activity

**Fig. 3:**
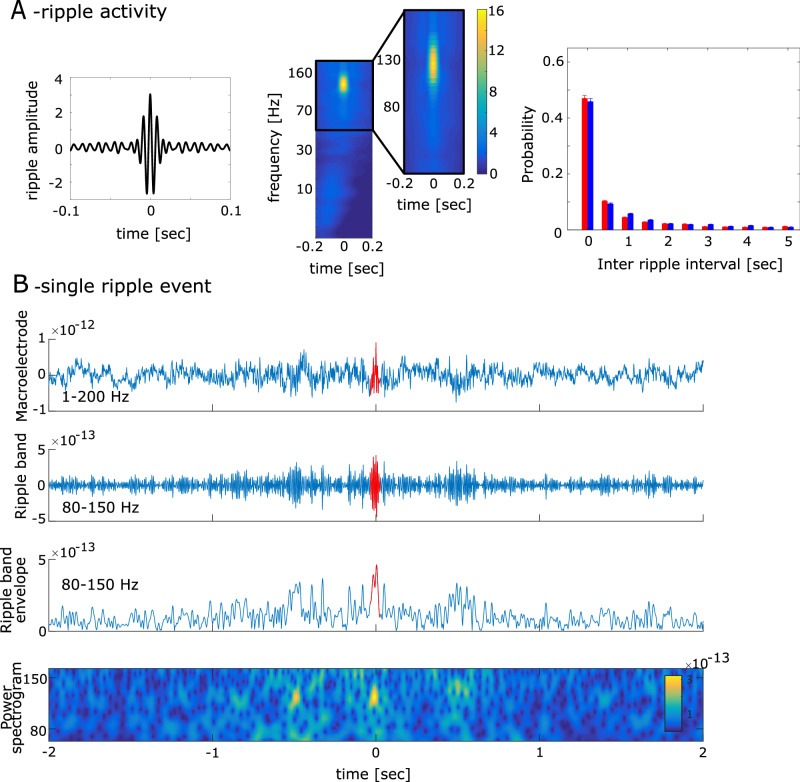
high frequency ripple activation in MEG. **A** (left): The grand-averaged signal of ripple activity. (middle): Time-frequency plot of ripple activity. (left): The probability of the interval time between two ripples across subjects. The error bars represent the standard deviation. The blue bar represents the ripple interval during the rest session, and the red bar represents the ripple interval during the PA session. **B** An example of ripples detected in our analysis. The upper line shows the broad band signal between 1 and 200 Hz. The second time series shows the same signal in the ripple band. The third time series shows the Hilbert transform of the ripple band activity. The last graphic shows the time frequency representation of the signal filtered between 80 and 150 Hz.

Ripples were first observed in rodent hippocampus^13^ and later in humans in a frequency range between 80 and 150 Hz^14–16^. We detected ripple events in the ongoing MEG signal in accord with previous studies^8,17,18^ (see Fig. 3A and Methods). Ripples in MEG resembled the time-frequency representation (mean duration = 31.91 msec, SD = 10.82 msec; ripple density = 0.125 Hz, SD = 0.098 Hz, see Fig. 3B) observed in intracranial studies^8,17,18^.

### Topographical likelihood distribution of ripples

**Fig. 4:**
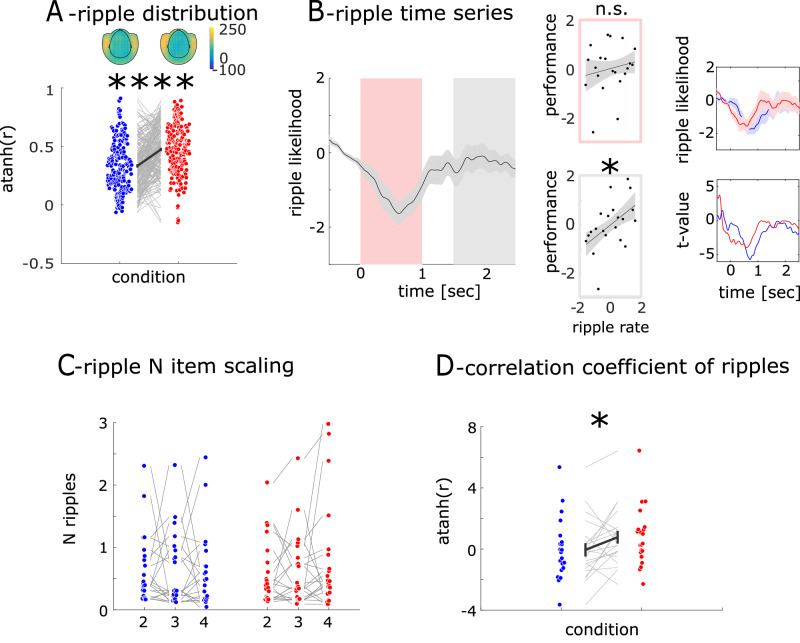
Ripple modulation. **A** (upper): Topographical map of ripple likelihood across MEG sensors for rest (left) and PA (right). (lower): Correlation coefficients of ripple distributions between pairs of subjects. Higher correlations coefficients following PA indicate a more consistent spatial structure of ripples likelihood across subjects. Error bars indicate the standard error of the mean. (*n* = 210; ****P < 0.0001). **B** (left): Time series of ripple likelihood. Shaded areas represent the standard error of the mean across subjects. (middle): linear regression between individual memory capacity and ripple likelihood in segmented intervals. (right): The ripple likelihood time series from grand-averaged and different sessions, along with the corresponding t-values. (*n* = 21; *P < 0.05, n.s.: not statistically significant). **C** Ripple count across N-back conditions displayed individually. **D** Correlation coefficients for rest (blue) and PA (red) calculated between the ripple likelihood and the 2, 3, 4-back levels. (*n* = 21; *P < 0.05).

We defined the topographical distribution of ripple events across the entire experiment. For each sensor, we determined the ripple likelihood as the average number of ripples across all trials, including targets and standards, leading to a likelihood value for each of the 102 MEG sensors and each participant. We then correlated the spatial likelihood distribution between all possible pairs of participants. This resulted in 210 correlation values both for rest and PA. We tested whether the distribution of ripples differed between PA and rest (see Fig. 4A). Following PA, pairs of participants exhibited stronger topographical consistency in ripple distribution (mean r_rest_ = 0.37; mean r_PA_ = 0.56; t_209_ = 8.07; *p* < 0.0001) with sensors covering the MTL region showing the highest ripple likelihood.

### Ripple time series

In the next step, we defined the time course of ripple events around stimulus presentation. We found that the averaged ripple likelihood decreased after stimulus onset (from 280 msec to 970 msec) with a rebound starting around 1.5 sec and peaking around 2 sec (see Fig. 4B).

### The correlation of ripples with memory capacity

We then tested whether the ripple frequency predicted individual hit rate. We found no correlation in the first sec after stimulus presentation (*r* = 0.17; *p* = 0.46). However, ripple rate between 1.5 and 2.5 sec predicted individual target detection rate (*r* = 0.49; *p* = 0.021, see Fig. 4B). In the first step we compared ripple rate in this interval between conditions but did not find a difference (t_20_ = 0.43; *p* = 0.67). When we compared the ripple density between conditions we found a significant difference between N-back conditions following PA (*F* = 3.24, *p* = 0.046) but not following rest (*F* = 0.84, *p* = 0.437). Furthermore, we found that the ripple density across participants was strongly correlated between blocks following PA (p_corrected_ = 0.05/6 = 0.0083; 2 vs 3-back: r = 0.7156; 2 vs 4-back: r = 0.6740; 3 vs 4-back: r = 0.620; all *p*-values < 0.0034) but not following rest (2 vs 3-back: r = 0.546; 2 vs 4-back: r = 0.2922; 3 vs 4-back: r = 0.5468; all *p*-values > 0.01). This result indicates that inter-individual differences remain consistent across N-back conditions following PA but not rest. Next, we tested whether the number of ripples is scaled with the number of items to be held in working memory (see Fig. 4C). We conducted a correlation analysis between N-back and ripple rate for each participant leading to a correlation value for each subject. Using directed t-tests under the assumption that r-values are higher than zero (more ripples when more items are held in WM) we found a positive linear scaling of N-back and ripples in the PA session (r_PA_ = 0.77; t_20_ = 1.86; *p* = 0.0385). No such effect was observed in the rest session (r_Rest_ = -0.05; t_20_ = 0.12; *p* = 0.452). The N-back ripple correlation in the PA session was stronger than in the rest session (t_20_ = 2.12; *p* = 0.024, see Fig. 4D).

### MEG-Ripple to EEG-low-Frequency coupling

#### Ripple centered EEG epochs

**Fig. 5:**
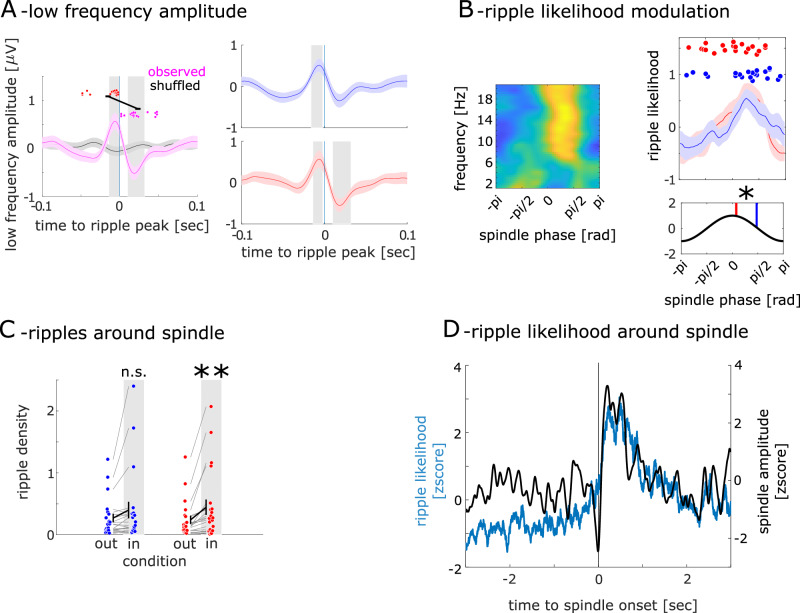
MEG-Ripple and EEG low-frequency coupling results. **A** (left) Grand-averaged slow oscillations around the ripple peak compared to the shuffled peak. The dots at the top represent slow oscillation amplitude peaks and troughs from individual subjects. The red dots indicate amplitude peaks, while the pink dots indicate troughs. Error bars indicate the standard error of the mean. (right): The slow oscillations around the ripple peak from different sessions. The blue color represents the rest session, and the red color represents the PA session. **B** (left): Phase distribution of low frequency around the ripple peak. (right upper): The phase distribution from 14 to 16 Hz from different sessions and the dominant phase from individual subjects. **B** (right lower): Average dominant phases across sessions. (*n* = 21; *P < 0.05). **C** The ripple density during the ‘in-spindle’ and ‘out-of-spindle’ phases is plotted separately for the PA and rest sessions. Red denotes PA session data, while blue represents rest session data. Error bars indicate the standard error of the mean. (*n* = 21; **P < 0.01, n.s.: not statistically significant). **D** Ripple likelihood around the spindle onset. The time 0 represents the spindle onset.

We tested whether we could find coupling between MEG-ripples and EEG-spindles. Note that we did not find MTL spindle activity in the MEG (see Fig. 3A). We epoched EEG data around MEG-ripple events and found a clear oscillation locked to ripples (see Fig. 5A). This oscillation showed a significant positive amplitude modulation from −14 to 0 msec (t_max_ = 3.24 at -6 msec relative to ripple peak; *p* = 0.0041; interval I) and a significant negative amplitude modulation from 10 to 32 msec (t_max_ = 4.08 at 24 msec following ripple peak; *p* = 0.0006; interval II, see Fig. 5A). No such difference could be found for randomized ripple events (interval I: t_max_ = 1.26; *p* = 0.57; interval II: t_max_ = 0.99; *p* = 0.32, see Fig. 5A). This pattern can be explained by oscillatory modulation around ripples in the PA session with a stronger modulation in interval II (interval I: t_max_ = 2.9 at −6 msec; p = 0.011; interval II: t_max_ = 3.7 at 22 msec; *p* = 0.0014). No such effect was found in the rest session (interval I: t_max_ = 3.32; *p* = 0.003; interval II: t_max_ = 2.2; *p* = 0.032)

#### Ripple-peak to spindle-phase coupling

We then investigated whether ripples are coupled to a specific low-frequency phase. We analyzed the phase of EEG low-frequency activity associated with the ripple peak (see Fig. 5B) and tested whether ripples are differently locked to the spindle band (see Fig. 5B). We found that ripples were locked to different phases φ depending on the session (φ_PA_ = 0.24 rad; φ_rest_ = 1.49 rad; F = 7.05; *p* = 0.011).

### Temporal coordination of ripples with wake spindle events

**Fig. 6:**
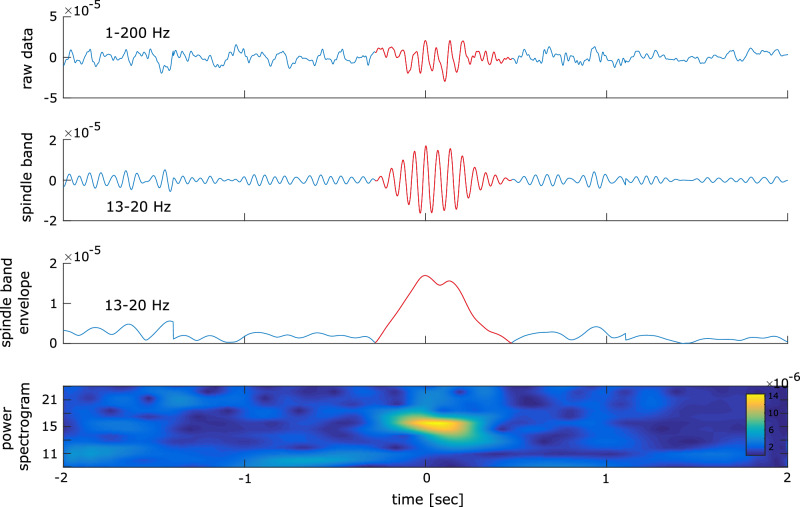
spindle activation in EEG. An example of spindle detected in our analysis. The upper line shows the broad band signal between 1 and 200 Hz. The second time series shows the same signal in the spindle band. The third time series shows the Hilbert transform of the spindle band activity. The last graphic shows the time frequency representation of the signal filtered between 13 and 20 Hz.

We examined whether ripples were temporally locked to wake spindle events by comparing the frequency of ripples outside and within wake spindle events (see Fig. 6, and Methods for a definition of wake spindle events). In the PA session, we observed more ripples within than outside wake spindle events (N_in_ = 0.45; N_out_ = 0.24; t_20_ = 3.25; *p* < 0.01, see Fig. 5C), but only a trend in the rest session (N_in_ = 0.40; N_out_ = 0.27; t_20_ = 1.93; *p* = 0.068). In the final step, we investigated when the ripple likelihood reaches its maximum relative to the wake spindle peak event. We found a peak in the ripple likelihood starting from 102 msec after the spindle onset (*p* < 0.0001, see Fig. 5D).

## Discussion

We investigated the impact of PA on working memory observing WM performance increases after PA in all N-back conditions. EEG-wake spindle activity was heightened in the PA session compared to rest. High-frequency ripples linked to the temporal organization of WM information were detected in MEG sensors covering the MTL region and the number of ripples predicted individual WM performance. The ripple rate was comparable between PA and rest. However, it increased with the number of items held in WM only after PA. Additionally, ripple-spindle coupling also increased following PA. Ripple activity has been reported to increase in the medio-temporal areas (hippocampus, amygdala, and parahippocampal gyrus) following 20 min of mini-bike exercise^19^. Moreover, a 10 min mild cycling exercise was found to enhance memory performance by increasing coupling between the hippocampus and cortical regions^3^. While evidence for the role of wake spindles in memory processing is limited, it has been reported that the neural sink and source of wake spindles are similar to those of sleep spindles^20^. Additionally, visual inspection of recordings from wired electrodes in rats indicated that wake spindles are associated with ripple activity, much like sleep spindles^20^.

Moderate PA improved working memory capacity in line with previous studies. Abou Khalil et al.^2^ found a performance increase in a word-recall task during brisk walking compared to sitting. Previous studies found that concurrent moderate exercise lead to an improvement in WM accuracy and faster reaction times^1^ and short bouts of exercise improved memory consolidation^21^. In summary, our findings contribute to the growing body of evidence indicating that short-term physical activity modulates memory processes.

Spindle activity during non-rapid-eye-movement (NREM) sleep increases during memory consolidation^8,9^. It has been proposed that spindles play a key role in regulating information flow between the cortex and hippocampus areas, supporting memory processing through the reactivation of prior experiences and enhancing structural changes at cortical sites during learning. The increase in ripples is predictive of individual performance differences^18,22,23^. The impact of PA on sleep spindle activity is uncertain, with some studies suggesting that daily repeated exercise can enhance 13–16 Hz spindle power during sleep^24^, while others did not find effects of activity on spindle activity^25^. We observed spindle activity in both PA and rest sessions, topographically overlapping with sleep spindle findings in previous studies^12^. Notably, the amplitude of sleep spindles increased after the working memory task. These findings suggest that PA improves working memory performance by enhancing spindle activity.

Ripple events, considered reactivation patterns, manifest as transient high frequency bursts (~80–150 Hz in humans: mean duration ~30 msec). Ripple events were first studied in the hippocampus and more recently identified in extrahippocampal regions^11,26^, particularly during sleep. Numerous studies highlight the crucial role of hippocampal sleep ripples in the initial formation of declarative memory traces, suggesting their involvement in anatomically distributed memory traces^27^. Hippocampo–thalamocortical synchronization during sleep is key to human memory consolidation^28^. A prominent model posits that embedding novel information in the neocortex requires offline reactivation by hippocampal generated ripple events, primarily during slow-wave sleep^22,29^. Recent studies have also reported ripples during waking, influencing emotional memory encoding and discrimination^30^. Ripples in the hippocampus, MTL, and neocortex are reported to mirror neuron firing patterns during cued recall. Awake ripples share similarities in density, frequency, and duration during both waking and sleep^15^. Episodic memory studies in human subjects support the role of the hippocampus and surrounding cortical areas in learning and recalling temporal sequences. We show that MTL ripple rate scales with the number of items held in working memory and predicts individual working memory capacity. Ripples show a specific time course with the ripple rate decreasing during stimulus presentation, rebound around 1 sec, and predict individual performance.

We observed that ripple rate predicted the number of items in working memory post-PA aligning with prior research indicating that ripple rate scales with the complexity of autobiographical memory^31^. We also identified a correlation between ripple activity and hit performance, consistent with previous results reporting a correlation between ripple rate and successful memory retrieval^22^.

Spindles and high-frequency ripple activity were co-modulated and both were modulated by PA. Spindle-ripple coupling was initially described during rat slow-wave sleep^32^ and reported in epilepsy patients during sleep using intracranial EEG (iEEG) or parahippocampal foramen ovale (FO) electrodes^11,14,33^. In line with these studies, we found coupling of EEG spindle activity with MEG ripple events in both the PA and rest sessions. However, PA modulated coupling through phase- and temporal alignment with spindle activity, exhibiting a stronger spindle modulation around ripple events. This coupling property is consistent with previous studies showing that ripple rates increase during the ‘waxing’ spindles phase (the raising phase) from the iEEG recording at human MTL regions^11^. Physical activity shifted the phase of ripple-coupled spindles, temporally aligning ripples with high-amplitude spindle events. Our results indicate that short-term PA modulates spindle-ripple coupling, contributing to improved memory formation. It is proposed that coupling creates a temporal window for communication between the hippocampus and neocortical regions^34,35^. The spindle-ripple coupling and the phase shift observed during PA session may signify modulated communication among reactivation processes between cortical and subcortical structures enhancing working memory.

## Methods

### Participants

Twenty one participants (13 female, 8 male, mean age = 27.00 years, SD = 5.76 years) participate in the experiment. Nine participants of an initial pool of thirty subjects dropped out after the first session due to unwillingness to return, excessive movement during the memory task, or difficulty understanding the task. A 60/40% gender split was maintained, which is sufficient to provide reliable and generalizable results, as the effects of gender on working memory performance are typically small. All participants were informed of the study and provided written consent to the experimental protocol prior to participation. The recordings took place at the Department of Neurology, Otto-von-Guericke University Magdeburg and were approved by the local ethics committee (“Ethical Committee of the Otto-von-Guericke University Magdeburg”). All ethical regulations relevant to human research participants were followed. Participants reported normal or corrected to normal vision and no history of neurological or psychiatric diseases. We invited participants to two separate MEG sessions with an average 75-day (SD = 65.27) interval between sessions to allow for a within-subject design. They either started with a PA or rest session counterbalanced across participants.

### Procedure

The stimulus presentation and experimental control were carried out using Matlab R2009a (Mathworks, Natick, USA) and the Psychophysics Toolbox^36^. The screen color was set to gray, while the stimuli and instructions were displayed in black. The experiment consisted of 12 blocks, each comprising 100 trials of an N-back task. Target items were defined as items that matched the item presented in either *N* = 2, *N* = 3, or *N* = 4 trials before the current trial requiring subjects to hold a varying number of items in their working memory. Each block consisted of 30 targets and 70 standards. Stimuli included 26 letters of the Latin alphabet, with targets and standards randomly selected. The experiment started with a practice session involving *N* = 2 and *N* = 4 back tasks, which only included 6 targets and 14 standards, under identical presentation settings as the main experiment. Each block began with a 2-minute phase before the actual visual stimuli were presented. Participants either used the pedal trainer or remained seated, depending on the session (rest vs. PA). Each block lasted 252 sec. Each trial started with a fixation cross presented for 500 msec, centered on the screen. The participants were instructed to focus the fixation cross during the entire experiment to minimize eye movements. Subsequently, the fixation cross was replaced by a letter stimulus for 500 msec, followed by the reappearance of the fixation cross for 2000 ± 100 msec.

Participants were instructed to respond as quickly and accurately as possible. They were instructed to use their index finger to respond to standards and their middle finger to respond to targets. The responses were provided with the left and right hands, which were alternated every three blocks. The pedal trainer was specifically designed in-house for MEG recording. All materials were carefully tested to ensure they contained no magnetic components. The device’s angle and height can be adjusted to suit each participant’s proportions, and its design allows for independent forward and backward movement of both feet. Participants were instructed to pedal at a moderate, self-adjusted pace, similar to a walking motion. While this activity resembles aerobic exercise—defined as movement sustained by oxygen delivered through the blood to active muscles—it did not have the prolonged duration typical of aerobic workouts^37^. This adjustment was made to minimize MEG artifacts, such as ECG interference or changes in skin impedance caused by sweating^38^, which can affect EEG electrode contact. Similar moderate-intensity exercises, such as wheel running in rodents, have been shown to enhance hippocampal plasticity and improve memory, linking our findings and previous animal research^5^.

### MEG recording

Participants were equipped with metal-free clothing and seated in a dimmed, magnetically shielded recording booth. Stimuli were presented via rear projection onto a semi-transparent screen with an LCD projector (DLA-G150CLE, JVC, Yokohama, Japan) that was positioned outside the booth. The screen was placed at a viewing distance of 100 cm in front of the participants. The display screen resolution was set to 1920 × 1080. Each letter presented on the screen measured approximately 3 × 3.5 cm. The screen refresh rate was 120 Hz, corresponding to an 8.3 msec refresh interval. Responses were given with the left and right hands via an MEG compatible LUMItouch response system (Photon Control Inc., Burnaby, DC, Canada). Acquisition of MEG data was performed in a sitting position using a whole-head Elekta Neuromag TRIUX MEG system (Elekta Oy, Helsinki, Finland), containing 102 magnetometers and 204 planar gradiometers. The sampling rate was set to 2000 Hz. EEG was recorded with 30 passive electrodes. The right mastoid was used as a reference electrode. Vertical EOG was recorded using one surface electrode above and one below the right eye. For horizontal EOG, one electrode on the left and right outer canthus was used. Preparation and recordings took about 3 hours.

### Preprocessing and artifact rejection

Maxwell filtering was applied to reduce external noise and both MEG and EEG data were down sampled to 500 Hz. We used Matlab 2016b (Mathworks, Natick, USA) for analysis. We included the 102 magnetometers in our analyses but no gradiometers. All filtering (see below) was done using zero phaseshift IIR filters (fourth order butterworth filter; filtfilt.m in Matlab). First, we filtered the data between 1 and 200 Hz. Then, we notch-filtered the data to discard line noise (50 Hz and its 2nd and 3rd harmonic). To discard trials of excessive, non-physiological amplitude, we used an individual threshold for each subject. Both for MEG and EEG we calculated the mean variance across time and channels for each trial. Trials with a variance exceeding 4 standard deviations were excluded. We then visually inspected all data and excluded epochs exhibiting excessive muscle activity, as well as time intervals containing artifactual signal distortions, such as signal steps or pulses.

EEG data were re-referenced to the left mastoid and band-pass filtered between 1 to 40 Hz. To eliminate eye movement artifacts, we applied Independent Component Analysis (ICA), which was computed with the FastICA package for MATLAB (Version 2.5). Resulting MEG and EEG time series were used to characterize ripple band dynamics (see below) and low frequency activity, respectively, over the time course of visual target detection. Both MEG and EEG data were epoched into trials ranging from −2 to 3 sec relative to stimulus onset (sufficiently long to prevent edge effects).

### Statistical analysis

In this study, we aimed to explore the effects of physical activity on working memory and the associated changes in brain states, specifically focusing on spindle and ripple activity as well as their coupling effect. First, we compared target detection performance and reaction times between PA and rest across various N-back conditions (Behavioral Results). In the next step, we examined EEG spindle activity (Spindle activity). Subsequently, we employed MEG signals to compute ripple activity and investigated correlations between task performance, memory load with ripple activity (Ripple activity). Lastly, we tested for spindle-ripple coupling and differences between rest and PA (Spindle-Ripple Coupling).

### Behavioral performance

We evaluated target detection accuracy based on hit rate (DA; percentage of target response when a target was present), false alarms (FA; percentage of target response when no target was presented), and reaction times (RT). We compared the impact of PA and rest on behavioral measures using a two-way ANOVA with the factors motion (PA vs. rest) and N-back (2-back, 3-back, 4-back) separately for DA, FA and RT. The performance of PA and rest sessions was compared using paired-sample t-tests at both the grand average level and for individual N-back levels. To account for multiple comparisons, we applied a Bonferroni-corrected significance level (*p* = 0.05/3) to define statistical significance. To address potential violations of sphericity and normality in the data, we compared the observed F-values to a surrogate distribution. This distribution was generated by randomly shuffling individual behavioral data across participants and conditions (motion and N-back) in 1000 iterations, ensuring that the resulting distribution preserved the same properties of sphericity and normality. For each iteration, we recalculated the F-values, resulting in a distribution of 1000 F-values. The probability (*p*-value) of the observed F-value was then determined based on its position within this surrogate distribution.

### EEG spindle activity

Our study aimed to explore whether specific frequency bands, particularly spindle activity, were modulated by the stimulus. By analyzing spindle activity after stimulus onset, we sought to identify task-related changes in brain dynamics. Spindle activity is believed to create a temporal window for ripple events to occur^11^. In the EEG, spindle oscillations generated within thalamocortical loops are primarily measured at fronto-central electrodes^12^. Both spindle and beta activity fall within the same frequency range, with beta activity also linked to working memory performance. To distinguish between these two types of activity, we focused on the central ROI (Fz, Cz, Fc1, and Fc2), as beta activity typically shows more posterior modulation. These channels were also used for subsequent spindle and coupling analyses. To calculate the time-frequency representation, we band-pass filtered the EEG signal at 21 exponentially increasing center frequencies (ranging from 1 to 40 Hz), each with a bandwidth of 10% around the center frequency bands. The resulting time series were Hilbert-transformed, epoched around stimulus onsets (from −1 to 2 sec) (see Fig. 2A), baseline-corrected by subtracting the average activity in the 200 msec prior to stimulus onset from each time point, and averaged across all trials. The resulting time series were z-scored with respect to a baseline period (−200 msec prior to stimulus onset) for a better comparison across frequencies. For this we firstly calculated the mean and standard deviation of time series in the baseline period and then subtracted the mean from the time series and divided them by the standard deviation at each time point. The following z-score analysis with baseline correction was computed with the same procedure. The time-frequency representation confirmed clear spindle activity. We then extracted EEG-spindle activity in the frequency band showing highest amplitudes. We band-pass filtered the EEG signal between 14 and 18 Hz. Subsequently, we determined the analytic amplitude A_(t)_ by Hilbert-transforming the signal maintaining the same baseline correction settings. The resulting spindle activity was averaged across all trials separately for rest and PA, then we z-scored the spindle activity with a baseline correction period from −200 msec to stimulus onset (see Fig. 2B). To investigate the disparity in spindle activity between the rest and PA sessions over different time courses, we conducted a t-test to analyze the difference between the two datasets, using the averaged spindle activity between 1 and 2 sec.

### Ripple activity

Ripple events were detected in the ongoing MEG signal in the following way^17,18,39^. Ripples were first observed in rodent hippocampus which occurred around 100–200 Hz^13^, while ripples in humans, which share many characteristics with it, occur at frequencies around 80-150 Hz^14,15^. Hence, the MEG signal was band-pass filtered between 80 and 150 Hz. We obtained the analytic amplitude A_(t)_ by applying a Hilbert transform to the filtered time series. To avoid that potential filtering artifacts affect ripple detection, A_(t)_ was reset to zero during the initial and final 100 msec of each trial. To establish a distribution of ripple events throughout the entire experiment, we concatenated all trials and computed the z-score Z_(t)_ separately for each MEG sensor. Ripple events were identified as peaks in the Hilbert signal that exceed 2.5 SD^39^ above mean, extend over 20 msec up to 500 msec (equivalent to 3 cycles at 150 Hz and 40 cycles at 80 Hz), and were separated by at least 20 msec. Events exceeding 9 SD above mean were excluded. We then retained only ripple events with at least three peaks that exceeded 2.5 SD above the mean, with at least one peak designated as dominant (prominence ≥ 20% above neighboring peaks).

### Time frequency representation of ripple events

We tested whether ripples in MEG resemble the time-frequency representation in intracranial studies^17,18,39^ in two steps. First, we calculated the grand average ripple shape. To this end, we epoched the raw MEG signal (filtered between 1 and 200 Hz) around the ripple peak events. Within subjects we averaged all resulting ripple-centered time series leading to a ripple shape for each subject (see Fig. 3A). Second, we calculate the time frequency representation by band-pass filtering the MEG signal at 38 exponentially increasing center frequencies (between 1 and 200 Hz) each with a band width of 10% around the center frequency bands. We obtained the analytic amplitude A_(t)_ by Hilbert-transforming the filtered time series. Then we epoched the frequency specific A_(t)_ around ripple events (see Fig. 3A) and averaged across epochs. The resulting time series were z-scored as to a baseline period (−1.0 and −0.2 sec) before the ripple event.

### Frequency distribution of ripples

First, we determined the topographical distribution of ripple events across the entire experiment. For each sensor, we calculated the ripple likelihood as the average number of ripples across all trials leading to ripple rate for each of the 102 MEG sensors and each participant. We tested whether the distribution of ripples differed between PA and rest (see Fig. 4A). Specifically, we tested whether the topographical ripple distribution becomes more stable across subjects with PA. To do this, we utilized the individual vectors of the ripple likelihood distribution across the 102 MEG sensors for each participant, both during PA and rest. We then correlated the likelihood distribution between all possible pairs of participants. This resulted in 210 correlation values both for rest and PA. The resulting correlation coefficients were converted into a metric distribution by calculating its inverse hyperbolic tangent and compared using a t-test. In the following steps, we limited the analysis to the MEG sensors with the highest number of ripples, which were four sensors positioned bilaterally covering the MTL region. The ripple peak time points were used to calculate the grand average ripple shape and the time frequency representation, and coupling with EEG spindle activity. For all the calculations, we chose a two-second window centered around the ripple events (from −1.0 to + 1.0 sec).

### Ripple time series

In the next step, we defined the time course of ripple events around stimulus presentation (see Fig. 4B). We summed the number of ripples detected across the four most informative sensors and all trials in each participant. This resulted in a discrete time series of zeros and ones (indicating a ripple event) for each participant. We then convolved the resulting time series in the following manner. Within a moving time window of 200 msec, we summed ripple events. This yielded a continuous time series ${{\rm{R}}}_{({\rm{t}})}$ for each participant. Then, we standardized ${{\rm{R}}}_{({\rm{t}})}$ (z-score) according to baseline activity.

### The correlation of ripples with memory capacity

Next, we tested whether the ripple frequency predicts individual hit rate. The ripple likelihood time series showed a continuous decrease over one sec after stimulus onset with a following rebound. Therefore, we tested whether the initial decrease or the subsequent rebound is predictive of differences in performance. For each participant, we determined the mean ripple likelihood from 0 to 1 sec and from 1.5 to 2.5 sec after stimulus onset (see Fig. 4B). To investigate whether ripples correlated with individual performance, we averaged target detection performance across all blocks and physical activity sessions (rest and PA). We then determined the rank of performance across all participants and correlated this with the rank of individual ripple likelihood using Pearson correlation. In the next step, we investigated whether the number of ripples provided information about the number of items that need to be retained in working memory. First, we performed a one-way ANOVA to examine the differences in the number of ripples between the N-back conditions. Second, we asked whether inter-individual differences remain consistent across N-back conditions. That assesses if subjects with a higher number of ripples in one condition also have a higher number of ripples in another condition. To test this, we computed Pearson correlations of ripple counts across participants between different N-back blocks (2 vs. 3, 2 vs. 4, and 3 vs. 4), applying Bonferroni correction for multiple comparisons. Additionally, we correlated the ripple frequency in different N-back conditions with N-back (2-3-4, see Fig. 4C, D), to estimate the linear association between ripple frequency and increasing task demands. We employed linear regression in our analysis to examine the three conditions, as it offers several methodological advantages in this context. Here it was important to model the continuous relationship between the conditions and the dependent variable, which can be better captured by linear regression. Another advantage of linear regression is that it allows us to investigate an interpretable relationship between the conditions and the dependent variable without strictly adhering to the assumptions of variance analysis. By using linear regression, we were able to model potential interactions or trends between the conditions and estimate the influence of each condition on the dependent variable in a continuous framework. For each participant, we determined the mean ripple events separately for each N-back condition. We then correlated these three resulting values with the 3 N-values separately for the rest and PA session. As a result, we obtained Pearson’s r value for each participant, both for the rest and PA session. These values were translated into a metric measure by calculating their inverse hyperbolic tangent and were compared between rest and PA using a t-test across participants.

### MEG-Ripple to EEG-low-frequency coupling

#### Ripple centered EEG epochs

Ripple and spindle activity have been observed in the human medial temporal lobe and ripple-spindle coupling is described as signature of memory consolidation in rodents^40,41^. Analogous findings in humans can be found during sleep^42^. Here we tested whether we could find a similar coupling pattern during wakefulness with non-invasive measurements. In the first step, we tested whether a modulation of EEG activity occurs simultaneously with ripple events (see Fig. 5A right). Since there could be coupling between ripples and lower frequencies, where the spindle frequency acts as a harmonic multiple. We investigated the ripple centered low frequency band rather than only spindle frequency band. In such cases, we would be investigating harmonic oscillations rather than direct ripple to low-frequency coupling. The fact that spindle activity is observed despite the broad frequency range emphasizes the robustness of our analysis. Although lower frequencies typically exhibit higher amplitudes, oscillations in the spindle band are the ones that emerged as significant. We epoched the filtered EEG signal (1–25 Hz) at the central channels around the ripple events. We identified ripples as outlined above and segmented the EEG data into epochs of length 2 sec (−1 to 1 sec) centered to the peak of the ripple. Resulting time series for each ripple event and each channel were averaged within subjects. This yielded a time series RS_(t)_ for each participant. We then standardized RS_(t)_ (z-score) according to baseline activity. First, we calculated the mean and standard deviation of the RS_(t)_ in the baseline period. Here, we chose the time interval between −1.0 and −0.5 sec before the ripple event as the baseline since we expect spindle events to be longer in duration than ripple events. Finally, we subtracted the mean from the RS_(t)_ at each time point and divided the RS_(t)_ at each time point by the standard deviation. We compared the spindle amplitude of the ripple centered EEG at each time point against 0 using a t-test. In the next step, we constructed surrogate EEG signals which were also tested against 0. The surrogate signals were constructed by centering the EEG signals to random time points matching the number of ripples in each subject. In this analysis, we shifted the ripple time series for each participant over time. This allows for maintaining both the number of ripple events and their intervals consistently. With these new surrogate ripple events, we repeated the analysis described above (see Fig. 5A left).

#### Ripple spindle coupling

In the next step, we investigated whether ripples are coupled to a specific low-frequency phase (see Fig. 5B). Phase-amplitude cross-frequency coupling (PAC) is a mechanism that has been proposed to coordinate the timing of neuronal firing within local neural networks^43^. We utilized conventional cross-frequency coupling metrics^43,44^ to test for coupling of ripple events to low frequency bands in EEG channels. Generally, we determined at which phase of the low-frequency activity ripples most frequently occur. To do this, we initially determined the phase angle at each time point for 20 frequency bands ranging from 1 to 20 Hz each. First, we band-pass filtered the EEG signal at these center frequencies each with a width of 2 Hz. We then extracted the instantaneous phase information using the Hilbert transformation. We calculated the instantaneous phase of the low frequency activity for each EEG channel time series. We divided each low frequency cycle separately in 50 equally spaced bins ranging from –π to π and summed the number of ripples within a 45-degree window centered on every phase bin^45^. The resulting ripple event histograms – each containing 50 values – were averaged, separately for each low frequency band (see Fig. 5B right). We then averaged the dominant phase across different subjects with (circ_mean in Circular Statistics Toolbox) and compared the phases with parametric Watson-Williams multi-sample test (circ_wwtest in Circular Statistics Toolbox, see Fig. 5B right lower).

### Temporal coordination of ripples with spindle events

In the previous steps, we examined whether ripple peaks are generally coupled with spindle activity. However, spindle activity is not a uniform oscillation but is characterized by specific high-amplitude bouts^35^. In the next step, we examined whether ripples are temporally locked to spindle events by comparing the frequency of ripples outside and within spindle events. We defined spindle events as follows: first, we filtered the EEG signal in the spindle frequency (13-20 Hz) and obtained the analytic amplitude A_(t)_. The resulting time series was z-scored, and peaks with z > 4 were detected. Note, that the peak of A_(t)_ is not necessarily the peak/trough of the spindle oscillation. Given that ripples are aligned with a specific phase (see Results) of the spindle activity we centered spindle events to the troughs of spindle oscillation. Hence, we epoched the data (−2 to 2 sec) around each detected peak in A_(t)_. We then detected the trough of the spindle oscillation closest to the peak of A_(t)_. This time point was used as the center of the spindle event. To determine spindle duration, we first applied an envelope to the spindle band-filtered data. We then identified envelope peaks that exceeded a threshold set at the mean plus one standard deviation of the spindle event. The start time of a spindle was defined as the valley preceding the first envelope peak, and the end time as the valley following the last peak. Subsequently, we epoched the ripple time series around these spindle events. We then tested when ripples occurred relative to individual spindle events. Here, we defined the ‘spindle-out’ interval, which occurs outside spindle events, as the 1-second periods before and after each spindle event. The ‘spindle-in’ interval was defined based on the duration of the spindle event itself. Ripple density was calculated by summing ripple occurrences across four sensors positioned bilaterally covering the MTL region, averaging them across spindle events, and then dividing by the number of sample points. For each interval, we computed the average ripple count across all spindle events for each subject (see Fig. 5C). This results in a ripple event rate both for the within and outside interval for each subject. This was done separately for the PA and rest session. The resulting ripple event rates were then compared using a t-test in each session. Finally, we tested when ripple likelihood peaks relative to spindle peak events (see Fig. 5D). A peak of ripple likelihood is shown shortly after the spindle onset. First, we summed ripple epochs across all spindle event peak for each participant, creating a time series of ripple likelihood around the spindle event peak for each participant. These individual time series were then averaged across subjects. This averaged ripple rate was compared against a surrogate distribution. To construct this distribution, we shifted the summed ripple epochs around spindle event onset for each participant over time and averaged the resulting time series across participants. This process was repeated 1000 times, resulting in 1000 surrogate ripple event series. We determined the likelihood of ripple events at each time point relative to the surrogate distribution at each time point by estimating the probability density functions (pdf.m in Matlab). This yielded a probability value p for each time point around the spindle event.

### Reporting summary

Further information on research design is available in the Nature Portfolio Reporting Summary linked to this article.

## Supplementary information

Transparent Peer Review file

Reporting Summary

## Data Availability

All data supporting the results presented in the results section and figures can be accessed on github (https://github.com/SDuerschmid/PhysicalExerciseWorkingMemory) upon publication.
