# Supplementary material for: Physical activity simultaneously improves working memory and ripple-spindle coupling
Source: Commun Biol. 2025 Aug 8;8:1187. doi: 10.1038/s42003-025-08618-3 (PMC12334720; doi:10.1038/s42003-025-08618-3)
Supplement: Supplementary file 1 — Transparent Peer Review file [file 42003_2025_8618_MOESM1_ESM.pdf]

nature portfolio

Peer Review File

Physical Activity Simultaneously Improves Working Memory and Ripple-Spindle Coupling

Corresponding Author: Dr Stefan Dürschmid

Parts of this Peer Review File have been redacted as indicated to maintain the confidentiality of unpublished data.

This file contains all reviewer reports in order by version, followed by all author rebuttals in order by version.

Version 0:

Reviewer comments:

Reviewer #1

(Remarks to the Author)
1. Physical exercise (PE) is a broad concept. How do the authors define PE? In this study, the authors utilized a 2-minute pedal exercise. What type and intensity of exercise does this pedal exercise represent? What are the differences between this brief step exercise and other forms of exercise, such as resistance exercise and aerobic exercise? Moreover, we briefly reviewed the references ([1]-[3]) mentioned in the introduction, and they do not seem to adequately support the authors' assertion that a "brief sessions of PE enhance WM performance"(Line 52). Could the authors provide further clarification and justification for this statement?

2. The title and conclusion stated that "physical exercise improves working memory by ripple-spindle coupling" indicating a relationship between working memory performance and ripple-spindle coupling. However, there was no evidence in this study demonstrating this relationship. In the results, we only found that PE simultaneously altered WM performance and coupling, but this does not support this conclusion.

3. The authors conducted multiple 2-minute pedal exercise sessions. As the number of exercise sessions increases, did the intensity of this exercise also increase, which could be measured by objective indicators? It is possible that the WM performance and the related EEG/MEG activity changes were also influenced by the exercise intensity.

4. Given the involvement of multiple comparison corrections, please provide more details on the post-hoc comparisons in the "Statistical Analysis" section.

5. The authors reported t-test results in this sentence (Line 102-105), but the text and statistic results did not seem to correspond. Which two variables were being compared in the t-test mentioned in this sentence?

6. In Line 390-391, the author performed a Pearson correlation on the ranks of two sets of data. Please check whether this is correct.

7. In Line 393-397, the authors seemed to perform a Pearson correlation between ripple frequency and the N-back task conditions (i.e., 2, 3, or 4). However, this seems inconsistent with the requirements of Pearson correlation analysis for variable types. Could the authors provide details on how this correlation analysis was conducted?

8. Many symbols such as asterisks were presented in the figures, but they were not explained in the figure captions.

9. In the discussion section, the authors cited evidences that short bouts of exercise enhanced memory performance. However, there is curiosity about how this type of exercise specifically affects spindle activity, ripple activity, and ripple-spindle coupling to improve memory capacity. Could the authors further discuss this question?

Reviewer #2

(Remarks to the Author)

This study examined the association between working memory (WM) performance and physical exercise (PE) as well as spindle-ripple coupling measures. The authors reported that the WM performance was greater in the PE session compared to the rest session and also found a positive correlation between the performance and ripple rate. They further claimed that the spindle-ripple coupling was stronger in the PE session. The topic is interesting, but there are several major issues with the results presented in this study.

Major points

1) Spindles are spontaneously generated in the thalamocortical network during NREM sleep and the transmission of sensory input is also suppressed during sleep spindles. Unlike hippocampal sharp wave ripples and cortical up and down states, there is no compelling evidence for the existence of spindles during awake state. However, in this manuscript, the authors focused on the spindle activity after the onset of the stimulus. It seems that what they reported as the spindle activity was only the increase in the beta power during awake state, not spindles (in other words, the increase in the beta frequency band doesn't necessarily indicate the existence of spindle events with the properties and mechanism similar to the sleep spindles). In line 190, the authors referred to reference 8 and stated that "The authors found spindle amplitude during WM task in alignment with our study demonstrating PE improved working memory performance and increased spindle activity". However, in this reference the relationship between the alpha/beta frequency range (6–20 Hz) during encoding and sleep spindles was investigated and the spindle activity during task was not examined.
The authors showed raw traces during ripples but no exemplary detected spindle event was shown. Especially, in their spindle detection method, the duration, topographies and the frequency range of spindles they used/reported is not consistent with the ones widely considered for the sleep spindles:
i) The authors considered all the events with the amplitude in the spindle frequency range exceeding the 4 z-score threshold value as the spindle event regardless of the duration of the event. However, the spindle duration should be longer than 0.5 s and hence a large portion of the events that the authors detected as spindles were shorter than the minimum spindle duration. Moreover, events longer than 3 s should also be excluded.
ii) While (fast) spindles mainly occur in centro-parietal areas (for example see doi: 10.1523/JNEUROSCI.2604-11.2011 and 10.1152/physrev.00042.2018), the authors reported that the spindle activity was maximum at fronto-central electrodes and stated that this topographic location was consistent with the ones reported in reference 8. However, in this reference, it has been clearly reported that the spindle activity was most pronounced in centroparietal areas.
iii) The Authors considered different frequency bands for spindles (14-18 Hz for figure 2, 14-16 Hz for figure 5B, 13-20 Hz for spindle detection, line 446) but none of them is consistent with the established spindle frequency range (10-16 Hz). In line 325, based on the time-frequency representation shown in figure 2A, the authors claimed that "The time-frequency representation confirmed clear spindle activity", however, the frequency range with increased power spans from 14 Hz up to 20 Hz which is higher than the spindle frequency range. On the other hand, there is a power reduction in a frequency range corresponding to the spindle frequency range (10-16 Hz) during a 1 s time window after the stimulus.
2) Another main concern is that the spindle-ripple coupling analyses have not been conducted or reported appropriately as is explained below:
i) The authors do not directly compute the spindle-ripple coupling strength. There are commonly used phase amplitude coupling (PAC) methods that measure both the strength and phase of the coupling between spindles and ripples (for example see the PAC method used in doi:10.1038/nn.4119 which measures the strength and phase of coupling of the ripple amplitude to the spindle phase within spindle events). Since spindles are long events that can be accompanied by several ripples, it is more accurate to use these more detailed PAC methods instead of simply computing the spindle power around each ripple event. In spite of this, it is not clear for me that why the authors reported "low frequency" amplitude (1-25 Hz) around the ripple event and not the power in the spindle frequency range (Figure 5A).
ii) It is more accurate to conduct the analyses of figure 5B only during spindles, since the phase values at the times with low amplitude in the spindle frequency range are meaningless (see for example the method used for phase-locking analyses in doi: 10.1111/ejn.14906).
iii) In another analyses (Figure 5C), the authors aimed to compare the number of ripples co-occurring with spindles with the isolated ripples. However, the time window they used to define these two different types of ripples is too short (-0.1 to +0.1 s for inside spindles and -0.1 o -0.3 for outside spindles) and is fixed for different spindles with different lengths. Hence their method does not correctly separate the ripples coupled to the spindles and the uncoupled ones. Since the spindles are events longer than 0.5 s, the time window to define the co-occurrence of the ripples with spindles should be longer than 0.5 s. In addition, as the length of the spindles is highly variable (from 0.5 to 3 s), it is more accurate to use different time windows for different spindles by defining the coupled ripples as the ones that occur between the onset and offset of spindles (for example see the method used in doi:10.7554/eLife.48881).
iv) The authors claimed that "A peak of ripple likelihood before the respective spindle peak event might indicate that ripples trigger spindles" (line 462) and hence computed the ripple likelihood locked to the spindle peak. However, to investigate whether the ripples can trigger the spindle events, the ripple likelihood should be computed locked to the spindle onset and not its peak.
3) For time-frequency representation (TFR), why don't the authors use the more accurate and widely used TFR methods like wavelet or multitaper (mtmconvol function of the FieldTrip toolbox, see for example: doi:10.1038/nn.4119)? The robustness of results to a different TFR method should be checked.
4) To find the association between ripple density and different N-back conditions, the authors conducted the correlation analyses between only 3 data points and separately for the rest and PE sessions which is not accurate. Instead, the comparison analyses like ANOVA should be conducted.
5) For the clusters shown in Figure 2B and Figure 4B, correction for multiple comparisons should be applied using the cluster-based permutation procedure (for example see doi:10.1038/nn.4119 or 10.1016/j.neuron.2017.11.020).

Minor points

1) While the authors reported the correlation between ripple activity and performance, the correlation between spindles/spindle-ripple coupling and performance has not been reported.
2) Is there any difference in the ripple/spindle activity after target versus standard stimulus?
3) It is not clear for me why the correlation coefficients have been transformed to inverse hyperbolic tangent.
4) Line 157: the unit of phase is not reported.
5) Lie 316: “we band-pass filtered the MEG signal at 21 exponentially”. MEG or EEG?
6) Overall, while the method in the manuscript text is clearly explained and well written, the required information for each figure has not been provided clearly in the figure or its legend. For example:
In all the figures, the number of data points that have been used to compute the average values is not reported.
Figure 2A: what measure is shown in the left panel?
Figure 2: the definition of the zero time should be stated in the figure legend.
Figure 3A: what do the two blue and red colors represent? There is no legend.
Figure 3B: the time scale has not been shown.
Figure 4A top: no color bar is shown.
Figure 4C: it is better to report ripple density than numbers.
Figure 4 and 5: the legend explaining the blue and red colors is not shown.
Figure 5A: The frequency band of “low frequency amplitude” that is shown in the y axis is not mentioned in the figure. In the text, the frequency band is written to be 1-25 Hz which does not correspond to what is commonly considered as low frequency in the EEG signal. As was mentioned above, it is also not clear why the authors computed the amplitude in this frequency range instead of the frequency range of spindles.
Figure 5D: What does the vertical red line represent?

Reviewer #3

(Remarks to the Author)
The authors provided evidence regarding the effects of physical exercise (PE) on brain rhythms, specifically focusing on spindle and ripple activity as foundational elements of working memory (WM). Using electroencephalography (EEG) and electromyography (EMG) devices, the study first demonstrated that PE leads to significant improvements in WM performance, as assessed by the N-back task. Furthermore, the enhancement in WM was associated with increased ripple activity within the medial temporal lobe, particularly in coupling with the spindle. These findings contribute to our understanding of the functional role of ripple-spindle coupling in the context of PE-enhanced WM, underscoring the neurophysiological impact of PE on cognitive function.

Overall, the manuscript is well-written, and the results are presented well. The study contributes to the field by elucidating the relationships between PE, WM, and spindle-ripple coupling. However, the introduction would benefit from descriptions of current memory theories, the relationship to PE, and the relationship between spindle and/ or ripple activity during PE. Further, the description of the PE used in the study lacks details regarding the methods used in the study. Specifically, there is ambiguity regarding the control of exercise intensity (which appears to be of moderate intensity). Additionally, since there was no physiological measure, for comparison with other studies, references or wheel parameters should be given. I assume the exercise was aerobic? Clarifying these aspects is important for accurately interpreting the study's findings and implications.
The following are my concerns regarding the study:

Comments:

Introduction:
Line 58: Please note the frequency range of spindles, like ripples.
Line 68: The phrase “PE enhanced detection” is unclear. Does this refer to an improvement in behavioral performance on the working memory task, or is it describing the increase in ripple activity mentioned in the following sentences?

Results:
Line 111: Typo? --“ripple frequency = .125Hz”. Is it “ripple rate” meant?

Methods:
Line 230 (Participants): Was there any subject dropout? Please discuss why a gender balance wasn’t used.
Line 238 (Procedure): Please provide detailed information on the display parameters used for stimulus presentation, including the resolution and refresh rate of the display, the size/visual size of the stimulus, and any other relevant details regarding the visual presentation setup.
Line 248: For the rest condition, please explicitly state that the subjects were sitting on the pedal trainer during the EEG/ MEG measurement.
Line 291: Please define what is t=0 in your study. Is this reference point aligned with the onset of the stimuli?
(Recording): Please provide the topography of the EEG. Some electrodes are more prone to muscle artifacts. Thus, the electrodes depicted should be given.
Line 308 (Behavioural performance): Please clarify the method used to address violations of the sphericity assumption in

your ANOVA analysis.
Line 327: The spindle frequency range is given as 14-18 Hz. Can you please provide a reference for looking at this range? In sleep, the typical (fast) spindle frequency range is 12-16 Hz. Aside from the frequency range, can you elaborate on any further criteria used for spindle detection?
Line 332: Running t-tests were used to compare average values at every 0.1-second interval following stimulus onset. Did the statistical results underly any testing for multiple comparisons, e.g., using a false discovery rate method? Give information on these results in the figures, e.g., in Fig. 2 B.

Legends of the figures should be self-explanatory without the text. Fig. 1 B, C (right), as well as Fig 3B: Please add the meaning of the x-axis. Give the meaning of the asterisks and symbols in the legends of all figures. For instance, in Fig. 1 B, right a strange symbol is used.

Version 1:

Reviewer comments:

Reviewer #1

(Remarks to the Author)
The authors have made substantial improvements.

Reviewer #2

(Remarks to the Author)
Major points
The authors did not address most of my major comments adequately in the revised manuscript.
-The main results of this manuscript are based on "awake" spindles, however, as I explained in details previously, there is no compelling evidence of the existence of spindles during the awake state. In the revised manuscript, the authors stated that "it has been reported that the neural sink and source of wake spindles are similar to those of sleep spindles (20)". However, in this old reference (20) which is the only reference provided by the authors for the existence of "wake" spindles, only the "sleep" spindles with "high voltage spike-and-wave patterns (HVSs)" were compared. The authors conducted experiments to compare the properties of sleep and "wake" spindles and stated that both spindles mostly occur in the fronto-central region. However, the topographical distribution shown in figure R3 D and R4 D are not similar, and also there is no color bar in these figures to compare the spindle density between them. More importantly, using a simple method of detection to find the high amplitude activity in 13-20 Hz from the EEG in a brain state does not provide compelling evidence for the existence of the spindles with the known thalamocortical mechanisms in that state. Also, the example of the detected spindle shown in figure 6 does not look like a typical spindle in terms of duration and waveform shape.
There are several concerns with the spindle detection method that was used in this manuscript which have been mentioned previously but the authors did not adequately address them in the revised manuscript or the response letter:

1- Spindle frequency: They authors called very different ranges of frequencies as the "wake" spindles but none of them corresponds to the mostly used frequency range for spindles. For example, the authors stated that "We found a consistent temporal pattern across both sessions, with spindle activity decreasing immediately after stimulus onset, followed by a subsequent increase at fronto-central EEG electrodes". However, the frequency range of the decrease and increase of the activity are very different. The reduction is concentrated around 9-10 Hz but the increase is from around 14 Hz up to more than 20 Hz. The authors called all of this wide range as "wake spindles" without any further evidence.
In response to my previous comment, the authors stated that "Additionally, we compared the frequency amplitude at the spindle peak and found that the main frequency range was similar between sleep and wake spindles (see Figure R7)". However, this similarity is due to using the same frequency range of 13-20 Hz to detect sleep and wake spindles and does not directly address my concern.
For spindle-ripple coupling, the authors considered a wide range of frequencies from 1 to 25 Hz. To address this concern, in the response letter, the authors wrote: "Our analysis, which includes a broader frequency range, highlights the robustness of spindle activity despite higher amplitudes at lower frequencies. Notably, oscillations in the spindle band emerge as significant.". However, the peak and through of the modulated low frequency activity around ripples are of the order of 10 ms apart and there are no oscillations in the spindle frequency band. In figure R5 where they used the spindle frequency range to determine the spindle modulation around the ripples, it seems that there is no significant difference between the PA and rest conditions.

2- Spindle duration: It is very important to consider the minimum duration of 0.5 s for the spindle detection (similar to almost all the previous articles that detected spindles), otherwise, as is also observed in figures R3 & R4 A, a large portion of the events would be very short ones which are not necessarily related to the spindle activity. To address this previously mentioned comment, the authors stated in the response letter: "To address this, we analyzed the frequency distribution of spindle lengths and found that the events averaged over 500 ms in duration". However, an average value over 0.5 s is not sufficient and there should be no event below 0.5 s.
3- Spindle topographical distribution: The authors claimed that (fast) spindle activity was typically seen in fronto-central regions similar to the previous observations and they referred to only one reference (12). However, in this paper (reference 12), consistent with many other papers (for example see doi:10.1523/JNEUROSCI.2604-11.2011 and 10.1152/physrev.00042.2018) the authors clearly stated that "At the group level, spindles and SOs showed the established

prevalence over centro-parietal and frontal areas, respectively". In figure S2 of this paper it has been shown that the fast spindle activity (density) is dominant across centro-parietal regions. The "slow" spindles occur mostly at fronto-central regions; however, the frequency range that the authors have considered for the spindle (13-20 Hz) is very different from the slow spindle frequency range (8-12 Hz).
-The authors found no direct evidence for different strengths of spindle-ripple coupling in the PA condition compared to the rest condition and hence it can not be concluded that the PA is associated with enhanced spindle-ripple coupling. However, they stated this claim in several sections of the manuscript:
abstract: "These findings reveal that PA enhances WM by coordinating ripple-spindle
Interaction", introduction: "Critically, PA increased coupling of MEG-ripples to EEG-spindle activity and also enhanced WM performance.", discussion: "Our results indicate that short-term PA enhances spindle-ripple coupling, contributing to improved memory formation."

Other points
-The authors investigated the ripple activity after the onset of stimulus. However, ripples are believed to occur mostly during the rest state not during the stimulus. Hence the decrease in the ripple activity they found after the stimulus onset is not novel.
-In the discussion, it has been written: "We observed spindle activity in both PA and rest sessions, topographically overlapping with sleep spindle findings in previous studies12. Notably, the amplitude of sleep spindles increased after the working memory task facilitating memory consolidation". However, no measure of memory consolidation/retention has been conducted in this study and the behavioral performance has measured only once.
-I am not convinced with authors explanation about using correlation analyses between only 3 data points instead of ANOVA to find the association between ripple density and different N-back conditions.
- The time scale in figure 5A is missing.
-Figure 6 was not cited in the text.

Reviewer #3

(Remarks to the Author)
The authors have addressed my comments nicely. I have no further comments.

Open Access This Peer Review File is licensed under a Creative Commons Attribution 4.0 International License, which permits use, sharing, adaptation, distribution and reproduction in any medium or format, as long as you give appropriate credit to the original author(s) and the source, provide a link to the Creative Commons license, and indicate if changes were made.
In cases where reviewers are anonymous, credit should be given to 'Anonymous Referee' and the source.
The images or other third party material in this Peer Review File are included in the article's Creative Commons license, unless indicated otherwise in a credit line to the material. If material is not included in the article's Creative Commons license and your intended use is not permitted by statutory regulation or exceeds the permitted use, you will need to obtain permission directly from the copyright holder.
To view a copy of this license, visit https://creativecommons.org/licenses/by/4.0/

We are grateful for the encouraging reviews and the helpful suggestions made by the three referees, which allowed us to improve the manuscript. Below, please find our response to each of the reviewers' comments. New text we added to the manuscript will be underscored in our replies. Note, the reference numbers do not match between the reply letter and the manuscript, as we did not use all the references in the reply letter.

Reviewers' comments:

1 (Remarks to the Author):

1. Physical exercise (PE) is a broad concept. How do the authors define PE? In this study, the authors utilized a 2-minute pedal exercise. What type and intensity of exercise does this pedal exercise represent?

Reply: We apologize for the lack of clarity in defining physical exercise (PE). PE is commonly described as planned, structured, and repetitive activity involving intentional physical effort[1]. However, this definition did not fully apply to our study. Our participants were instructed to pedal at a self-regulated, moderate pace, aiming for a speed that was neither too slow nor exhausting. The pedal trainer used in this study initially lacked a light barrier to measure pedaling speed. To address this limitation, a newer version of the trainer was developed, equipped with a light barrier for speed measurement. This was used to define the velocity the participants pedaled. We conducted a behavioral study measuring individual speeds of 29 new participants under identical instructions, as participants from the original study were unavailable for re-measurement. In this study, participants were instructed to pedal at a moderate pace for two minutes, and their speed was measured across four sessions. We analyzed individual speeds both within and across sessions. On average, participants maintained a moderate speed of approximately one step per second (see ***Figure R1***). Importantly, their speeds were consistent across sessions, as indicated by strong correlations between blocks (see ***Figure R2***). These findings demonstrate that individual pedaling speeds were stable over time under the same instructions, highlighting the reliability of the self-regulated, moderate pace.

To avoid confusion, we replaced 'physical exercise' with 'physical activity' throughout the text.

For example, the abstract now reads:

Spindle-ripple coupling enhances memory consolidation during sleep. Ripples, representing the compressed reactivation of environmental information, provide a mechanism for retaining memory information in chronological order and are also crucial for working memory (WM) during wakefulness. Brief sessions of <u>physical activity (PA)</u> are proposed to boost WM. In concurrent EEG/MEG sessions, we investigated the role of <u>PA</u> in WM performance and high-frequency-ripple to wake spindle coupling. Ripples, identified in MEG sensors covering the medial temporal lobe (MTL) region, predicted individual WM performance. Ripples were locked to robust oscillatory patterns in the EEG defined spindle band.

What are the differences between this brief step exercise and other forms of exercise, such as resistance exercise and aerobic exercise?

Reply: Aerobic exercise involves sustained, low-intensity activity fueled by oxygen, improving cardiovascular endurance, while resistance exercise uses weights to build strength and muscle size. Our activity—pedaling at a moderate speed of approximately 1 step/sec for 2 min (see ***Figure R1***) — resembled aerobic exercise in intensity[2] but lacked prolonged duration and external resistance, differentiating it from resistance exercise[3]. This moderate activity was chosen to minimize MEG artifacts. Similar moderate-intensity activities, like rodent wheel running, have been linked to enhanced hippocampal plasticity and memory improvements[4].

We added this to the method section. The new paragraph now reads:

<u>The pedal trainer was specifically designed in-house for MEG recording. All materials were carefully tested to ensure they contained no magnetic components. The device’s angle and height can be adjusted</u>

to suit each participant's proportions, and its design allows for independent forward and backward movement of both feet. Participants were instructed to pedal at a moderate, self-adjusted pace, similar to a walking motion. While this activity resembles aerobic exercise—defined as movement sustained by oxygen delivered through the blood to active muscles—it did not have the prolonged duration typical of aerobic workouts[2] This adjustment was made to minimize MEG artifacts, such as ECG interference or changes in skin impedance caused by sweating[5], which can affect EEG electrodes contact. Similar moderate-intensity exercises, such as wheel running in rodents, have been shown to enhance hippocampal plasticity and improve memory, linking our findings and previous animal research[6].

Moreover, we briefly reviewed the references ([1]-[3]) mentioned in the introduction, and they do not seem to adequately support the authors' assertion that a "brief sessions of PE enhance WM performance"(Line 52). Could the authors provide further clarification and justification for this statement?

Reply:

We appreciate your comments, which have prompted us to address this issue and improve the rigor of our discussion.

Our work aligns with studies demonstrating that acute cardiovascular exercise positively influence short-term memory in specific contexts. For example:

- **Sibley and Beilock (2007)**[7] reported improvements primarily in individuals with lower baseline memory scores.
- **Audiffren et al. (2009)**[6] and **Pontifex et al. (2009)**[8] found benefits only in specific memory domains, while **Davey (1973)**[9] noted enhancements specifically at moderate exercise intensities.
- Conversely, some studies report detrimental effects on short-term verbal-auditory memory under certain conditions[10,11] but these studies involve intense exercise.

Additionally, brief aerobic exercise is known to modulate neural network activity and improve bottom-up, stimulus-driven processes[6] and enhance functional connectivity in hippocampal regions after light-intensity cycling[12].

These studies highlight the nuanced relationship between exercise intensity, memory performance, and underlying neural mechanisms. We have revised the manuscript to integrate these findings:

Brief sessions of physical activity (PA) like walking[13,14] or cycling[12] enhance working memory performance[6–9,12] likely due to increased hippocampus-cortex coupling[12].

Thank you for pointing out these omissions. We believe the revised text more accurately reflects the existing research enhancing the context of our findings.

2. The title and conclusion stated that "physical exercise improves working memory by ripple-spindle coupling" indicating a relationship between working memory performance and ripple-spindle coupling. However, there was no evidence in this study demonstrating this relationship. In the results, we only found that PE simultaneously altered WM performance and coupling, but this does not support this conclusion.

Reply: Thank you for highlighting this. We acknowledge that we did not directly manipulate the coupling mechanism to affect memory. However, we observed that both behavioral performance and coupling changed after physical activity, with ripple rate correlating with memory performance. The increased spindle amplitude and stronger coupling after physical activity align with prior findings in both animal and human studies[15,16], suggesting that this coupling may enable communication between the hippocampus and neocortex, which may explain how physical activity enhances memory. While no study has definitively shown this, our work demonstrates that physical activity alters ripple-spindle coupling and links ripple rate to memory performance, providing a foundation for targeted interventions.

However, lacking a causal manipulation of WM we adjusted the title to: "Physical Activity Simultaneously Enhances Working Memory and Ripple-Spindle Coupling."

3. The authors conducted multiple 2-minute pedal exercise sessions. As the number of exercise sessions increases, did the intensity of this exercise also increase, which could be measured by objective indicators? It is possible that the WM performance and the related EEG/MEG activity changes were also influenced by the exercise intensity.

Reply: Thank you for this insightful question. We did not explore this aspect as we did not modulate intensity. In our study, participants moved at a moderate pace for 2 min, mimicking aerobic exercise intensity without the prolonged duration or resistance typical of aerobic or resistance training. This adjustment was made to minimize MEG artifacts, such as ECG interference or changes in skin impedance caused by sweating[5], which can affect EEG electrode contact. Moderate-intensity activities, such as wheel running in rodents, have been shown to enhance hippocampal plasticity and memory, aligning with prior animal research. The ***Figure R2*** has shown that participants maintained stable intensity (all correlation coefficients >.7) when instructed to move moderately, without increasing intensity across blocks.

4. Given the involvement of multiple comparison corrections, please provide more details on the post-hoc comparisons in the "Statistical Analysis" section.

Reply: We apologize for not previously clarifying the need for multiple comparison corrections. In our study, we conducted post-hoc comparisons on the behavioral data but did not report it in the methods section. In ***Fig. 1***, we reported significant differences in the 2- and 3-back conditions at a Bonferroni-corrected level ($p < .05/3$). The difference in the 4-back condition was significant only at an uncorrected level. We have now updated the Methods, Results, and the respective caption to clarify that all post-hoc comparisons across n-back conditions are significant at the corrected level, except for the 4-back condition.

The corresponding passage reads:

Performance declined with number of items to be held in working memory (H_2 = 80.07%; H_3 = 64.68%; H_4 = 52.12%). However, hit rate was higher after PA than after rest (H_{PA} = 68.51%; H_{rest} = 62.74%; t_{20} = 3.62; p = .0017) in 2 and 3 back conditions at the Bonferroni-corrected level (2-back: t_{20} = 2.78; p = 0.012; 3-back: t_{20} = 3.39; p = 0.003; 4-back: t_{20} = 2.21; p = 0.039, see ***Fig. 1B***). When we compared false alarm rates, no significant effect of PA was found ($F_{(1,120)}$ =1.56; p=0.214, see ***Fig. 1C***).

Furthermore, we have added to the Methods section that pairwise comparisons were Bonferroni-corrected:

We compared the impact of PA and rest on behavioral measures using a two-way ANOVA with the factors motion (physical activity vs. rest) and N-back (2-back, 3-back, 4-back) separately for DA, FA and RT. The performance of PA and rest sessions was compared using paired-sample t-tests at both the grand average level and for individual N-back levels. To account for multiple comparisons, we applied a Bonferroni-corrected significance level (p = .05/3) to define statistical significance.

We have also clarified the symbols in the figures, and the updated caption reads:

Fig. 1 | Behavioral Performance.

A: Task design. Subjects were instructed to rest or use a pedal trainer before the N-back task.

B: (left): shows that hit rate increased across all conditions. (right): Hit rate across different N-back conditions. (+: P<0.05, *: P<0.017, **: P<0.003, Bonferroni-corrected)

C: (left): Individual false alarm rate. (right): False alarms across different N-back conditions. Red indicates PA and blue rest session. (+: P<0.05, n.s.: not statistically significant)

5. The authors reported t-test results in this sentence (Line 102-105), but the text and statistic results did not seem to correspond. Which two variables were being compared in the t-test mentioned in this sentence?

Reply: The reviewer is correct that our original text did not clearly specify the variables compared. We have now clarified in the Results section that we compared spindle activity in the temporal interval between 1 and 2 sec between physical activity (PA) and rest.

The new paragraph now reads:

Spindle activity provides a temporal framework for ripple occurrence[19]. In the EEG, thalamic spindle activity is maximal at fronto-central electrodes[20]. We found a consistent temporal pattern across both sessions, with spindle activity decreasing immediately after stimulus onset, followed by a subsequent increase at fronto-central EEG electrodes (see ***Fig. 2A, left***), peaking at 1150 msec. Additionally, enhanced activity between 1 and 2 sec after stimulus presentation was observed in the PA conditions ($t_{20} = 2.20$; $p = 0.04$, see ***Fig. 2B***).

The Methods section now reads:

To investigate the disparity in spindle activity between the rest and PA sessions over different time courses, we conducted a t-test to analyze the difference between the two datasets, using the averaged spindle activity between 1 to 2 sec.

6. In Line 390-391, the author performed a Pearson correlation on the ranks of two sets of data. Please check whether this is correct.

Reply: Thank you for the suggestion. The methods section was intended for ***Fig. 4B***.

Here is the revised text:

For each participant, we determined the mean ripple likelihood from 0 to 1 sec and from 1.5 to 2.5 sec after stimulus onset (see ***Fig. 4B***). To investigate whether ripples correlated with individual performance, we averaged target detection performance across all blocks and sessions. We then determined the rank of performance across all participants and correlated this with the rank of individual ripple likelihood using Pearson correlation.

7. In Line 393-397, the authors seemed to perform a Pearson correlation between ripple frequency and the N-back task conditions (i.e., 2, 3, or 4). However, this seems inconsistent with the requirements of Pearson correlation analysis for variable types. Could the authors provide details on how this correlation analysis was conducted?

Reply: We appreciate the opportunity to clarify this point. Although Pearson correlation is typically used for continuous variables, it can also be appropriate for ordinal data, such as the N-back task levels (2-back, 3-back, and 4-back), when there is a natural order, and the intervals are assumed to be roughly equal. Given that N-back task difficulty increases monotonously, we treated these levels as ordinal data, potentially reflecting a linear trend. By correlating ripple frequency with these task levels, Pearson's correlation can provide meaningful insights into their association. Here, Pearson's correlation coefficient was used primarily as an estimator to compare the linear trend across conditions, which is valid if the data approximately follow a linear pattern. This approach allowed us to assess how ripple frequency varies with increasing task difficulty.

Here is the revised text:

In the next step, we investigated whether the number of ripples provided information about the number of items that need to be retained in working memory. We correlated the ripple frequency in different N-back conditions with N-back (2-3-4, see ***Fig. 4C, D***)，to estimate the linear association between ripple frequency and increasing task demands. We employed linear regression in our analysis to examine the

three conditions, as it offers several methodological advantages in this context. Here it was important to model the continuous relationship between the conditions and the dependent variable, which can be better captured by linear regression. Another advantage of linear regression is that it allows us to investigate an interpretable relationship between the conditions and the dependent variable without strictly adhering to the assumptions of variance analysis. By using linear regression, we were able to model potential interactions or trends between the conditions and estimate the influence of each condition on the dependent variable in a continuous framework.

8. Many symbols such as asterisks were presented in the figures, but they were not explained in the figure captions.

Reply: We apologize for the unclear definition. We have updated the figures in the manuscript, and here are the revised text and figures:

Figures

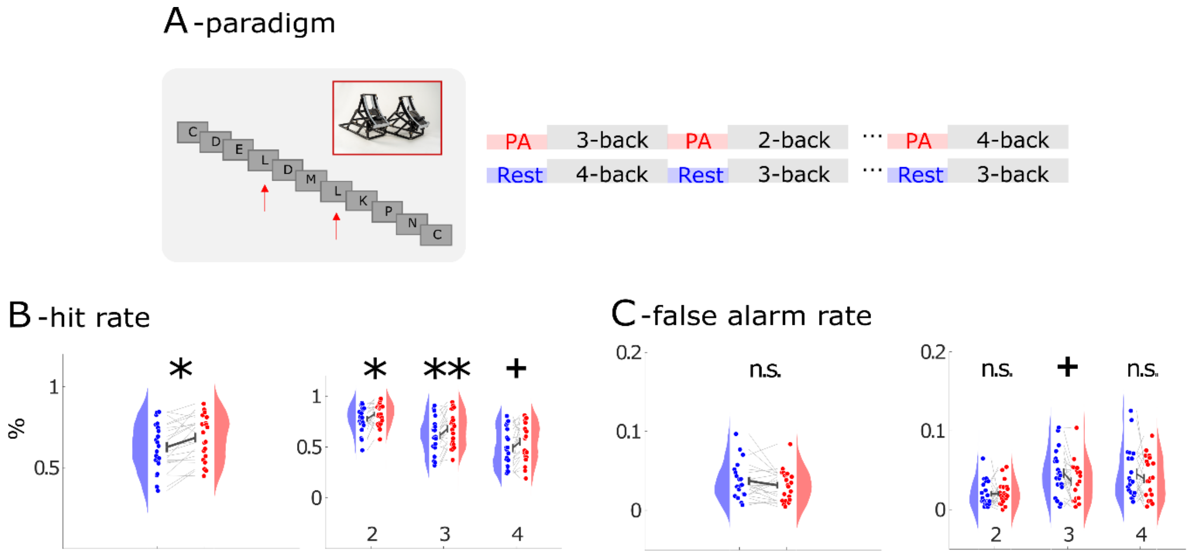

Fig. 1 | Behavioral Performance.

A: Task design. Subjects were instructed to rest or use a pedal trainer before the N-back task.

B: (left): shows that hit rate increased across all conditions. (right): Hit rate across different N-back conditions. (+: P<0.05, *: P<0.0167, **: P<0.003, Bonferroni-corrected)

C: (left): Individual false alarm rate. (right): False alarms across different N-back conditions. Red indicates PA and blue rest session. (+: P<0.05, n.s.: not statistically significant)

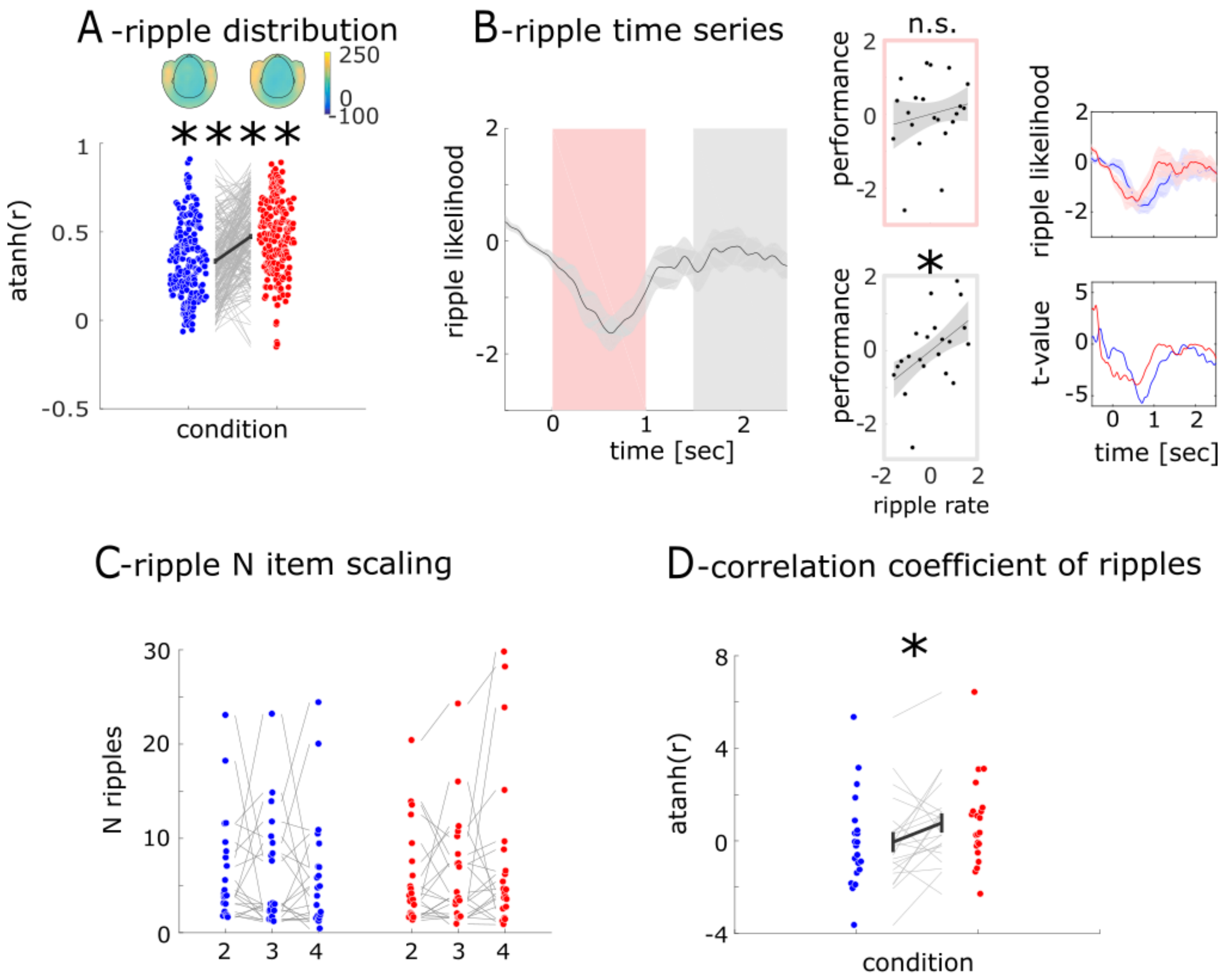

Fig. 4 | Ripple modulation.

A (upper): Topographical map of ripple likelihood across MEG sensors for rest (left) and PA (right). (lower): Correlation coefficients of ripple distributions between pairs of subjects. Higher correlations coefficients following PA indicate a more consistent spatial structure of ripples likelihood across subjects. (****: P<0.0001)

B (left): Time series of ripple likelihood. (middle): linear regression between individual memory capacity and ripple likelihood in segmented intervals. (right): The ripple likelihood time series from grand-averaged and different sessions, along with the corresponding t-values. (*: P<0.05, n.s.: not statistically significant)

C: Ripple count across N-back conditions displayed individually.

D: Correlation coefficients for rest (blue) and PA (red) calculated between the ripple likelihood and the 2, 3, 4-back levels. (*: P<0.05)

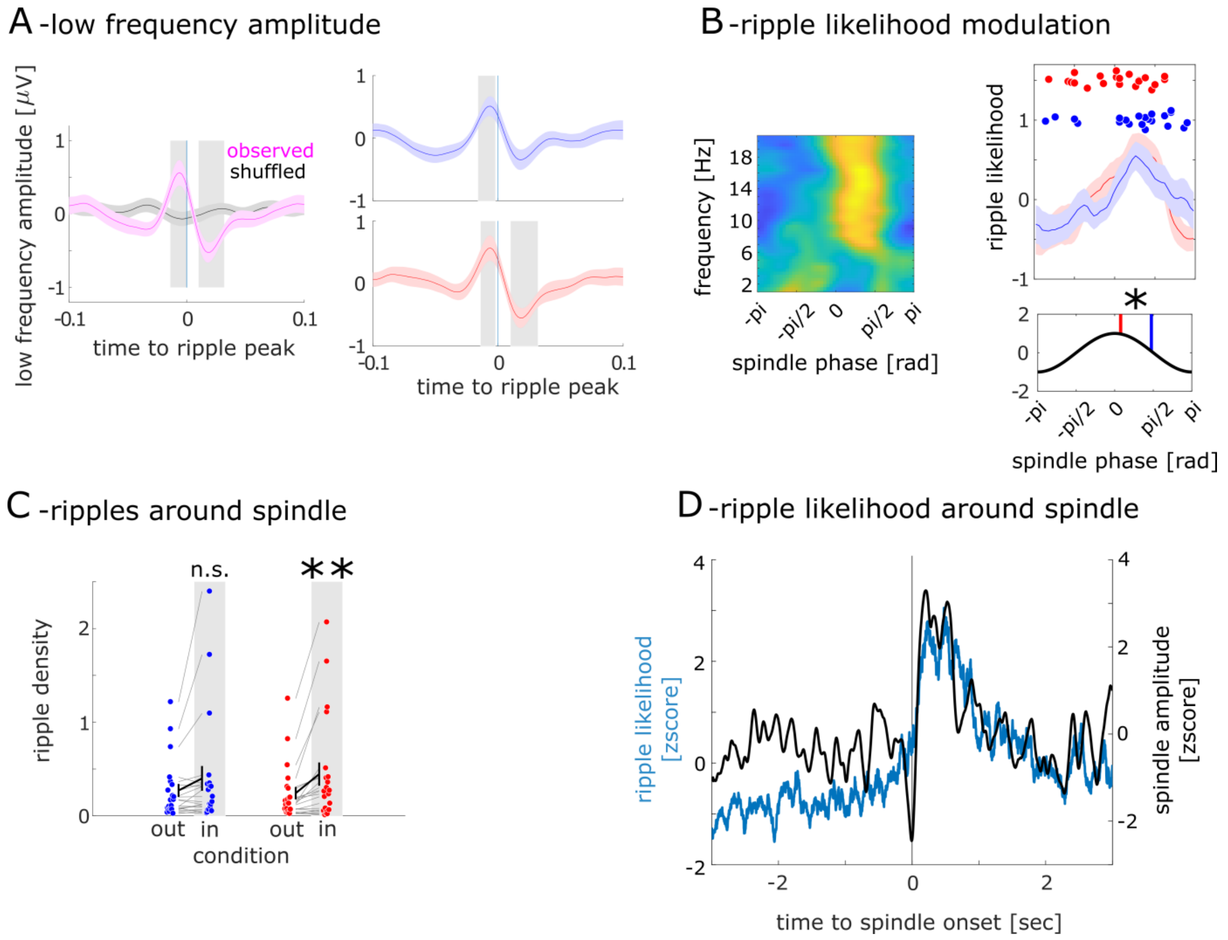

Fig. 5 | MEG-Ripple and EEG low-frequency coupling results.

A (left): Grand-averaged slow oscillations around the ripple peak compared to the shuffled peak. (right): The slow oscillations around the ripple peak from different sessions. The pink color represents the grand-averaged slow oscillations, the blue color represents the rest session, and the red color represents the PA session.

B (left): Phase distribution of low frequency around the ripple peak. (right upper): The phase distribution from 14-16 Hz from different sessions and the dominant phase from individual subjects. B (right lower): Average dominant phases across sessions. (*: P<0.05)

C: The ripple density during the 'in-spindle' and 'out-of-spindle' phases is plotted separately for the PA and rest sessions. Red denotes PA session data, while blue represents rest session data. (**: P<0.01, n.s.: not statistically significant)

D: Ripple likelihood around the spindle onset. The time 0 represents the spindle onset.

9. In the discussion section, the authors cited evidences that short bouts of exercise enhanced memory performance. However, there is curiosity about how this type of exercise specifically affects spindle activity, ripple activity, and ripple-spindle coupling to improve memory capacity. Could the authors further discuss this question?

Reply: We agree with the reviewer and added the following paragraph to the discussion:

Ripple activity has been reported to increase in the medio-temporal areas (hippocampus, amygdala, and parahippocampal gyrus) following 20 min of mini-bike exercise[17]. Moreover, a 10-minutes mild cycling exercise was found to enhance memory performance by increasing coupling between the hippocampus and cortical regions[12]. While evidence for the role of wake spindles in memory processing is limited, it

has been reported that the neural sink and source of wake spindles are similar to those of sleep spindles[18]. Additionally, visual inspection of recordings from wired electrodes in rats indicated that wake spindles are associated with ripple activity, much like sleep spindles[18].

Reviewer #2 (Remarks to the Author):

This study examined the association between working memory (WM) performance and physical exercise (PE) as well as spindle-ripple coupling measures. The authors reported that the WM performance was greater in the PE session compared to the rest session and also found a positive correlation between the performance and ripple rate. They further claimed that the spindle-ripple coupling was stronger in the PE session. The topic is interesting, but there are several major issues with the results presented in this study.

Major points

1) Spindles are spontaneously generated in the thalamocortical network during NREM sleep and the transmission of sensory input is also suppressed during sleep spindles. Unlike hippocampal sharp wave ripples and cortical up and down states, there is no compelling evidence for the existence of spindles during awake state.

Reply: We agree that spindles are primarily observed during non-REM sleep. However, spindle-like activity has also been reported during wakefulness[18], with similarities in brain regions, including layers IV, V-VI, and II-III, and associations with ripple activity. To compare wake and sleep spindles, we conducted a study with simultaneous EEG-MEG recordings in five participants during a memory task (see procedure below). After four hours of sleep, participants stayed awake from 2 a.m. to 9 a.m., at which point the MEG-EEG measurements began. The experiment started with a memory task, beginning with an encoding phase. During this phase, participants were presented with 30 sequences, each containing either 2, 3, or 4 pictures of everyday objects (e.g., a coffee cup, a cat, a screwdriver), totaling 90 pictures. Each picture was displayed for 500 ms, with a 100 ms interval between pictures and a 3-second interval between sequences. After the encoding phase, participants completed the first retrieval phase. They were shown sequences that were either old (from the encoding phase), new (entirely novel), or mixed (a combination of old and new pictures). For each condition (2, 3, 4 pictures), they saw five sequences of each type (old, new, mixed), resulting in 15 sequences per condition and 135 pictures in total. After viewing a sequence, participants identified it as old, new, or mixed using a button press. Following the first retrieval phase, participants were asked to sleep for 30 min during MEG-EEG measurement. Afterwards, they completed a second retrieval phase identical to the first, with the same structure and number of sequences per condition.

[image redacted]

We detected spindle events during the 30 min sleep across all channels within the spindle frequency band (13–20 Hz), using the same method applied for wake spindles in our study. We compared their topography, duration, and averaged signal. The number of spindle events was visualized using topographic plots, which showed a higher event density in the fronto-central region for both sleep and wake spindles (see ***Figure R3 and R4***).

Subsequently, we analyzed spindle events specifically in the fronto-central region of interest (ROI), including channels Fz, Cz, FC1, and FC2. We compared the spindle durations and averaged signals within this ROI. Spindle duration was determined by applying an envelope to the spindle band-filtered data and identifying peaks that exceeded a threshold defined as the mean plus one standard deviation. The spindle start was marked as the trough before the first peak, and the end as the trough after the last peak.

Using this method, we generated a bar graph of spindle lengths, showing that sleep spindles typically lasted longer than 0.5 sec but less than 2.5 sec (mean sleep = 0.945 sec; mean awake = 0.751 sec). Spindle durations from both sleep and awake states, along with example single spindles, are presented in ***Figure R4, R5.***

However, in this manuscript, the authors focused on the spindle activity after the onset of the stimulus.

Reply: We agree that our approach lacked clarity. We focused specifically on spindle activity following stimulus onset, aiming to determine whether specific frequency bands, particularly spindles, were modulated by the stimulus. This analysis was intended to uncover changes in brain dynamics directly related to task processing.

We added this to the methods section. The new paragraph now reads:

Our study aimed to explore whether specific frequency bands, particularly spindle activity, were modulated by the stimulus. By analyzing spindle activity after stimulus onset, we sought to identify task-related changes in brain dynamics. Spindle activity is believed to create a temporal window for ripple events to occur[19]. In the EEG, spindle oscillations generated within thalamocortical loops are primarily measured at fronto-central electrodes[20].

It seems that what they reported as the spindle activity was only the increase in the beta power during awake state, not spindles (in other words, the increase in the beta frequency band doesn't necessarily indicate the existence of spindle events with the properties and mechanism similar to the sleep spindles).

Reply: We thank the reviewer for their observation and acknowledge that the beta band (13-30 Hz) does overlap with the spindle frequency range. However, beta and spindle activities differ not only in frequency but also in their spatial distribution. Low beta oscillations (~13-20 Hz) are primarily observed in posterior EEG regions[21] and have been shown to increase during working memory retention, whereas spindle activity is typically seen in fronto-central regions[20]. Moreover, the spindles detected in our study represent short burst activities. Therefore, an increase in beta power during wakefulness does not necessarily reflect spindle events with the same properties or mechanisms as sleep spindles.

[figure redacted]

The topographical maps corresponding to low beta (5R and 7R retention conditions, 5M and 7M memorize conditions)[21]

[figure redacted]

Spindle topography[20]

We added to the Methods section that we limited our analyses to the central region of interest (ROI) to avoid confounding beta and spindle activity. Below is the revised text:

Both spindle and beta activity fall within the same frequency range, with beta activity also linked to working memory performance. To distinguish between these two types of activity, we focused on the fronto-central ROI (Fz, Cz, Fc1, and Fc2), as beta activity typically shows more posterior modulation. These channels were also used for subsequent spindle and coupling analyses.

In line 190, the authors referred to reference 8 and stated that "The authors found spindle amplitude during WM task in alignment with our study demonstrating PE improved working memory performance and increased spindle activity". However, in this reference the relationship between the alpha/beta frequency range (6–20 Hz) during encoding and sleep spindles was investigated and the spindle activity during task was not examined.

Reply: The reviewer is correct, and we apologize for the incorrect description. Below is the revised text:

The impact of PA on sleep spindle activity is uncertain, with some studies suggesting that daily repeated exercise can enhance 13-16 Hz spindle power during sleep[22], while others did not find effects of activity on spindle activity[23]. We observed spindle activity in both PA and rest sessions, topographically overlapping with sleep spindle findings in previous studies[20]. Notably, the amplitude of sleep spindles increased after the working memory task facilitating memory consolidation. These findings suggest that PA improves working memory performance by enhancing spindle activity.

The authors showed raw traces during ripples but no exemplary detected spindle event was shown.

Reply: We agree with the reviewer that this figure is missing. Here, we added an example of a single spindle activity from this study.

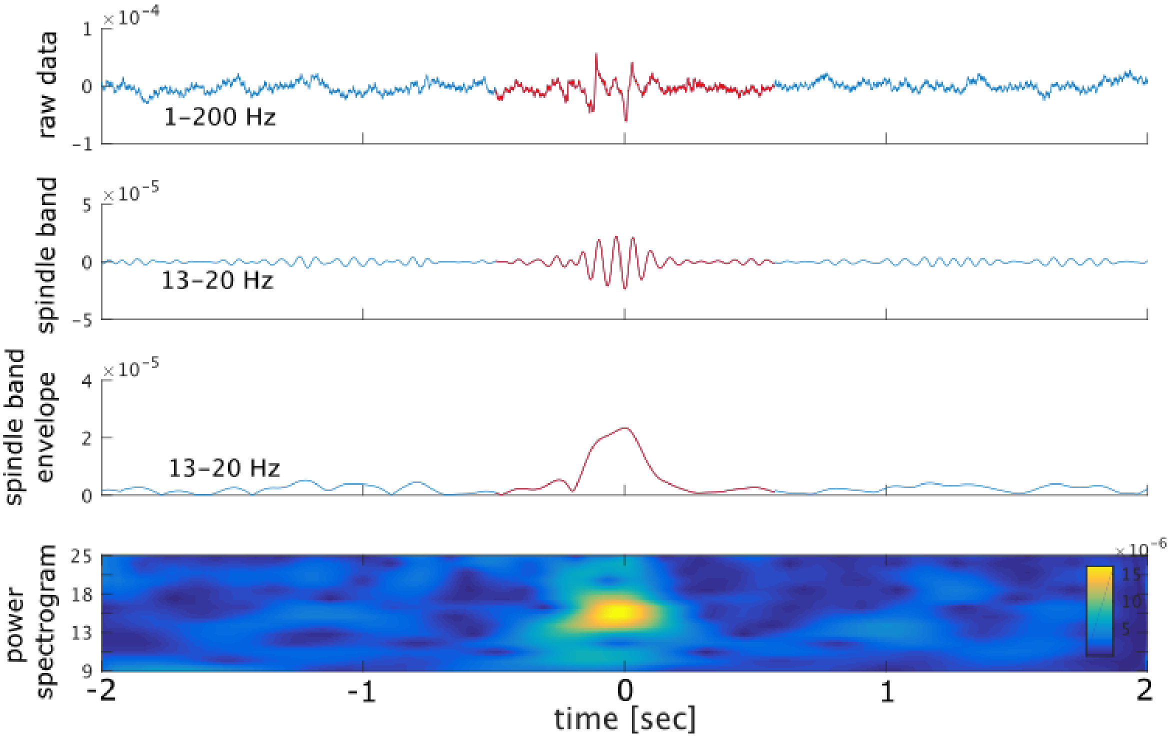

Fig. 6 | spindle activation in EEG.
An example of spindle detected in our analysis. The upper line shows the broad band signal between 1 and 200 Hz. The second time series shows the same signal in the spindle band. The third time series shows the Hilbert transform of the spindle band activity. The last graphic shows the time frequency representation of the signal filtered between 13-20 Hz.

Especially, in their spindle detection method, the duration, topographies and the frequency range of spindles they used/reported is not consistent with the ones widely considered for the sleep spindles:

Reply: Thank you for raising this point. In the original manuscript, we used the same detection approach for both ripples and spindles to ensure consistency between these neural markers. While this is not the standard algorithm for spindle detection, we aimed to demonstrate that our method reliably identifies sleep spindles and achieves high agreement between sleep and wake spindles. To investigate the overlap in region, duration, and frequency between sleep and wake spindles, we conducted a targeted sleep experiment with five participants. Participants slept in the MEG for this study. Although it was not a full sleep study this approach allowed us to evaluate key similarities between sleep and wake spindles effectively.

We analyzed sleep spindles using the same method described in the manuscript (see ***Figures R3***). Rather than recording overnight, we used partial sleep deprivation to align the circadian time point with the prior wake recording. Participants were instructed to go to bed at 10 p.m. and wake up at 2 a.m. the night before the experiment, ensuring at least 7 hours of sleep deprivation. The experiment began around 9 a.m., during which participants slept in the MEG for 30 min. EEG/MEG activity was recorded while participants lay comfortably.

The bar graphs (see ***Figure R3A and R4A***) indicate consistency across different brain states. Both studies show a high probability of spindles in a frontal ROI, as we previously described. Additionally, spindle lengths exhibit a consistent distribution.

Despite the high spatial, temporal, and frequency-specific overlap between sleep and wake spindles, we agree with the reviewer that these may involve different processes. Therefore, we have renamed "spindles" to "wake spindles" to highlight the distinction from sleep spindles.

For example, the result session ‘Temporal coordination of ripples with wake spindle events’ now reads: We examined whether ripples were temporally locked to wake spindle events by comparing the frequency of ripples outside and within wake spindle events (see ***Fig. 5C*** and Methods for a definition of wake spindle events). In the PA session, we observed more ripples within than outside wake spindle events (N_{in} = 0.45; N_{out} = 0.24; t_{20} = 3.25; $p < .01$), but only a trend in the rest session (N_{in} = 0.40 ; N_{out} = 0.27; t_{20} = 1.93; p = .068). In the final step, we investigated when the ripple likelihood reaches its maximum relative to the wake spindle peak event. We found a peak in the ripple likelihood starting from 102 msec after the spindle onset ($p < 0.0001$)(see ***Fig. 5D***).

i) The authors considered all the events with the amplitude in the spindle frequency range exceeding the 4 z-score threshold value as the spindle event regardless of the duration of the event. However, the spindle duration should be longer than 0.5 s and hence a large portion of the events that the authors detected as spindles were shorter than the minimum spindle duration. Moreover, events longer than 3 s should also be excluded.

Reply: The reviewer is correct that we did not sufficiently describe the analysis. While we used spindle events with peaks exceeding 4 standard deviations, the reviewer rightly notes that spindle length should also be considered. To address this, we analyzed the frequency distribution of spindle lengths and found that the events averaged over 500 ms in duration, with none exceeding 3 sec (mean sleep spindles = 0.945 sec; mean wake spindles = 0.751 sec; see ***Figures R3 and R4***). This confirms that our approach effectively captures spindles with an average length of over 500 ms. We have added a detailed explanation of this analysis to the Methods section.

First, we filtered the EEG signal in the spindle frequency (13-20 Hz) and obtained the analytic amplitude $A_{(t)}$. The resulting time series was z-scored, and peaks with $z>4$ were detected. Note, that the peak of $A_{(t)}$ is not necessarily the peak/trough of the spindle oscillation. Given that ripples are aligned with a specific phase (see ***Results***) of the spindle activity we centered spindle events to the troughs of spindle oscillation. Hence, we epoched the data (-2 to 2 sec) around each detected peak in $A_{(t)}$. We then detected the trough of the spindle oscillation closest to the peak of $A_{(t)}$. This time point was used as the center of the spindle event. To determine spindle duration, we first applied an envelope to the spindle band-filtered data. We then identified envelope peaks that exceeded a threshold set at the mean plus one standard

deviation of the spindle event. The start time of a spindle was defined as the valley preceding the first envelope peak, and the end time as the valley following the last peak.

We agree that the previous figure did not clearly show that the spindle events are longer than 500 ms. This is because ripples are generally coupled with the spindle phase, occurring at the descending flank of a wake spindle event (see ***Fig. 5B***). Using ripples as the starting point results in aligning only the descending flanks of the oscillation, which leads to a single oscillation and flat edges due to ripples being found at both the beginning and end of the spindle. To better demonstrate that spindle events are longer, we aligned the spindle envelope to ripple time points (see ***Figure R5***). This alignment shows that spindle peaks tend to occur around ripple peaks. However, since ripples are not restricted to the spindle peak and spindle peaks are not always symmetrically positioned, the resulting event may appear shorter than 500 ms in the figure. Nonetheless, individual events are indeed longer, with a mean duration of 945 ms (see above).

ii) While (fast) spindles mainly occur in centro-parietal areas (for example see doi: 10.1523/JNEUROSCI.2604-11.2011 and 10.1152/physrev.00042.2018), the authors reported that the spindle activity was maximum at fronto-central electrodes and stated that this topographic location was consistent with the ones reported in reference 8. However, in this reference, it has been clearly reported that the spindle activity was most pronounced in centroparietal areas.

Reply: In the reference suggested by the reviewer, there is a description of the location of sleep spindles in humans:

'There are also slow spindles during the N2 stage of human NREMS, but these are less dense, smaller, and spread across parietal and frontal sites. Fast spindles during N3 occur with comparable amplitudes but more moderate density at central sites.'

Therefore, the detection of spindle activity in the frontal-central area is in line with previously published studies. Additionally, the spindle event topography is similar to that described in reference 8[20]. In our sleep deprivation study, the spindle during sleep also showed a similar topography (n = 5).

[figure redacted]

Spindle from reference 8[20].

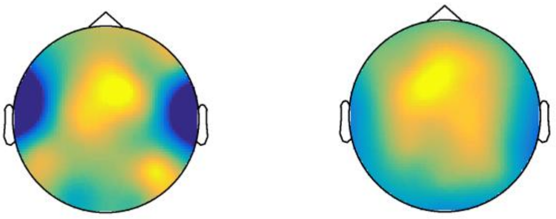

The spindle topography from this study and our sleep deprivation study.
iii) The Authors considered different frequency bands for spindles (14-18 Hz for figure 2, 14-16 Hz for figure 5B, 13-20 Hz for spindle detection, line 446) but none of them is consistent with the established spindle frequency range (10-16 Hz).

Reply: The reviewer is correct that the spindle frequency ranges we considered do not fully align with the traditionally established 10-16 Hz range. However, as the reviewer noted, differences between sleep and wakefulness could explain these variations. In our study, we used a data-driven approach to address these potential discrepancies. This approach revealed an increase in the 14-18 Hz range, which may reflect specific neural dynamics overlapping with the conventional spindle range. Additionally, 13-20

Hz has been shown to be effective for spindle detection, as observed in both this study and our sleep study. Given that wake spindles are less well-characterized than sleep spindles, we adopted this data-driven approach to capture the specific characteristics of this relatively new signal. As a result, there are differences from previous sleep studies. However, we observed significant overlap between wake and sleep spindles. To clarify this distinction, we have decided to use the term "wake spindles" throughout the article to differentiate them from sleep spindles.

In line 325, based on the time-frequency representation shown in figure 2A, the authors claimed that "The time-frequency representation confirmed clear spindle activity", however, the frequency range with increased power spans from 14 Hz up to 20 Hz which is higher than the spindle frequency range. On the other hand, there is a power reduction in a frequency range corresponding to the spindle frequency range (10-16 Hz) during a 1 s time window after the stimulus.

Reply: We appreciate the reviewer's concern. As mentioned previously, the spindle-like activity we detected during wakefulness may have different frequency characteristics compared to sleep spindles. Nonetheless, it exhibits burst-like dynamics and originates from the same brain region as sleep spindles. Additionally, we compared the frequency amplitude at the spindle peak and found that the main frequency range was similar between sleep and wake spindles (see ***Figure R7***).

2) Another main concern is that the spindle-ripple coupling analyses have not been conducted or reported appropriately as is explained below:

i) The authors do not directly compute the spindle-ripple coupling strength. There are commonly used phase amplitude coupling (PAC) methods that measure both the strength and phase of the coupling between spindles and ripples (for example see the PAC method used in doi:10.1038/nn.4119 which measures the strength and phase of coupling of the ripple amplitude to the spindle phase within spindle events).

Reply: We acknowledge that phase-amplitude coupling (PAC) is an effective method for analyzing spindle-ripple coupling strength, as it identifies spindle phases most strongly correlated with ripple amplitudes. Typically, phase-amplitude coupling measures the low-frequency phase at which the strongest high-frequency amplitude can be detected, based on ongoing activity. Here, we followed a slightly different approach: we preselected ripple events with the highest amplitudes and determined the spindle phase at which these events occurred (see ***Fig. 5B***). While ripple events were used to represent the coupling effect in ***Fig. 5B***, this visualization reflects the coupling between spindle phases and high-amplitude ripple events, considering only these significant events. We then examined whether these events were concentrated at both a specific frequency and phase. This analysis revealed that ripples are coupled to spindle activity. This approach, illustrated in ***Figures R5 and R6***, highlights the coupling effect between spindle phases and ripple amplitudes.

In spite of this, it is not clear for me that why the authors reported "low frequency" amplitude (1-25 Hz) around the ripple event and not the power in the spindle frequency range (Figure 5A).ii) It is more accurate to conduct the analyses of figure 5B only during spindles, since the phase values at the times with low amplitude in the spindle frequency range are meaningless (see for example the method used for phase-locking analyses in doi: 10.1111/ejn.14906).

Reply: We thank the reviewer for highlighting this point, allowing us to clarify our approach. While modulation of spindle activity was observed (***Figure R4***), filtering solely within the spindle frequency band could exclude relevant frequencies. Ripple coupling may occur with lower frequencies, where spindle frequencies act as harmonic multiples, reflecting harmonic oscillations rather than direct spindle-ripple coupling. Our analysis, which includes a broader frequency range, highlights the robustness of spindle activity despite higher amplitudes at lower frequencies. Notably, oscillations in the spindle band emerge as significant. Moreover, ripples predominantly occur near spindles, with significantly fewer outside these events, leading to stronger phase relations during spindles. Restricting the analysis to ripples during spindle events would limit the dataset and reduce generalizability. By including all ripples, we provide a more comprehensive view of information processing within brain networks. Our findings

show that typical oscillations occur around ripples, supporting a characteristic spindle trajectory (***Figure R5***), distinct from random time points. We have clarified this approach in the revised methods.

We also shown an envelope spindle frequency figure (see ***Figure R5***) and an averaged ripple likelihood around spindle peak (see ***Figure R6***).

iii) In another analyses (Figure 5C), the authors aimed to compare the number of ripples co-occurring with spindles with the isolated ripples. However, the time window they used to define these two different types of ripples is too short (-0.1 to +0.1 s for inside spindles and -0.1 o -0.3 for outside spindles) and is fixed for different spindles with different lengths. Hence their method does not correctly separate the ripples coupled to the spindles and the uncoupled ones. Since the spindles are events longer than 0.5 s, the time window to define the co-occurrence of the ripples with spindles should be longer than 0.5 s. In addition, as the length of the spindles is highly variable (from 0.5 to 3 s), it is more accurate to use different time windows for different spindles by defining the coupled ripples as the ones that occur between the onset and offset of spindles (for example see the method used in doi:10.7554/eLife.48881).

Reply: The reviewer is correct. We should take spindle duration into consideration. We have now adjusted the ‘spindle in’ interval based on the spindle duration, and the "spindle-out" interval is defined as 1 sec before and after each spindle event. With this new analysis we confirm the pattern reported in the previous version of the manuscript. The results are presented as density plots. Here is the revised text:

Result:

We examined whether ripples were temporally locked to wake spindle events by comparing the frequency of ripples outside and within wake spindle events (see ***Fig. 5C*** and Methods for a definition of wake spindle events). In the PA session, we observed more ripples within than outside wake spindle events ($N_{in} = 0.45$; $N_{out} = 0.24$; $t_{20} = 3.25$; $p < .01$), but only a trend in the rest session ($N_{in} = 0.40$; $N_{out} = 0.27$; $t_{20} = 1.93$; $p = .068$).

Method:

We then tested when ripples occurred relative to individual spindle events. Here, we defined the ‘spindle-out’ interval, which occurs outside spindle events, as the 1-second periods before and after each spindle event. The ‘spindle-in’ interval was defined based on the duration of the spindle event itself. Ripple density was calculated by summing ripple occurrences across four sensors positioned bilaterally covering the MTL region, averaging them across spindle events, and then dividing by the number of sample points. For each interval, we computed the average ripple count across all spindle events for each subject (see ***Fig. 5C***). This results in a ripple event rate both for the within and outside interval for each subject. This was done separately for the PA and rest session. The resulting ripple event rates were then compared using a t-test in each session.

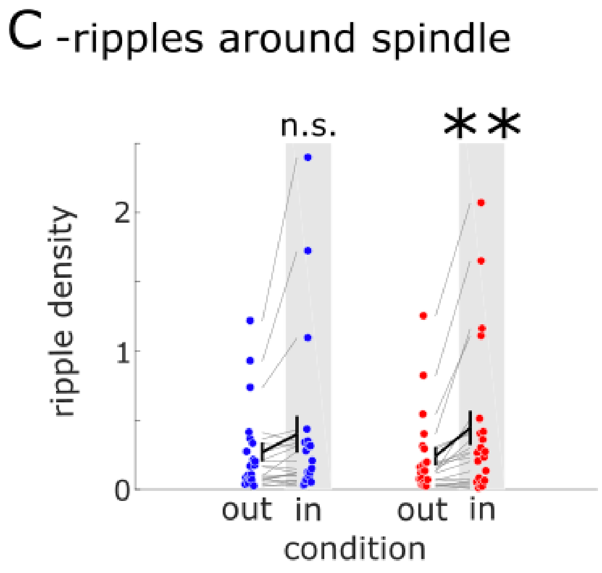

C: The ripple density during the 'in-spindle' and 'out-of-spindle' phases is plotted separately for the PA and rest sessions. Red denotes PA session data, while blue represents rest session data. (**: P<0.01, n.s.: not statistically significant)

iv) The authors claimed that "A peak of ripple likelihood before the respective spindle peak event might indicate that ripples trigger spindles" (line 462) and hence computed the ripple likelihood locked to the spindle peak. However, to investigate whether the ripples can trigger the spindle events, the ripple likelihood should be computed locked to the spindle onset and not its peak.

Reply: The reviewer is correct. We reanalyzed the results by aligning them to the onset of spindle activity and found that the highest likelihood of ripples occurred after the spindle onset. Therefore, we revised the sentence to state that the coupling between spindles and ripples is strong. The revised text reads:

Result:

In the final step, we investigated when the ripple likelihood reaches its maximum relative to the wake spindle peak event. We found a peak in the ripple likelihood starting from 102 msec after the spindle onset ($p < 0.0001$)(see ***Fig. 5D***).

Method:

Finally, we tested when ripple likelihood peaks relative to spindle peak events (see ***Fig. 5D***). A peak of ripple likelihood is shown shortly after the spindle onset. First, we summed ripple epochs across all spindle event peak for each participant, creating a time series of ripple likelihood around the spindle event peak for each participant. These individual time series were then averaged across subjects. This averaged ripple rate was compared against a surrogate distribution. To construct this distribution, we shifted the summed ripple epochs around spindle event onset for each participant over time and averaged the resulting time series across participants. This process was repeated 1000 times, resulting in 1000 surrogate ripple event series. We determined the likelihood of ripple events at each time point relative to the surrogate distribution at each time point by estimating the probability density functions (pdf.m in Matlab). This yielded a probability value p for each time point around the spindle event.

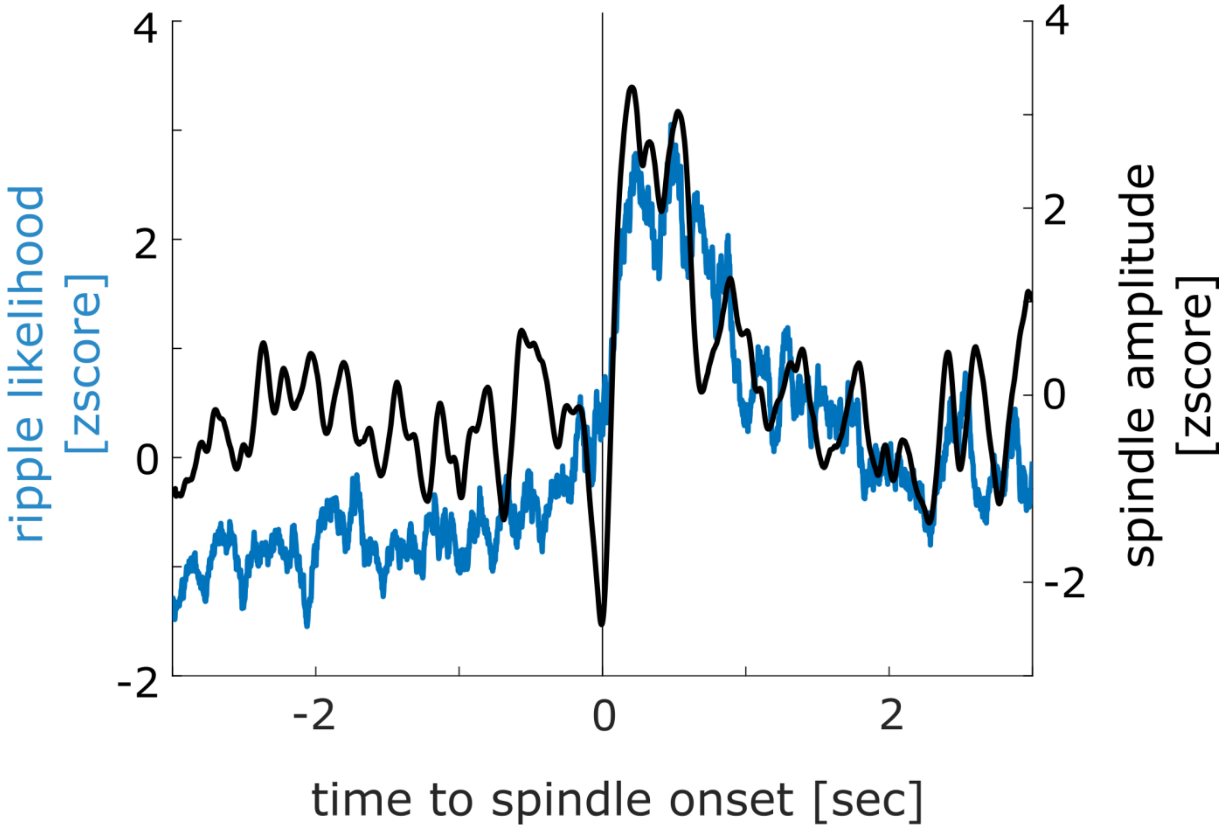

***D*: Ripple likelihood around the spindle onset. The time 0 represents the spindle onset.**

3) For time-frequency representation (TFR), why don't the authors use the more accurate and widely used TFR methods like wavelet or multitaper (mtmconvol function of the FieldTrip toolbox, see for example: doi:10.1038/nn.4119)? The robustness of results to a different TFR method should be checked.

Reply: We are aware that different time-frequency methods exist and that each method has its individual characteristics. The Hilbert transform is a reliable method in science and applied in various fields where oscillations need to be investigated, including analyzing EEG and MEG signals[24–26]. The advantage of the Hilbert transform is that it accurately calculates the envelope of a given signal and is most flexible in the definition of the bandwidth as compared to multitaper and wavelet approaches. Further, the Hilbert transform extracts instantaneous amplitude and phase information[27,28], whereas the result of multitaper and wavelet analysis depends on an analysis window. The wavelet and multitaper analysis might be more accurate for specific applications but we think that Hilbert transform is suitable for our approach. To compare the suitability to detect frequency bursts and to check the robustness of our results, as suggested, we performed two control analyses:

I) To quantitatively compare the Hilbert transform as used in our study with the continuous Wavelet transform using the Morse wavelet, we generated simulated data by embedding a 120 Hz burst of 18ms ms width (FWHM of a gaussian window) into a noisy signal. The ***Figure R8 A*** shows the raw signal, the bandpass-filtered signal (105-130Hz), the analytical amplitude of the Hilbert transform of the bandpass-filtered signal and the result of the continuous wavelet transform using a wavelet of center frequency 117.4 Hz. The shape of both time-resolved frequency decompositions are similar (correlation value of r=0.96).The ***Figure R8 B*** shows the time-frequency representation of the continuous wavelet transform and the ***Figure R8 C*** that of the Hilbert transform. Both methods generate similar localizations of the simulated bursts, although different in temporal and spectral spread (which could be controlled using the Hilbert transform). From this comparison it is not obvious that one is more accurate than the other method.

II) In a control analysis, we computed the ripple time-frequency representation with the continuous wavelet transform using the Morse wavelet. Both wavelet transform and Hilbert transform show a clear ripple amplitude around 80-150 Hz (see ***Figure R8 D, E***).

4) To find the association between ripple density and different N-back conditions, the authors conducted the correlation analyses between only 3 data points and separately for the rest and PE sessions which is not accurate. Instead, the comparison analyses like ANOVA should be conducted.

Reply: We appreciate the reviewer's concern. Instead of using an ANOVA, we employed linear regression in our analysis to examine the three conditions, as it offers several methodological advantages in this context. Here it was important to model the continuous relationship between the conditions and the dependent variable, which can be better captured by linear regression. Another advantage of linear regression is that it allows us to investigate a clear and interpretable relationship between the conditions and the dependent variable without strictly adhering to the assumptions of variance analysis. By using linear regression, we were able to model potential interactions or trends between the conditions and estimate the influence of each condition on the dependent variable in a continuous framework.

We added this information to our methods:

We correlated the ripple frequency in different N-back conditions with N-back (2-3-4, see ***Fig. 4C,D***), to estimate the linear association between ripple frequency and increasing task demands. We employed linear regression in our analysis to examine the three conditions, as it offers several methodological advantages in this context. Here it was important to model the continuous relationship between the conditions and the dependent variable, which can be better captured by linear regression. Another advantage of linear regression is that it allows us to investigate an interpretable relationship between the conditions and the dependent variable without strictly adhering to the assumptions of variance analysis. By using linear regression, we were able to model potential interactions or trends between the conditions and estimate the influence of each condition on the dependent variable in a continuous framework.

5) For the clusters shown in Figure 2B and Figure 4B, correction for multiple comparisons should be applied using the cluster-based permutation procedure (for example see doi:10.1038/nn.4119 or 10.1016/j.neuron.2017.11.020).

Reply: We appreciate the reviewer's suggestion. Here, we compared the averaged activity over the 1-2 sec window in ***Fig. 2B*** and the 0-1 and 1.5-2.5 sec windows in ***Fig. 4*B**. Since the activity was averaged over a time window, only a single statistical test was performed, and no multiple comparisons were involved. Therefore, correction for multiple comparisons is not necessary. We added this information to our methods:

The resulting spindle activity was averaged across all trials separately for rest and PA, then we z-scored the spindle activity with a baseline correction period from -200 msec to stimulus onset (see ***Fig. 2B***). To investigate the disparity in spindle activity between the rest and PA sessions over different time courses, we conducted a t-test to analyze the difference between the two datasets, using the averaged spindle activity between 1 to 2 sec.

Minor points

1) While the authors reported the correlation between ripple activity and performance, the correlation between spindles/spindle-ripple coupling and performance has not been reported.

Reply: We acknowledge that while we primarily focused on the correlation between ripple activity and performance in this study, the correlation between spindle activity, spindle-ripple coupling, and performance is also critical for understanding the relationship between neural oscillations and cognitive outcomes. However, we did not observe any statistically significant correlation between these measures and performance. We believe this could be due to the limited sample size in our study, which may have reduced the power to detect such relationships.

Additionally, it is possible that spindle activity and spindle-ripple coupling represent only part of the mechanisms underlying performance improvement. Their effects may need to be considered in conjunction with other neural processes to fully account for the observed behavioral outcomes. Nevertheless, we did observe higher spindle amplitudes and stronger spindle-ripple coupling after physical activity. Combined with findings from other studies, these phenomena provide insights into how physical activity might influence working memory processes.

2) Is there any difference in the ripple/spindle activity after target versus standard stimulus?

Reply: We appreciate the reviewer's suggestion. We found no differences in spindle amplitude or ripple activity between target and standard stimuli during the 1–2 sec window, nor in ripple activity after the target stimulus compared to standard trials, highlighting a general effect of both in working memory. The n-back paradigm is characterized by a rapid succession of stimuli, which may limit its sensitivity to specific modulation effects on targets and standards.

3) It is not clear for me why the correlation coefficients have been transformed to inverse hyperbolic tangent.

Reply: We appreciate the opportunity to provide clarification. The inverse hyperbolic tangent function (also known as Fisher's z-transform) is essential for normalizing correlation coefficients, which range from -1 to 1 and generally are not normally distributed. This transformation stabilizes the variance of the correlation coefficients, allowing for more accurate hypothesis testing and confidence interval estimation. By approximating a normal distribution, the z-transform facilitates statistical inference and comparison across different studies or groups. Additionally, it helps mitigate the influence of extreme correlation values, ensuring more robust analyses. Overall, applying Fisher's z-transform enables drawing more reliable conclusions.

4) Line 157: the unit of phase is not reported.

Reply: We apologize for the missing information. The unit of phase is radians, as shown in ***Fig. 5B***. Here we added it to the result.

We found that ripples were locked to different phases φ depending on the session ($\varphi_{PA} = 0.24$ rad; $\varphi_{rest} = 1.49$ rad; $F = 7.05$; $p = 0.011$).

5) Line 316: "we band-pass filtered the MEG signal at 21 exponentially". MEG or EEG?

Reply: We apologize for this typographical error. It should be EEG at this part. Here is the revised text:

To calculate the time-frequency representation, we band-pass filtered the EEG signal at 21 exponentially increasing center frequencies (ranging from 1 to 40 Hz), each with a bandwidth of 10% around the center frequency bands.

6) Overall, while the method in the manuscript text is clearly explained and well written, the required information for each figure has not been provided clearly in the figure or its legend. For example: In all the figures, the number of data points that have been used to compute the average values is not reported.

Reply: We apologize for the confusion. The figures present the average values along with the standard error, indicated by the black line between the two conditions. For example, in ***Fig. 1B and 1C***, the endpoint represents the mean value, while the vertical line indicates the standard error. Additionally, we have included the number of data points in the figure captions. Below are the revised figure captions:

Fig. 1 | Behavioral Performance.

A: Task design. Subjects were instructed to rest or use a pedal trainer before the N-back task.

B: (left): shows that hit rate increased across all conditions. (right): Hit rate across different N-back conditions. The black line between the two conditions represents the mean value and standard error, while the endpoint represents the mean value, and the vertical line represents the standard error. (n=21; +: P<0.05, *: P<0.017, **: P<0.003, Bonferroni-corrected)

C: (left): Individual false alarm rate. (right): False alarms across different N-back conditions. Red indicates PA and blue rest session. (n=21; +: P<0.05, n.s.: not statistically significant)

Fig. 4 | Ripple modulation

A (upper): Topographical map of ripple likelihood across MEG sensors for rest (left) and PA (right). (lower): Correlation coefficients of ripple distributions between pairs of subjects. Higher correlations coefficients following PA indicate a more consistent spatial structure of ripples likelihood across subjects. (n=210; ****: P<0.0001)

B (left): Time series of ripple likelihood. (middle): linear regression between individual memory capacity and ripple likelihood in segmented intervals. (right): The ripple likelihood time series from grand-averaged and different sessions, along with the corresponding t-values. (n=21; *: P<0.05, n.s.: not statistically significant)

C: Ripple count across N-back conditions displayed individually.

D: Correlation coefficients for rest (blue) and PA (red) calculated between the ripple likelihood and the 2, 3, 4-back levels. (n=21; *: P<0.05)

Fig. 5 | MEG-Ripple and EEG low-frequency coupling results.

A (left): Grand-averaged slow oscillations around the ripple peak compared to the shuffled peak. (right): The slow oscillations around the ripple peak from different sessions. The pink color represents the grand-averaged slow oscillations, the blue color represents the rest session, and the red color represents the PA session.

B (left): Phase distribution of low frequency around the ripple peak. (right upper): The phase distribution from 14-16 Hz from different sessions and the dominant phase from individual subjects. B (right lower): Average dominant phases across sessions. (n=21; *: P<0.05)

C: The ripple density during the 'in-spindle' and 'out-of-spindle' phases is plotted separately for the PA and rest sessions. Red denotes PA session data, while blue represents rest session data. (n=21; **: P<0.01, n.s.: not statistically significant)

D: Ripple likelihood around the spindle onset. The time 0 represents the spindle onset.

Figure 2A: what measure is shown in the left panel?

Reply: We apologize for the confusion. The left panel in ***Fig. 2A*** is only intended to show the channels used in our analysis; it is not a topographical representation of any analysis.

Figure 2: the definition of the zero time should be stated in the figure legend.

Reply: We appreciate the suggestion. Here is the revised text:

Shaded areas represent the standard error across subjects. The gray shading indicates the interval displaying significant differences between rest and PA. Time 0 represents stimulus onset.

Figure 3A: what do the two blue and red colors represent? There is no legend.

Reply: We apologize for the confusion. The red color represents the probability of ripple intervals during the PA session, and the blue represents the probability of ripple intervals during the rest session. We have added the definition to the figure legend. Here is the revised text:

A (left): The grand-averaged signal of ripple activity. (middle): Time-frequency plot of ripple activity. (left): The probability of the interval time between two ripples across subjects. The error bars represent

the standard deviation. The blue bar represents the ripple interval during the rest session, and the red bar represents the ripple interval during the PA session.

Figure 3B: the time scale has not been shown.

Reply: We apologize for the confusion. The time period is 4 sec, we added the time scale into the figure.

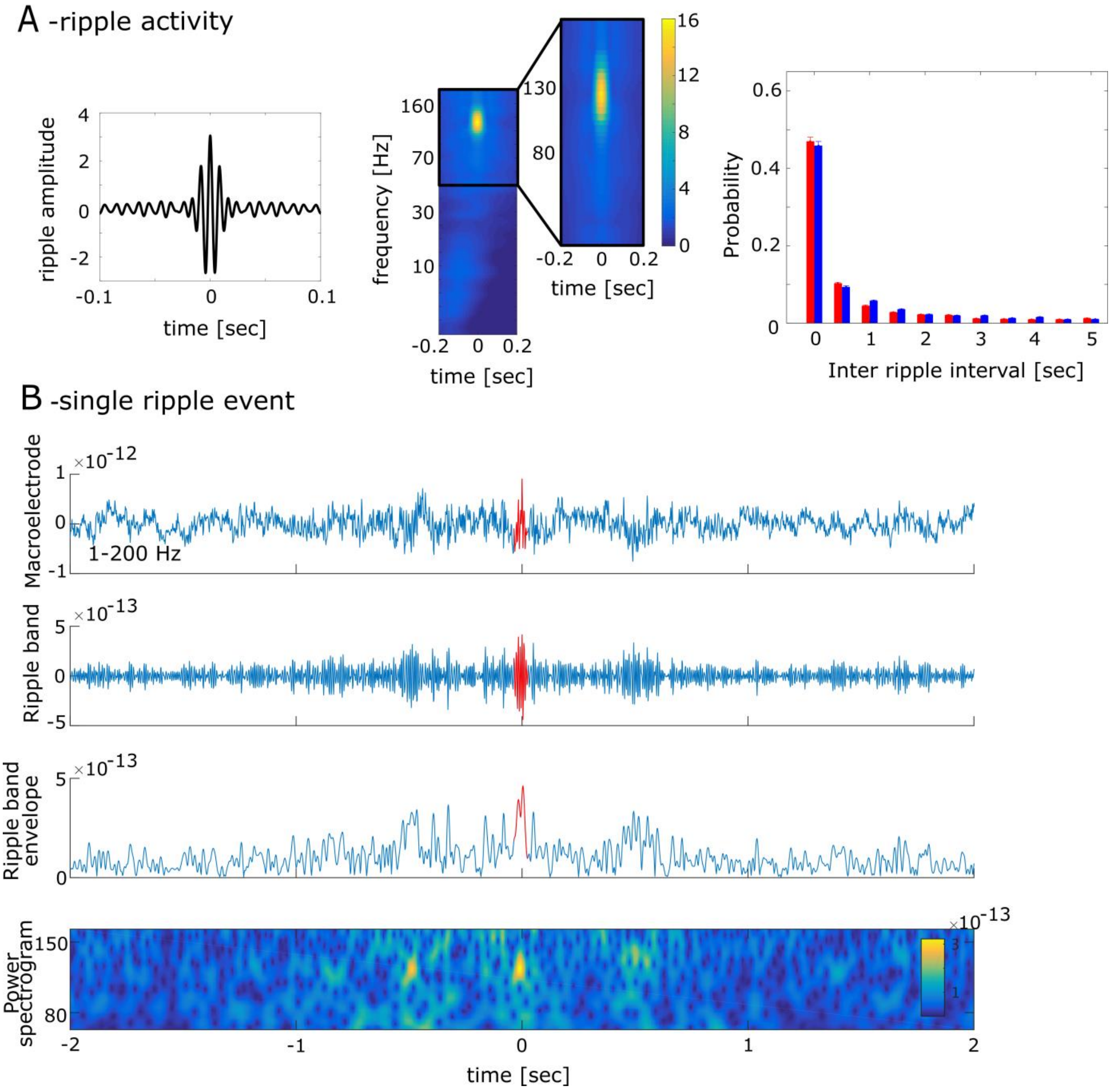

Figure 4A top: no color bar is shown.

Reply: Here is the revised figure.

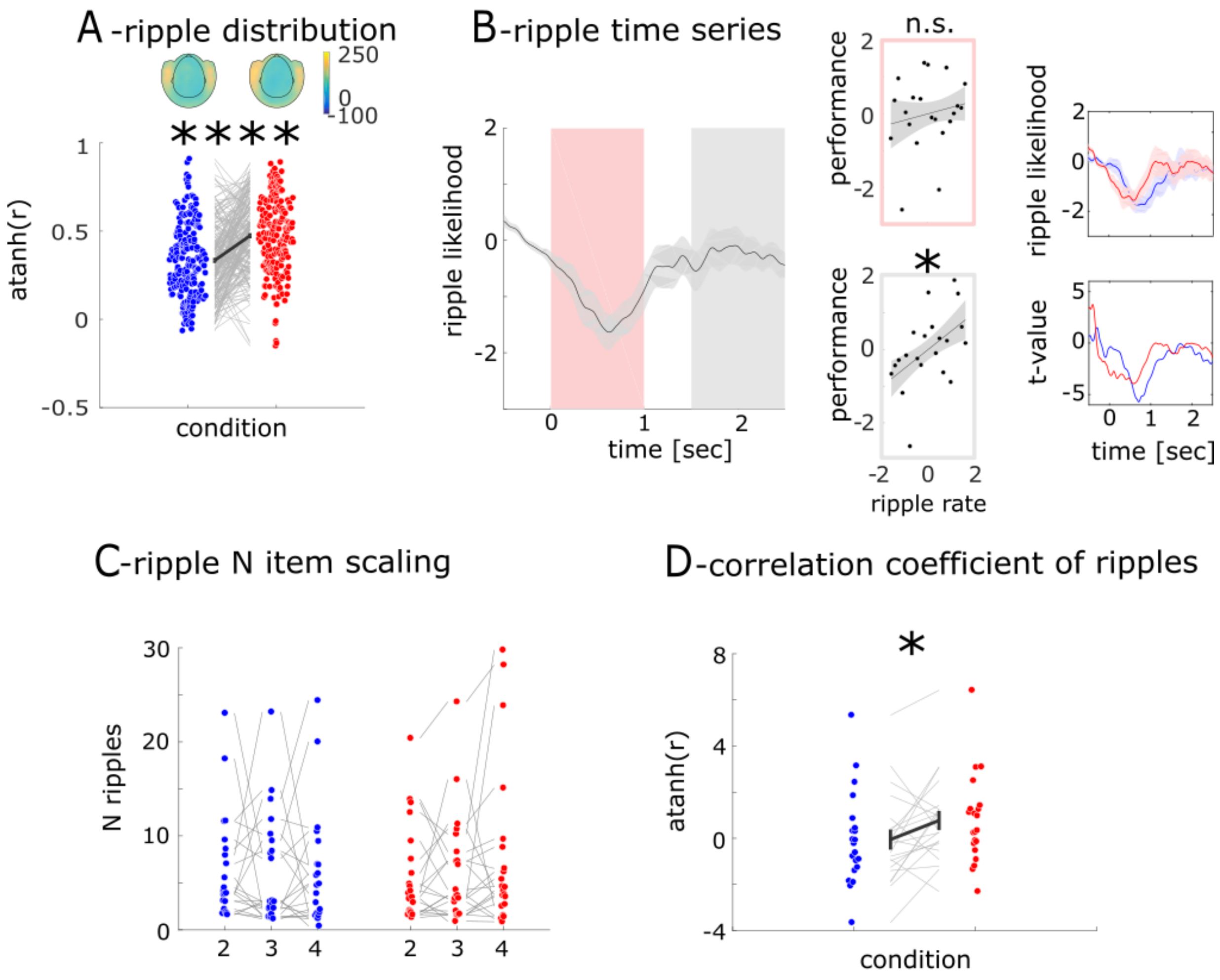

Figure 4C: it is better to report ripple density than numbers.

Reply: We changed the figure accordingly.

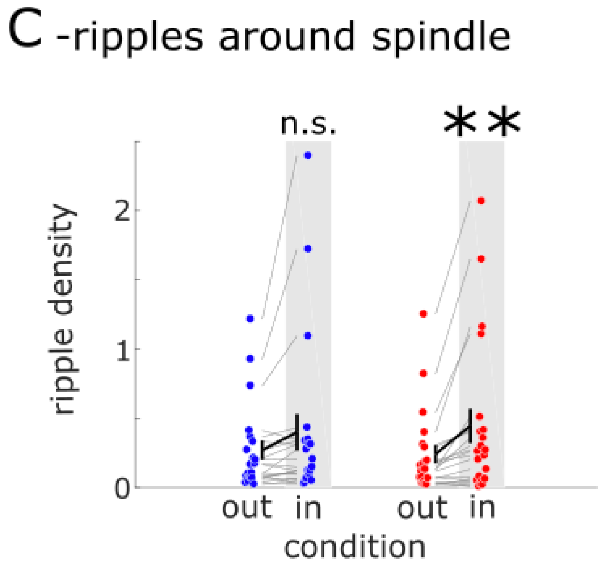

C: The ripple density during the 'in-spindle' and 'out-of-spindle' phases is plotted separately for the PA and rest sessions. Red denotes PA session data, while blue represents rest session data. (**: P<0.01, n.s.: not statistically significant)

Figure 4 and 5: the legend explaining the blue and red colors is not shown.

Reply: We apologize for the missing information. The red color always represents the PA session, and the blue color represents the rest session. We have added this information to the figure caption.

Figure 5A: The frequency band of "low frequency amplitude" that is shown in the y axis is not mentioned in the figure. In the text, the frequency band is written to be 1-25 Hz which does not correspond to what is commonly considered as low frequency in the EEG signal. As was mentioned above, it is also not clear why the authors computed the amplitude in this frequency range instead of the frequency range of spindles.

Reply: We thank the reviewer for raising this point, as it allows us to clarify our approach. At the time of our analysis, we observed modulation of spindle activity (**Fig. 3**), but filtering exclusively within the spindle frequency band would have excluded other potential frequency bands. While such filtering could reveal coupling, it might not necessarily reflect a direct relationship. Additionally, there could be coupling between ripples and lower frequencies, where the spindle frequency acts as a harmonic multiple. In such cases, we would be investigating harmonic oscillations rather than direct spindle-ripple coupling. The fact that spindle activity is observed despite the broad frequency range emphasizes the robustness of our analysis. Although lower frequencies typically exhibit higher amplitudes, oscillations in the spindle band are the ones that emerge as significant. It is true that spindle phase relations with ripples outside these events are less informative, and our previous analysis shows significantly fewer ripples occur outside spindle events. This results in stronger phase relations during spindle events. Focusing on the entire signal could increase the sensitivity to weaker or less obvious coupling relationships that might be missed when limiting analysis to specific spindle events. Including all ripples in our analysis offers a more comprehensive understanding of the underlying mechanisms of information processing within brain networks. Furthermore, our data show that, on average, a typical oscillation is present around ripples, indicating a characteristic spindle trajectory across all ripples. In contrast, random time points do not produce the same results (see ***Fig. 4***), suggesting that ripples are more likely to occur in close temporal proximity to spindles. We appreciate your suggestion and have emphasized these points in our revised method:

Since there could be coupling between ripples and lower frequencies, where the spindle frequency acts as a harmonic multiple. We investigated the ripple centered low frequency band rather than only spindle frequency band. In such cases, we would be investigating harmonic oscillations rather than direct spindle-ripple coupling. The fact that spindle activity is observed despite the broad frequency range emphasizes the robustness of our analysis. Although lower frequencies typically exhibit higher amplitudes, oscillations in the spindle band are the ones that emerge as significant.

We also shown an envelope spindle frequency figure (see ***Figure R5***) and an averaged ripple likelihood around spindle peak (see ***Figure R6***) at the bottom of this document.

Figure 5D: What does the vertical red line represent?

We apologize for any confusion caused. The vertical red line originally represented the ripple peak. However, since we revised ***Fig. 5D***, the vertical black line now represents the spindle onset.

Reviewer #3 (Remarks to the Author):

The authors provided evidence regarding the effects of physical exercise (PE) on brain rhythms, specifically focusing on spindle and ripple activity as foundational elements of working memory (WM). Using electroencephalography (EEG) and electromyography (EMG) devices, the study first demonstrated that PE leads to significant improvements in WM performance, as assessed by the N-back task. Furthermore, the enhancement in WM was associated with increased ripple activity within the medial temporal lobe, particularly in coupling with the spindle. These findings contribute to our understanding of the functional role of ripple-spindle coupling in the context of PE-enhanced WM,

underscoring the neurophysiological impact of PE on cognitive function. Overall, the manuscript is well-written, and the results are presented well. The study contributes to the field by elucidating the relationships between PE, WM, and spindle-ripple coupling.

Reply: We thank the reviewer for this positive assessment.

However, the introduction would benefit from descriptions of current memory theories, the relationship to PE, and the relationship between spindle and/ or ripple activity during PE.

Reply: We would like to first note that we have changed the term "physical exercise" (PE) to "physical activity" (PA), as we agree with another reviewer's comment that PA more accurately describes the locomotion task used in our study. We appreciate the reviewer's suggestion and have added the following passage to the introduction:

Brief sessions of physical activity (PA) like walking[13,14] or cycling[12] enhance working memory performance[6–9,12] likely due to increased hippocampus-cortex coupling[12].

Further, the description of the PE used in the study lacks details regarding the methods used in the study. Specifically, there is ambiguity regarding the control of exercise intensity (which appears to be of moderate intensity).

Reply: The reviewer is correct that specific methods are needed to control physical exercise (PA), defined as planned, structured, and repetitive physical activity, which plays a key role in shaping human physiology and brain development[1]. Due to the unavailability of the speed monitor in our initial experiment, we lacked speed data. In response to this review, we invited a new group of participants to test how quickly they would pedal based on our original instructions. In these subsequent experiments, using the speed monitor, participants consistently maintained a pace of around 1 step per second across sessions. The correlation coefficient between speeds across four sessions (see ***Figure R2***) showed stable performance over time. As noted to avoid confusion, we have also changed the term "physical exercise" to "physical activity" in the manuscript.

For example, the abstract now reads:

Spindle-ripple coupling enhances memory consolidation during sleep. Ripples, representing the compressed reactivation of environmental information, provide a mechanism for retaining memory information in chronological order and are also crucial for working memory (WM) during wakefulness. Brief sessions of physical activity (PA) are proposed to boost WM. In concurrent EEG/MEG sessions, we investigated the role of PA in WM performance and high-frequency-ripple to wake spindle coupling. Ripples, identified in MEG sensors covering the medial temporal lobe (MTL) region, predicted individual WM performance. Ripples were locked to robust oscillatory patterns in the EEG defined spindle band. Wake spindle activity and ripples decrease during initial stimulus presentation and rebound after 1 sec. Behaviorally, PA enhanced WM performance. Neurophysiologically, PA scaled the ripple rate with the number of items to be kept in WM and strengthened the coupling between ripple events and spindle oscillations. These findings reveal that PA enhances WM by coordinating ripple-spindle interaction.

Additionally, since there was no physiological measure, for comparison with other studies, references or wheel parameters should be given. I assume the exercise was aerobic?

Reply: Yes, aerobic exercise is defined as sustained, low-intensity activity that relies on oxygen to fuel muscles over time, improving cardiovascular endurance. In contrast, resistance exercise uses external weights to progressively overload muscles, increasing strength and muscle size. Our physical activity, where participants moved at a moderate pace for 2 min, resembled aerobic exercise in intensity[2]. However, it lacked the sustained duration typical of aerobic exercise and did not involve external resistance, distinguishing it from resistance exercise[3]. We specifically chose this moderate activity to minimize MEG and EEG artifacts, such as ECG interference or changes in skin impedance caused by sweating[5], which can affect EEG electrodes. Similar moderate-intensity exercises, like wheel running in

rodents, have been shown to enhance hippocampal plasticity and improve memory, linking our findings to prior animal research[6].

We added this to the method section. The new paragraph now reads:

The pedal trainer was specifically designed in-house for MEG recording. All materials were carefully tested to ensure they contained no magnetic components. The device's angle and height can be adjusted to suit each participant's proportions, and its design allows for independent forward and backward movement of both feet. Participants were instructed to pedal at a moderate, self-adjusted pace, similar to a walking motion. While this activity resembles aerobic exercise—defined as movement sustained by oxygen delivered through the blood to active muscles—it did not have the prolonged duration typical of aerobic workouts[2]. This adjustment was made to minimize MEG artifacts, such as ECG interference or changes in skin impedance caused by sweating[5], which can affect EEG electrodes contact. Similar moderate-intensity exercises, such as wheel running in rodents, have been shown to enhance hippocampal plasticity and improve memory, linking our findings and previous animal research[6].

Clarifying these aspects is important for accurately interpreting the study's findings and implications.

Reply: We agree on this with the reviewer. We hope that we answered the questions

The following are my concerns regarding the study:

Comments:

Introduction:

Line 58: Please note the frequency range of spindles, like ripples.

Reply: We appreciate the opportunity to provide clarification. In rat studies, sleep spindles are typically observed in the 10–15 Hz range, while spindles during wakefulness occur at 7–9 Hz[18]. However, human studies reporting spindles during wakefulness are scarce. In our study, we detected spindle-like events during an awake experiment, characterized by burst-like activity resembling sleep spindles. This suggests the presence of awake spindles in humans, occurring around 13–20 Hz during offline awake periods. To compare wake and sleep spindles, we recorded simultaneous EEG-MEG activity in five individuals during sleep in a control experiment. Participants were awakened at 2 a.m. after four hours of sleep, and at 9 a.m., we conducted MEG measurements while they rested for 30 min. This setup allowed us to compare spindle dynamics between wake and sleep states (see ***Figures R3 and R4***). Using the same detection method for both states, we found that sleep spindles typically lasted around or over 0.5 sec, while awake spindles had shorter durations. To determine spindle duration, we applied an envelope to the spindle band-filtered data, identified peaks exceeding the mean plus one standard deviation of the spindle event, and defined spindle start and end times based on the valleys before and after the envelope peaks. We present the spindle durations from both sleep and awake experiments, with examples shown at ***Figure R3 and R4***.

Line 68: The phrase "PE enhanced detection" is unclear. Does this refer to an improvement in behavioral performance on the working memory task, or is it describing the increase in ripple activity mentioned in the following sentences?

Reply: We apologize for the confusion. We intended to convey that physical activity (PA) enhances behavioral performance on the working memory task. The sentence has been revised:

Physical activity (PA) enhances working memory performance. High-frequency ripples, key for organizing information into working memory, were detected in MEG sensors covering the medial temporal lobe (MTL) region. Both EEG-spindle and MEG-ripple rates decreased during stimulus presentation but increased after 1 sec.

Results:

Line 111: Typo? --"ripple frequency = .125Hz". Is it "ripple rate" meant?

Reply: We apologize for the confusion. This term refers to ripple density or ripple rate, which indicates how many ripples occur per second. We have revised the sentence:

Ripples in MEG resembled the time-frequency representation (mean duration = 31.91 msec, SD = 10.82 msec; ripple density = .125 Hz, SD = .098 Hz) observed in intracranial studies (see **Fig. 3B**)[29-31].

Methods:

Line 230 (Participants): Was there any subject dropout? Please discuss why a gender balance wasn't used.

Reply: We apologize for the oversight regarding gender balance and dropout information. Due to pandemic-related constraints, we prioritized participants who met all study requirements, which limited our ability to achieve perfect gender balance. As a result, there is a slight bias toward female participants (approximately 60% female, 40% male). We agree this is important and have clarified it in the text. Additionally, nine participants dropped out after the first session due to unwillingness to return, excessive movement during the memory task, or difficulty understanding the task.

In the context of a working memory study, a 60/40% gender bias is unlikely to be problematic for several reasons. First, research has generally shown that gender differences in overall working memory capacity are minimal, with men and women performing similarly on most standard working memory tasks. While small differences can exist in specific types of working memory tasks, such as spatial versus verbal memory, these differences are negligible in our study since we are not using tasks that specifically target spatial or verbal memory. Furthermore, statistical methods can account for any potential gender effects, ensuring that the modest imbalance does not bias the results. Additionally, if the study's focus is not on gender differences but rather on general working memory performance, this distribution remains representative enough for valid conclusions. Given the small gender effect sizes typically found in working memory research, the 60/40% split which should provide reliable and generalizable results.

Here is the revised text in method:

Twenty one participants (13 female, 8 male, mean age = 27.00 years, SD = 5.76 years) participate in the experiment. Nine participants of an initial pool of thirty subjects dropped out after the first session due to unwillingness to return, excessive movement during the memory task, or difficulty understanding the task. A 60/40% gender split was maintained, which is sufficient to provide reliable and generalizable results, as the effects of gender on working memory performance are typically small. All participants reported normal or corrected to normal vision and no history of neurological or psychiatric diseases. The recordings took place at the Department of Neurology, Otto-von-Guericke University Magdeburg. We invited participants to two separate MEG sessions with an average 75-day (SD = 65.27) interval between sessions to allow for a within-subject design.

Line 238 (Procedure): Please provide detailed information on the display parameters used for stimulus presentation, including the resolution and refresh rate of the display, the size/visual size of the stimulus, and any other relevant details regarding the visual presentation setup.

Reply: We thank the reviewer for highlighting this and apologize for the initial omission. Here are the detailed parameters used in the visual presentation setup, which have now been added to the manuscript at MEG recording session. The new paragraph now reads:

Stimuli were presented via rear projection onto a semi-transparent screen with an LCD projector (DLA-G150CLE, JVC, Yokohama, Japan) that was positioned outside the booth. The screen was placed at a viewing distance of 100 cm in front of the participants. The screen was placed at a viewing distance of 100 cm in front of the participants. The display screen resolution was set to 1920 x 1080. Each letter presented on the screen measured approximately 3 x 3.5 cm. The screen refresh rate was 120 Hz, corresponding to an 8.3 ms refresh interval.

Line 248: For the rest condition, please explicitly state that the subjects were sitting on the pedal trainer during the EEG/ MEG measurement.

Reply: We appreciate the opportunity to provide clarification. During the experiment, participants sat in an adjustable chair designed for MEG measurements. In the physical activity session, their feet were placed on a pedal trainer. However, during the rest session, the pedal trainer was removed from the MEG room to prevent participants from using it. Instead, a footrest of the same height as the pedal was installed. The participants' sitting posture remained the same throughout both sessions.

Line 291: Please define what is t=0 in your study. Is this reference point aligned with the onset of the stimuli?

Reply: The reviewer is right that t=0 marks the stimulus onset. We have added this information to the respective paragraph, and here is the revised text:

EEG data were re-referenced to the left mastoid and band-pass filtered between 1 to 40 Hz. To eliminate eye movement artifacts, we applied Independent Component Analysis (ICA), which was computed with the FastICA package for MATLAB (Version 2.5). Resulting MEG and EEG time series were used to characterize ripple band dynamics (see below) and low frequency activity, respectively, over the time course of visual target detection. Both MEG and EEG data were epoched into trials ranging from -2 to 3 sec relative to stimulus onset (sufficiently long to prevent edge effects).

(Recording): Please provide the topography of the EEG. Some electrodes are more prone to muscle artifacts. Thus, the electrodes depicted should be given.

Reply: The reviewer is correct. In this study, we exclude the trials with a variance exceeding 4 standard deviations both at PA and rest sessions to avoid artifact. Additionally, we didn't analyze the signal during the pedal trainer. Furthermore, the muscle artifact generally occurs in a higher frequency band around 20-300 Hz[32], while we only analysis low frequency band with EEG signal which around 13-20 Hz.

Line 308 (Behavioural performance): Please clarify the method used to address violations of the sphericity assumption in your ANOVA analysis.

Reply: We apologize for not providing sufficient statistical analysis. We compared the F value of PA ($F_{(1,120)} = 4.04$) and N-back condition ($F_{(2,120)} = 31.7$) obtained from the ANOVA on Hit results with the F values derived from the surrogate distribution (1000 repetitions). Our analysis revealed a significant difference between the real F value and the F values from the surrogate distribution in the N-back and PA/rest conditions ($P_{PA/rest} = 0.0042$; $P_{back} < 0.0001$). Here are the revised results and methods:

Result:

First, we evaluated target detection accuracy (hit rate; H; see ***Fig. 1B***) and false alarms (see ***Fig. 1C***). When we compared hit rate, we found a main effect of PA ($F_{(1,120)} = 4.04$; p = 0.0042) and N-back condition ($F_{(2,120)} = 31.7$; p < .00001) compared against a surrogate distribution (see Methods).

Method:

To address potential violations of sphericity and normality in the data, we compared the observed F-values to a surrogate distribution. This distribution was generated by randomly shuffling individual behavioral data across participants and conditions (motion and N-back) in 1000 iterations, ensuring that the resulting distribution preserved the same properties of sphericity and normality. For each iteration, we recalculated the F-values, resulting in a distribution of 1000 F-values. The probability (p-value) of the observed F-value was then determined based on its position within this surrogate distribution.

Line 327: The spindle frequency range is given as 14-18 Hz. Can you please provide a reference for looking at this range? In sleep, the typical (fast) spindle frequency range is 12-16 Hz.

Reply: The reviewer is correct that the spindle frequency ranges we analyzed do not fully align with the traditional 10–16 Hz range. However, as noted, differences between sleep and wakefulness could account for these variations. To address potential discrepancies, we used a data-driven approach that identified an increase in the 14–18 Hz range, possibly reflecting neural dynamics overlapping the

conventional spindle range. Additionally, the 13–20 Hz range has proven effective for spindle detection in this and our previous sleep study. Since wake spindles are less well-characterized than sleep spindles, we adopted this approach to better capture their unique features. While this may result in differences compared to prior sleep studies, we observed significant overlap between wake and sleep spindles. To clarify, we now use the term "wake spindles" throughout the article to distinguish them from sleep spindles.

For example, the abstract now reads:

Spindle-ripple coupling enhances memory consolidation during sleep. Ripples, representing the compressed reactivation of environmental information, provide a mechanism for retaining memory information in chronological order and are also crucial for working memory (WM) during wakefulness. Brief sessions of physical activity (PA) are proposed to boost WM. In concurrent EEG/MEG sessions, we investigated the role of PA in WM performance and high-frequency-ripple to wake spindle coupling. Ripples, identified in MEG sensors covering the medial temporal lobe (MTL) region, predicted individual WM performance. Ripples were locked to robust oscillatory patterns in the EEG defined spindle band. Wake spindle activity and ripples decrease during initial stimulus presentation and rebound after 1 sec. Behaviorally, PA enhanced WM performance. Neurophysiologically, PA scaled the ripple rate with the number of items to be kept in WM and strengthened the coupling between ripple events and spindle oscillations. These findings reveal that PA enhances WM by coordinating ripple-spindle interaction.

Aside from the frequency range, can you elaborate on any further criteria used for spindle detection?

Reply: The spindle activity is a burst-like sequence, it was reported lasting for at least 0.5 sec but no longer than 2-3 sec (see ***Figure R4***). In our coupling analysis, we defined spindle events: first, we filtered the EEG signal in the spindle frequency (13-20 Hz) and obtained the analytic amplitude $A_{(t)}$. The resulting time series was z-scored, and peaks with $z>4$ were detected. With this method, the spindle event we detected is around 0.5 sec.

Line 332: Running t-tests were used to compare average values at every 0.1-second interval following stimulus onset. Did the statistical results underly any testing for multiple comparisons, e.g., using a false discovery rate method? Give information on these results in the figures, e.g., in Fig. 2 B.

Reply: The reviewer is correct, and we apologize for the incorrect information in the Methods section. The tested difference is limited to the 1–2 sec interval, as accurately presented in the results section. Since we used the averaged spindle activity from 1 to 2 sec, resulting in only one t-test, there was no need for any correction for multiple comparisons.

Here is the revised text:

Results Section:

Spindle activity provides a temporal framework for ripple occurrence[19]. In the EEG, thalamic spindle activity is maximal at fronto-central electrodes[20]. We found a consistent temporal pattern across both sessions, with spindle activity decreasing immediately after stimulus onset, followed by a subsequent increase at fronto-central EEG electrodes (see ***Fig. 2A, left***), peaking at 1150 msec. Additionally, enhanced activity between 1 and 2 sec after stimulus presentation was observed in the PA conditions ($t_{20} = 2.20$; $p = 0.04$, see ***Fig. 2B***).

Methods Section:

To investigate the disparity in spindle activity between the rest and PA sessions over different time courses, we conducted a t-test to analyze the difference between the two datasets, using the averaged spindle activity between 1 to 2 sec.

Legends of the figures should be self-explanatory without the text. Fig. 1 B, C (right), as well as Fig 3B: Please add the meaning of the x-axis. Give the meaning of the asterisks and symbols in the legends of all figures. For instance, in Fig. 1 B, right a strange symbol is used.s

Reply: We apologize for the unclear definition. Here is the revised text and figures:

Figures

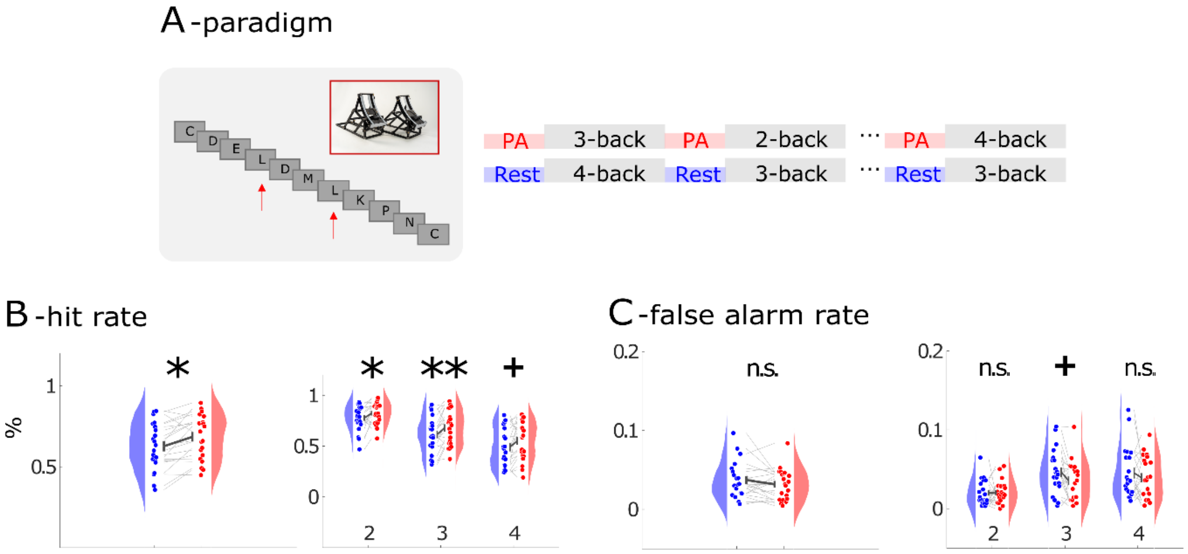

Fig. 1 | Behavioral Performance.

A: Task design. Subjects were instructed to rest or use a pedal trainer before the N-back task.

B: (left): shows that hit rate increased across all conditions. (right): Hit rate across different N-back conditions. The black line between the two conditions represents the mean value and standard error, while the endpoint represents the mean value, and the vertical line represents the standard error. (+: P<0.05, *: P<0.017, **: P<0.003, Bonferroni-corrected)

C: (left): Individual false alarm rate. (right): False alarms across different N-back conditions. Red indicates PA and blue rest session. (+: P<0.05, n.s.: not statistically significant)

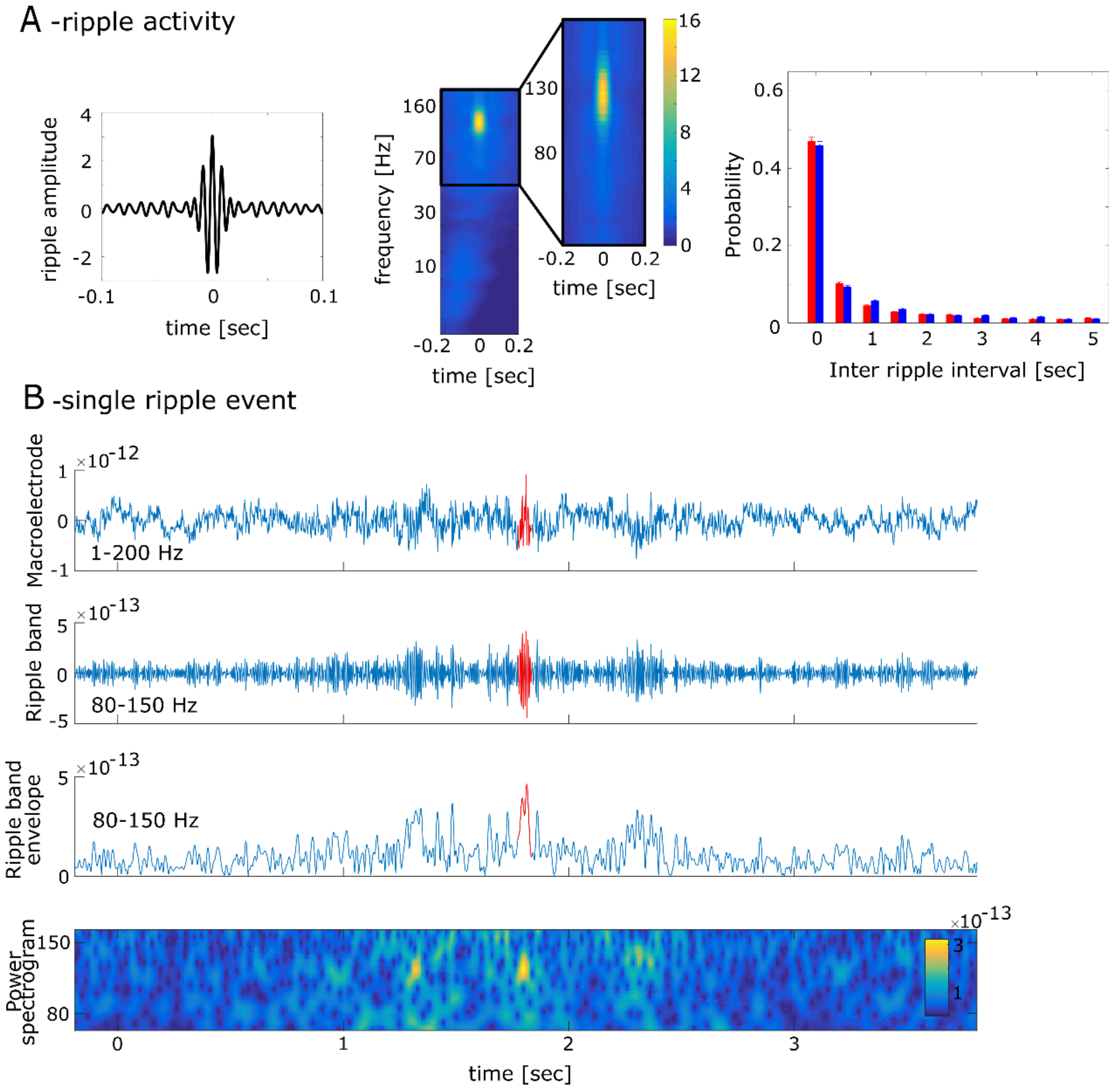

A -ripple activity
ripple amplitude
time [sec]
frequency [Hz]
time [sec]
time [sec]
Probability
Inter ripple interval [sec]
B -single ripple event
Macroelectrode
1-200 Hz
Ripple band
80-150 Hz
Ripple band envelope
80-150 Hz
Power spectrogram
time [sec]

Response figure：

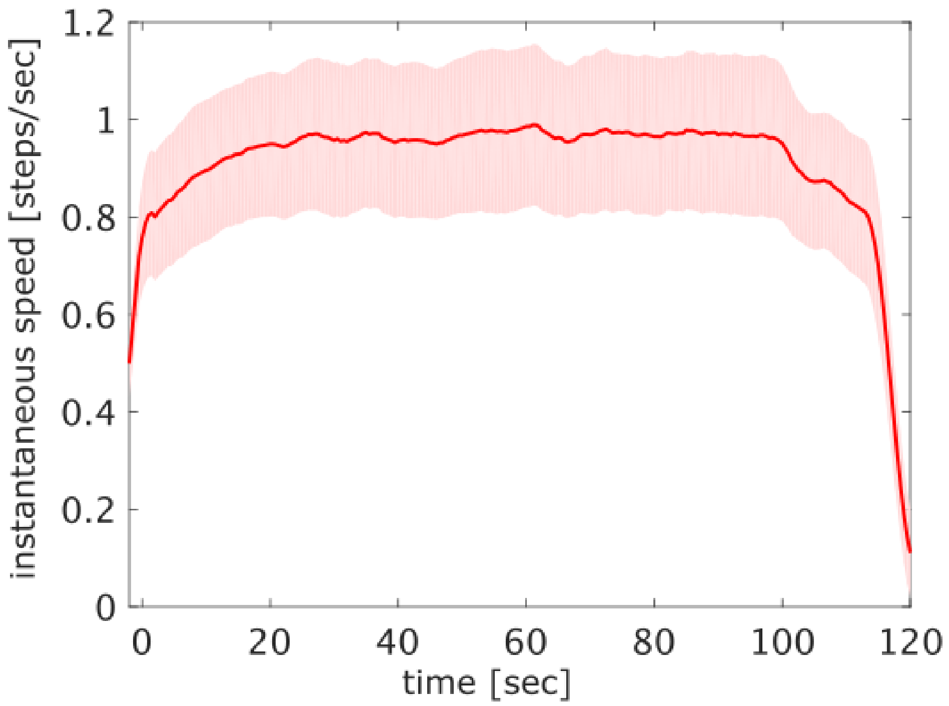

Figure R1 | The averaged speed during the PA sessions.

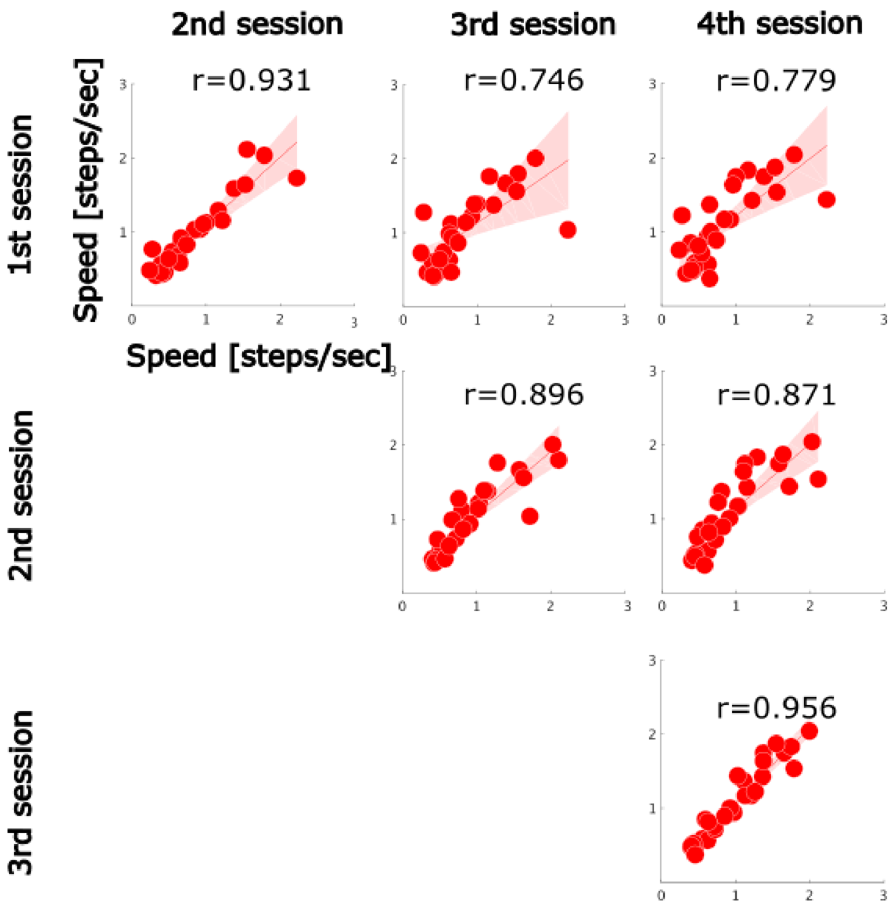

Figure R2| Correlation coefficients of pedal speed between four sessions. Red shaded areas show the 95 % confidence interval.

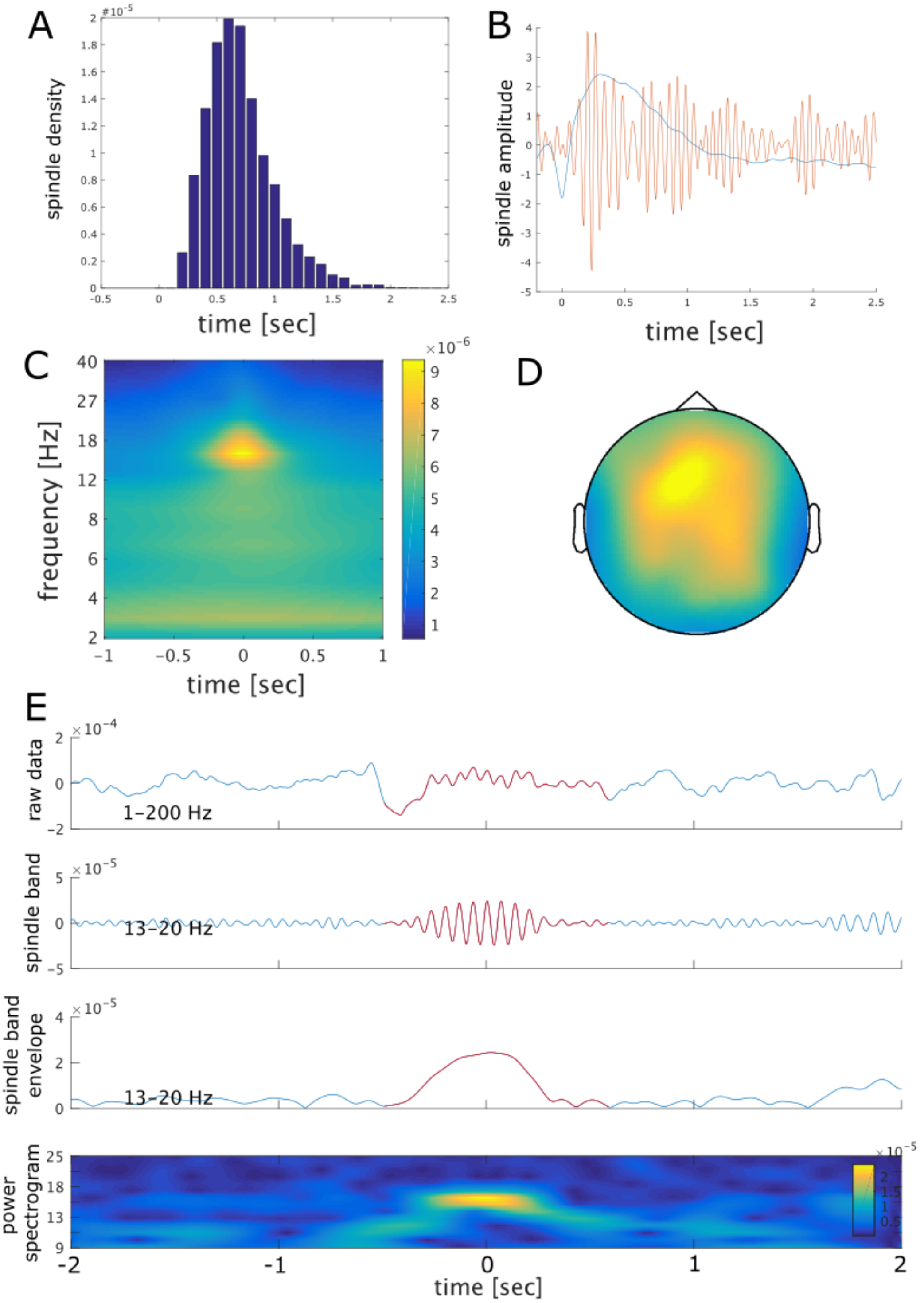

Figure R3 | Sleep spindle in EEG.

A: The sleep spindle distribution bar (spindles/minute). ***B***: Zscored averaged raw signal (orange line) according to the spindle onset and the Zscored averaged amplitude (blue line). ***C***: The averaged time

frequency of spindle according to the spindle peak. ***D***: The topography of spindle events. ***E***: An example of sleep spindle.

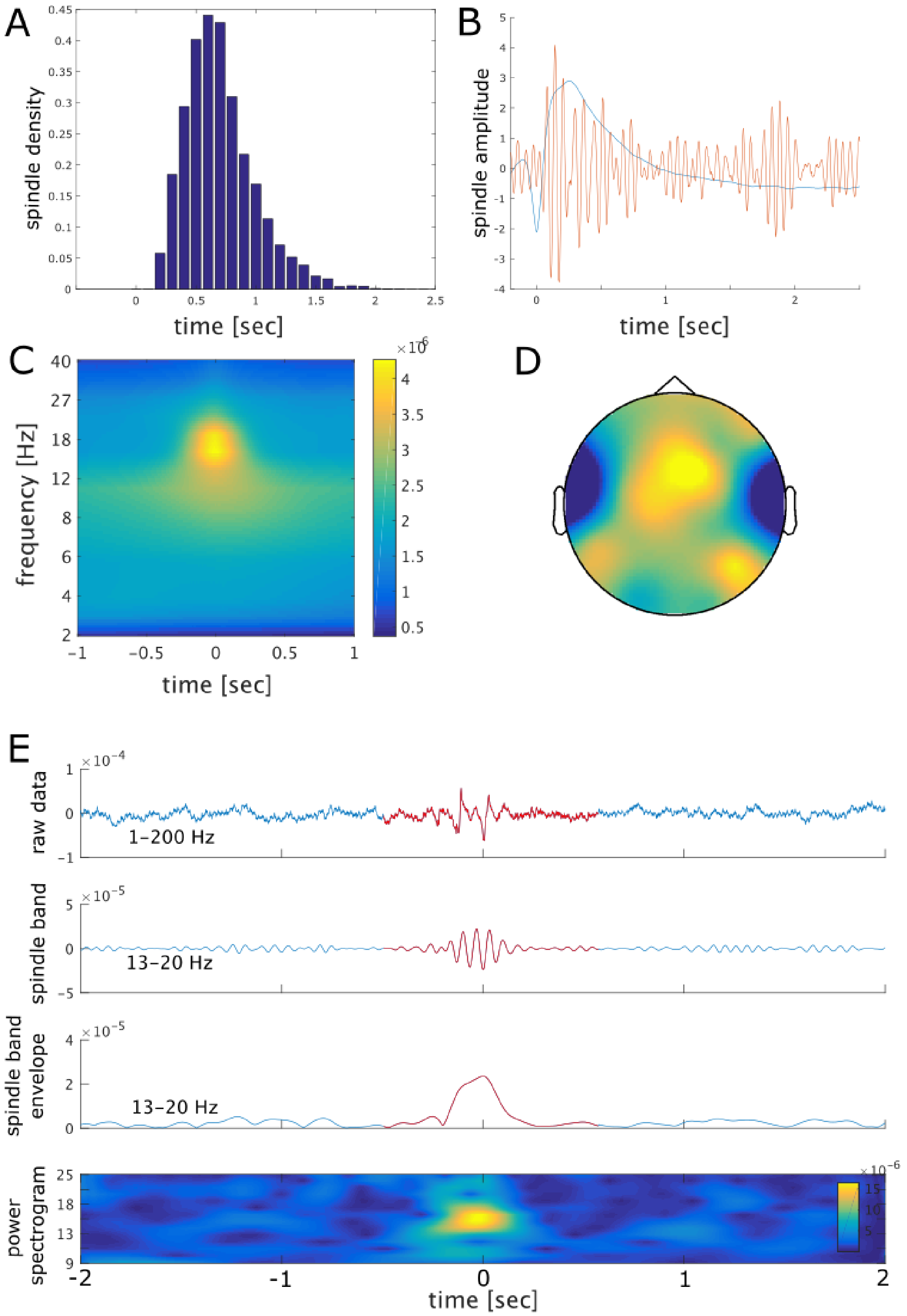

Figure R4 | Wake spindle in EEG. A: The spindle distribution bar (spindles/minute). ***B***: Zscored averaged raw signal (orange line) according to the spindle onset and the Zscored averaged amplitude

(blue line). ***C***: The averaged time frequency of spindle according to the spindle peak. ***D***: The topography of spindle events. ***E***: An example of wake spindle.

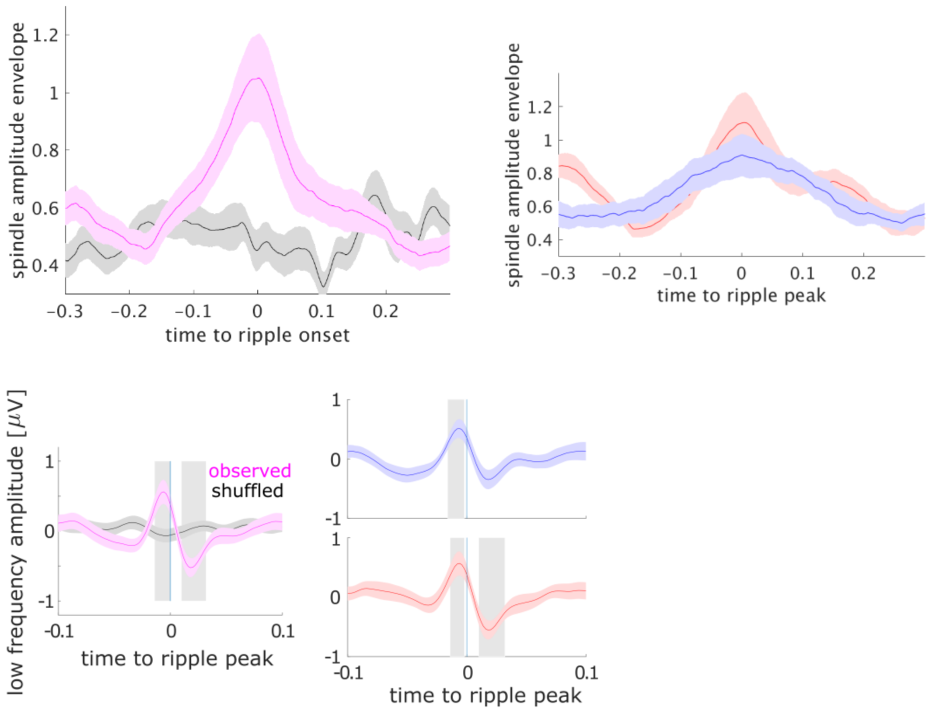

Figure R5 | The wake spindle amplitude envelope centered around ripples. The pink line represents the grand-averaged spindle band, while the gray line represents the shuffled spindle band. The red line indicates the PA condition, and the blue line indicates the rest condition.

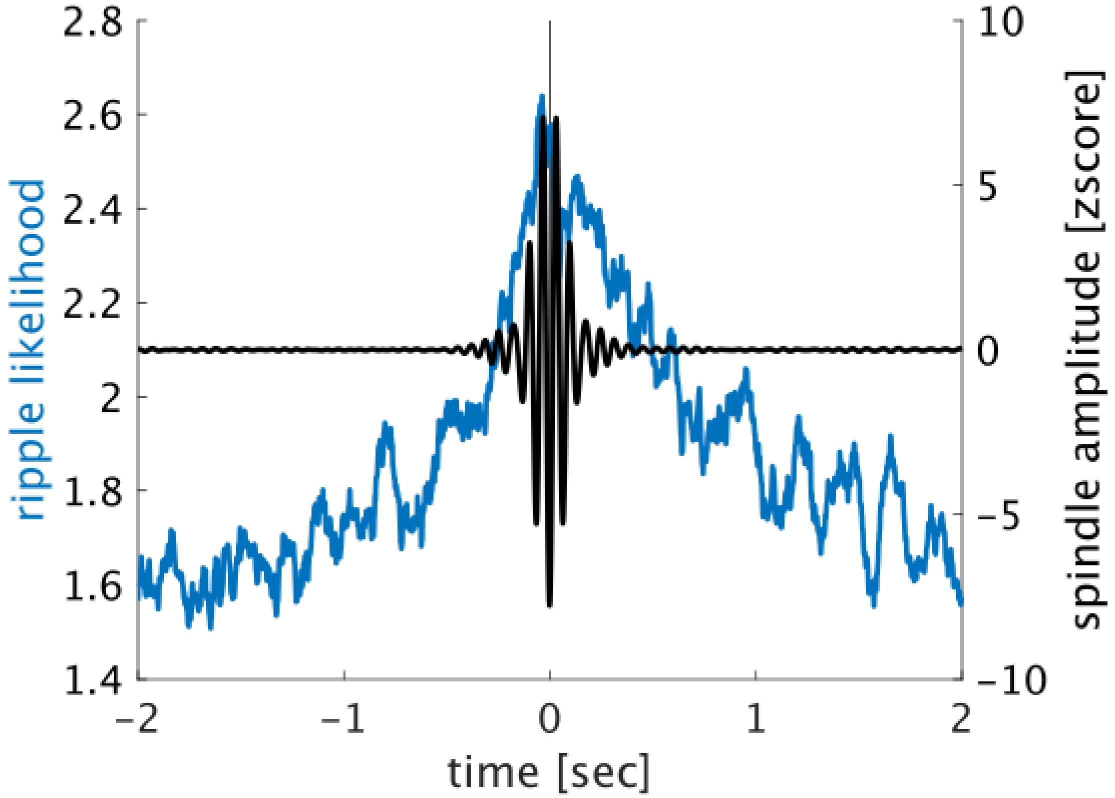

Figure R6 | Spindle centered ripple likelihood.

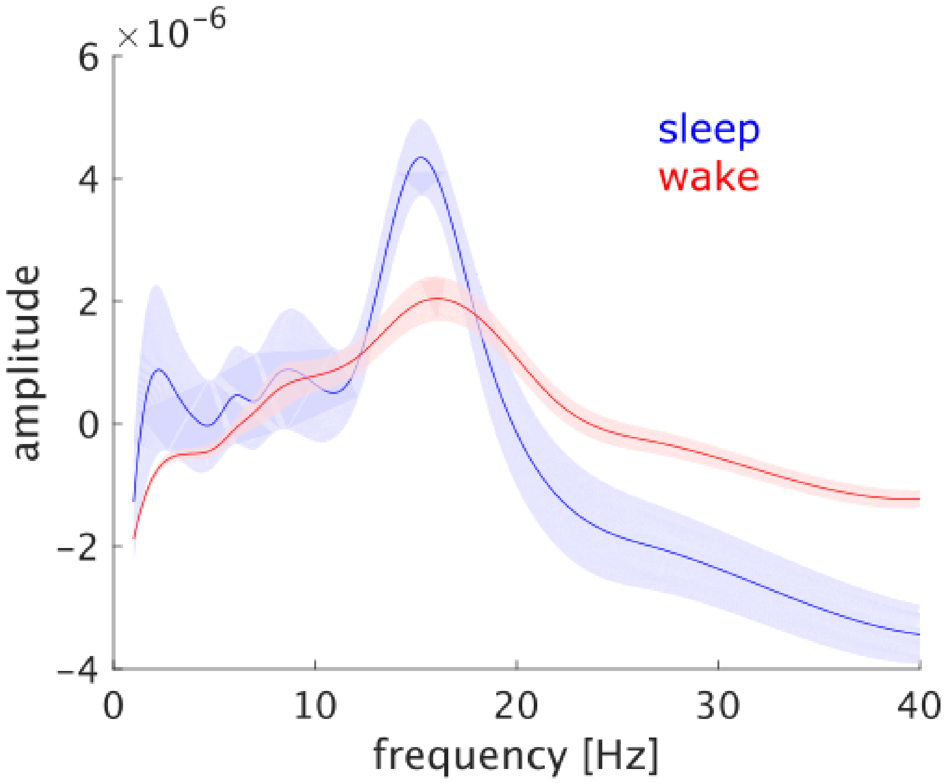

Figure R7 | The frequency amplitude at the spindle peak.

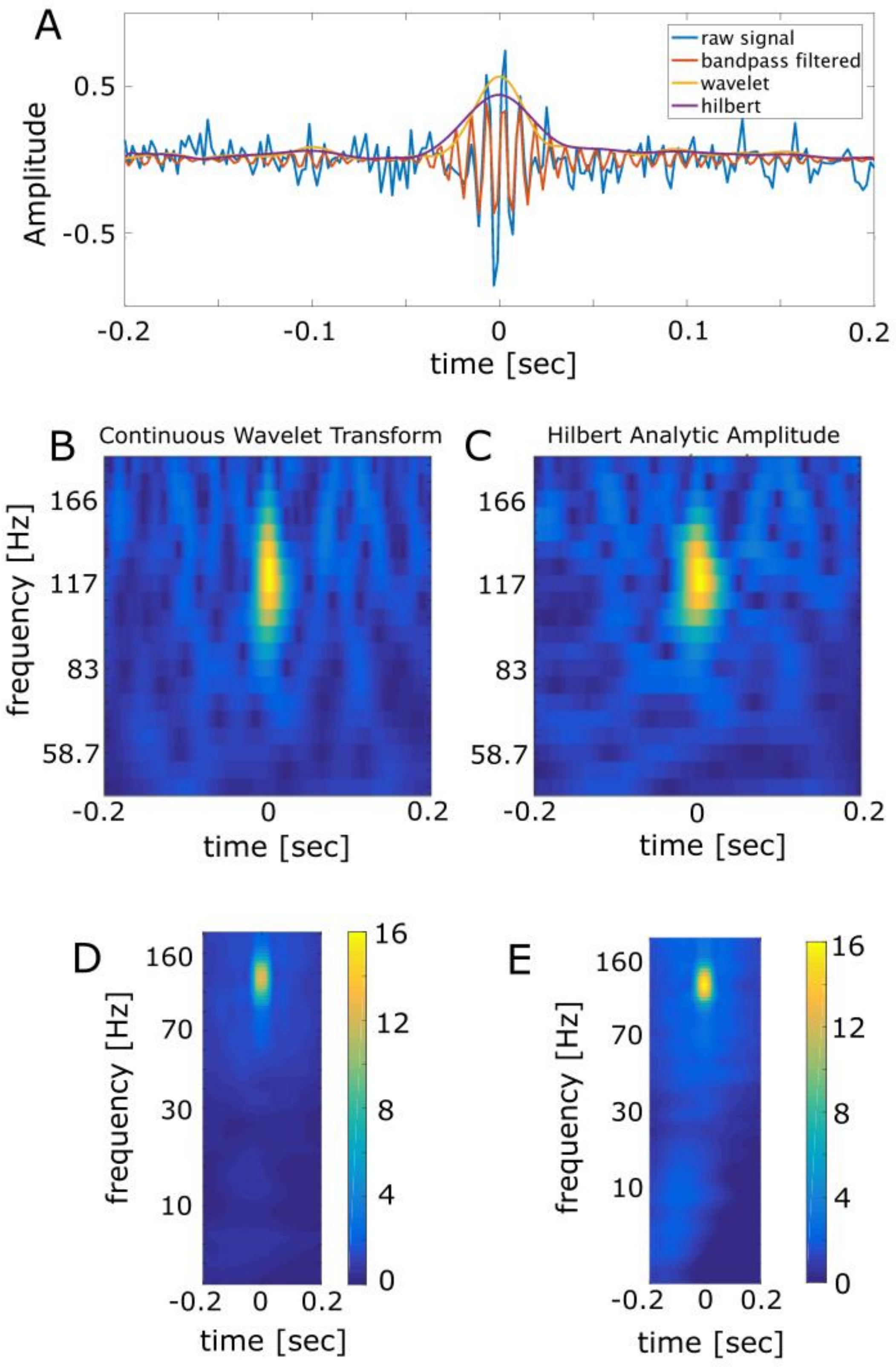

Figure R8 I Comparison of the continuous wavelet transform (CWT) and the Hilbert transform method. A: An artificially generated 120Hz burst signal, the bandpass-filtered signal (105-130Hz) and the analytical amplitude of the Hilbert transform of the bandpass-filtered signal as well as the CWT at center frequency 117.4Hz. ***B***: The time-frequency representation of the simulated 120Hz burst with CWT. ***C***: The time-frequency representation of the simulated 120Hz burst with Hilbert transform. ***D***:

The time-frequency representation of ripples with CWT (control analysis). ***E***: The time-frequency representation of ripples with Hilbert transform (same as in paper).

References：

1. Andreotti, D. Z. et al. Effects of Physical Exercise on Autophagy and Apoptosis in Aged Brain: Human and Animal Studies. Front. Nutr. **7**, 94 (2020).
2. Mersy, D. J. Health benefits of aerobic exercise. Postgraduate Medicine **90**, 103–112 (1991).
3. Phillips, S. M. & Winett, R. A. Uncomplicated Resistance Training and Health-Related Outcomes: Evidence for a Public Health Mandate. Current Sports Medicine Reports **9**, 208–213 (2010).
4. Van Praag, H., Kempermann, G. & Gage, F. H. Running increases cell proliferation and neurogenesis in the adult mouse dentate gyrus. Nat Neurosci **2**, 266–270 (1999).
5. Puce, A. & Hämäläinen, M. A Review of Issues Related to Data Acquisition and Analysis in EEG/MEG Studies. Brain Sciences **7**, 58 (2017).
6. Audiffren, M. Acute Exercise and Psychological Functions: A Cognitive-Energetic Approach. in Exercise and Cognitive Function (eds. McMorris, T., Tomporowski, P. D. & Audiffren, M.) 1–39 (Wiley, 2009). doi:10.1002/9780470740668.ch1.
7. Sibley, B. A. & Beilock, S. L. Exercise and Working Memory: An Individual Differences Investigation. Journal of Sport and Exercise Psychology **29**, 783–791 (2007).
8. Pontifex, M. B., Hillman, C. H., Fernhall, B., Thompson, K. M. & Valentini, T. A. The Effect of Acute Aerobic and Resistance Exercise on Working Memory. Medicine & Science in Sports & Exercise **41**, 927–934 (2009).
9. Davey, C. P. Physical Exertion and Mental Performance. Ergonomics **16**, 595–599 (1973).
10. Covassin, T., Weiss, L., Powell, J. & Womack, C. Effects of a maximal exercise test on neurocognitive function. Br J Sports Med **41**, 370–374 (2007).
11. Dietrich, A. & Sparling, P. B. Endurance exercise selectively impairs prefrontal-dependent cognition. Brain and Cognition **55**, 516–524 (2004).
12. Suwabe, K. et al. Rapid stimulation of human dentate gyrus function with acute mild exercise. Proc. Natl. Acad. Sci. U.S.A. **115**, 10487–10492 (2018).
13. Quelhas Martins, A., Kavussanu, M., Willoughby, A. & Ring, C. Moderate intensity exercise facilitates working memory. Psychology of Sport and Exercise **14**, 323–328 (2013).
14. Abou Khalil, G., Doré-Mazars, K., Senot, P., Wang, D. P. & Legrand, A. Is it better to sit down, stand up or walk when performing memory and arithmetic activities? Exp Brain Res **238**, 2487–2496 (2020).
15. McKenzie, S., Nitzan, N. & English, D. F. Mechanisms of neural organization and rhythmogenesis during hippocampal and cortical ripples. Phil. Trans. R. Soc. B **375**, 20190237 (2020).
16. Helfrich, R. F. et al. Bidirectional prefrontal-hippocampal dynamics organize information transfer during sleep in humans. Nat Commun **10**, 3572 (2019).
17. Cardenas, A. R. et al. Exercise Modulates Human Hippocampal-Cortical Ripple Dynamics. http://biorxiv.org/lookup/doi/10.1101/2023.05.19.541461 (2023) doi:10.1101/2023.05.19.541461.
18. Kandel, A. & Buzsáki, G. Cellular–Synaptic Generation of Sleep Spindles, Spike-and-Wave Discharges, and Evoked Thalamocortical Responses in the Neocortex of the Rat. J. Neurosci. **17**, 6783–6797 (1997).
19. Staresina, B. P., Niediek, J., Borger, V., Surges, R. & Mormann, F. How coupled slow oscillations, spindles and ripples coordinate neuronal processing and communication during human sleep. Nat Neurosci **26**, 1429–1437 (2023).
20. Petzka, M., Chatburn, A., Charest, I., Balanos, G. M. & Staresina, B. P. Sleep spindles track cortical learning patterns for memory consolidation. Current Biology **32**, 2349-2356.e4 (2022).
21. Pavlov, Y. G. & Kotchoubey, B. EEG correlates of working memory performance in females. BMC Neurosci **18**, 26 (2017).
22. Aritake-Okada, S. et al. Diurnal repeated exercise promotes slow-wave activity and fast-sigma power during sleep with increase in body temperature: a human crossover trial. Journal of Applied Physiology **127**, 168–177 (2019).
23. Frisch, N. et al. An acute bout of high-intensity exercise affects nocturnal sleep and sleep-dependent memory consolidation. Journal of Sleep Research e14126 (2023) doi:10.1111/jsr.14126.
24. Link, Λ., Elster, C., Sander, T., Lucschow, A. & Curio, G. MEC-ANALYSIS USING THE HILBERT TRANSFORM. Biomedizinische Technik (2002).
25. Agarwal, P. & Kumar, S. EEG-based imagined words classification using Hilbert transform and deep networks. Multimed Tools Appl **83**, 2725–2748 (2024).
26. Emara, H. M. et al. Hilbert Transform and Statistical Analysis for Channel Selection and Epileptic Seizure Prediction. Wireless Pers Commun **116**, 3371–3395 (2021).

27. Bruns, A. Fourier-, Hilbert- and wavelet-based signal analysis: are they really different approaches? Journal of Neuroscience Methods **137**, 321–332 (2004).
28. Le Van Quyen, M. et al. Comparison of Hilbert transform and wavelet methods for the analysis of neuronal synchrony. Journal of Neuroscience Methods **111**, 83–98 (2001).
29. Latchoumane, C.-F. V., Ngo, H.-V. V., Born, J. & Shin, H.-S. Thalamic Spindles Promote Memory Formation during Sleep through Triple Phase-Locking of Cortical, Thalamic, and Hippocampal Rhythms. Neuron 95, 424-435.e6 (2017).
30. Staresina, B. P. et al. Hierarchical nesting of slow oscillations, spindles and ripples in the human hippocampus during sleep. Nat Neurosci 18, 1679–1686 (2015).
31. Norman, Y. et al. Hippocampal sharp-wave ripples linked to visual episodic recollection in humans. Science 365, eaax1030 (2019).
32. Muthukumaraswamy, S. D. High-frequency brain activity and muscle artifacts in MEG/EEG: a review and recommendations. Front. Hum. Neurosci. **7**, (2013).

The authors did not address most of my major comments adequately in the revised manuscript.
-The main results of this manuscript are based on “awake” spindles, however, as I explained in details previously, there is no compelling evidence of the existence of spindles during the awake state. In the revised manuscript, the authors stated that “it has been reported that the neural sink and source of wake spindles are similar to those of sleep spindles (20)”. However, in this old reference (20) which is the only reference provided by the authors for the existence of “wake” spindles, only the “sleep” spindles with “high voltage spike-and-wave patterns (HVSs)” were compared.

Reply: We appreciate the reviewer’s detailed comments and have carefully revised our manuscript to address these concerns. We acknowledge that thalamocortical events observed during wakefulness have historically been referred to as high-voltage spike-and-wave patterns (HVSs). However, studies suggest that these events share similar neural origins and mechanisms with sleep spindles (Reference 20) (Petzka et al., 2022). While we agree that compelling evidence for fully equivalent "wake spindles" is still under investigation, our findings contribute to this discussion by demonstrating oscillatory events with spindle-like characteristics in the awake state.

The authors conducted experiments to compare the properties of sleep and “wake” spindles and stated that both spindles mostly occur in the fronto-central region. However, the topographical distribution shown in figure R3 D and R4 D are not similar, and also there is no color bar in these figures to compare the spindle density between them.

Reply: we agree with the figures are not identical: one represents spindle detection during the working memory paradigm in rest phases after stimulus presentation, while the other shows spindles during sleep. Nevertheless, both figures reveal a strong concentration of spindles in a fronto-central ROI, where usually high amplitude spindles can be found (Petzka et al., 2022).

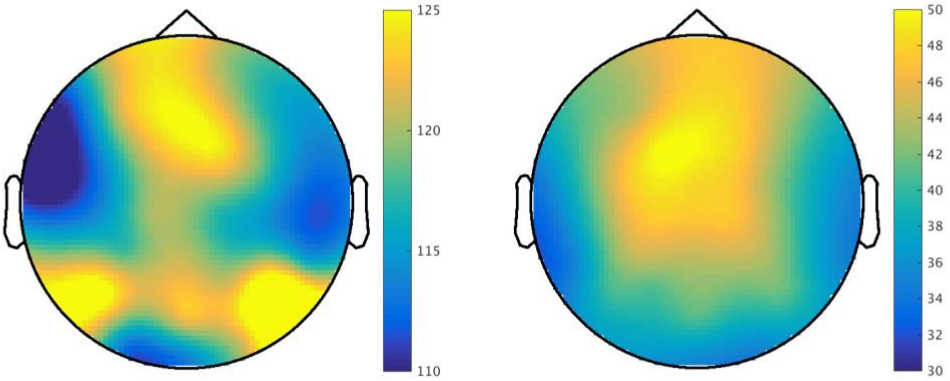

***Figure R1* | Topographical distribution of spindle events.** Left: Results from the current study. Right: Results from a sleep deprivation study.

The distribution of large amplitude spindles is largely overlapping with other studies showing that fast and slow spindle density can be found at fronto-central ROIs (Fernandez and Lüthi, 2020; Petzka et al., 2022).

[figure redacted]

Spindle topography from reference (Fernandez and Lüthi, 2020).

[figure redacted]

Group-level topographies of amplitude, duration, and density of sleep spindles and slow oscillations (Petzka et al., 2022).

More importantly, using a simple method of detection to find the high amplitude activity in 13-20 Hz from the EEG in a brain state does not provide compelling evidence for the existence of the spindles with the known thalamocortical mechanisms in that state.

Reply: In our previous response letter, we explained that we used the same detection method for spindles as for ripples to ensure signal comparability. As outlined below, we employed essentially the same analysis which has been employed by several groups. Burst-like activity, including spindles, can be reliably identified using algorithmic detection (Ayoub et al., 2012; Petzka et al., 2022; Schreiner et al., 2021; Staresina et al., 2023). The authors of these studies also used band-pass filtering at different frequency ranges—12–16 Hz, 12–18 Hz, 12–15 Hz,

and 12.5-15.5Hz, respectively. Notably, applying such frequency bands results in the detection of spindles within a broader range, typically between 10 and 20 Hz (see Figure 2A in Schreiner et al., 2021, Figure 1E in Petzka et al., 2022 and Figure 1 in Ayoub et al., 2012). These studies followed a similar approach: the authors calculated the root mean square (RMS) over a 200-ms window, followed by smoothing with the same window length. A spindle event was identified when the smoothed RMS signal exceeded a threshold defined as the mean plus one standard deviation, and spindle duration was required to be between 0.4 and 3 sec. In our approach, we used the signal envelope derived from the Hilbert transform, which provides a more accurate instantaneous estimate than the RMS. Furthermore, we applied a threshold higher than one standard deviation. Therefore, our approach is comparable to that of the other authors. We fully agree with the reviewer that EEG cannot provide precise insights into thalamocortical mechanisms.

Also, the example of the detected spindle shown in figure 6 does not look like a typical spindle in terms of duration and waveform shape.

Reply: We assume that the reviewer refers to the first graph, where we present a raw signal. It is important to note that this is not directly comparable to spindles observed in the EEG during sleep, as the ongoing signal is often filtered below 40 Hz during EEG monitoring (Andrillon et al., 2011). However, the filtered signal does exhibit burst-like oscillatory activity with a waxing and waning modulation, similar to the patterns described by Staresina, without explicitly identifying it as spindle activity (Staresina et al., 2023). In general, we agree with the reviewer that a different spindle event with a more pronounced waxing and waning phase would be more suitable (see ***Fig. 6***).

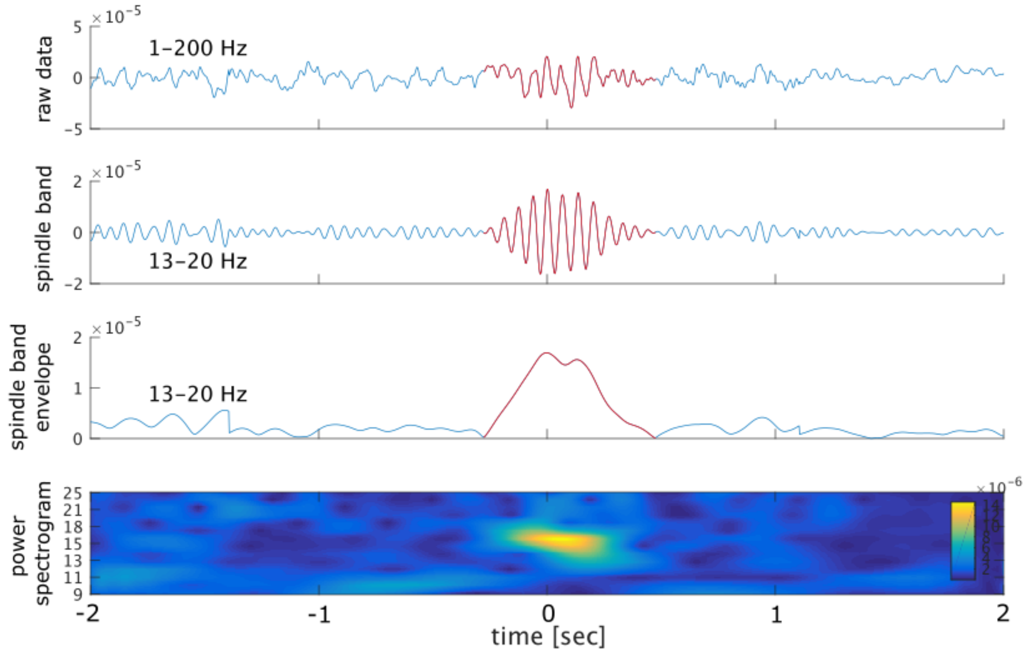

Fig. 6 | spindle activation in EEG.
An example of spindle detected in our analysis. The upper line shows the broad band signal between 1 and 200 Hz. The second time series shows the same signal in the spindle band. The third time series shows the Hilbert transform of the spindle band activity. The last graphic shows the time frequency representation of the signal filtered between 13-20 Hz.

There are several concerns with the spindle detection method that was used in this manuscript which have been mentioned previously but the authors did not adequately address them in the

revised manuscript or the response letter:

1- Spindle frequency: They authors called very different ranges of frequencies as the “wake” spindles but none of them corresponds to the mostly used frequency range for spindles. For example, the authors stated that “We found a consistent temporal pattern across both sessions, with spindle activity decreasing immediately after stimulus onset, followed by a subsequent increase at fronto-central EEG electrodes”. However, the frequency range of the decrease and increase of the activity are very different. The reduction is concentrated around 9-10 Hz but the increase is from around 14 Hz up to more than 20 Hz. The authors called all of this wide range as “wake spindles” without any further evidence.

Reply: The reviewer refers to ***Fig. 2A***. It is correct that the strongest decrease is observed in the frequency range between 9 and 10 Hz. However, as shown in ***Fig. 2B***, a decrease is also present in the band above 10 Hz.

In the previous review the reviewer wrote: the Authors considered different frequency bands for spindles (14-18 Hz for figure 2, 14-16 Hz for figure 5B, 13-20 Hz for spindle detection, line 446) but none of them is consistent with the established spindle frequency range (10-16 Hz).

Reply: The reviewer is correct in noting that the spindle frequency ranges we considered to not fully align with the traditionally established 10–16 Hz range. However, in our study, we used a data-driven approach to address potential discrepancies between previous sleep studies and our working memory study. In ***Fig. 5A***, we show that EEG activity is locked to ripple peaks. Specifically, we found a low-frequency oscillation with a peak at approximately -6 ms and a trough at ~24 ms relative to the ripple peak. Since the peak and trough are ~30 ms apart, the full cycle lasts ~60 ms, corresponding to a frequency of 16 Hz. This indicates a 16 Hz oscillation around ripples, making it reasonable to analyze this activity within a frequency band centered around 16 Hz. Please note that the actual frequency spectrum of detected spindles is between 10 and 20Hz with a maximum around 15Hz (Schreiner et al., 2021; Petzka et al., 2022; Ayoub et al., 2012). The 16 Hz was the starting point for our further analyses. We then tested whether this exact frequency range showed a modulation with stimulus onset and took a frequency band around 16 Hz with a band width of 4 Hz (14-18 Hz), which may reflect specific neural dynamics overlapping with the conventional spindle range. Given that wake spindles are controversial and less well-characterized than sleep spindles, we adopted this data-driven approach to capture the specific characteristics of this relatively new signal. As a result, the differences from previous sleep studies are to be expected. However, we observed significant overlap between wake and sleep spindles. To clarify this distinction, we have decided to use the term "wake spindles" throughout the article to differentiate them from sleep spindles.

In response to my previous comment, the authors stated that “Additionally, we compared the frequency amplitude at the spindle peak and found that the main frequency range was similar between sleep and wake spindles (see Figure R7)”. However, this similarity is due to using the same frequency range of 13-20 Hz to detect sleep and wake spindles and does not directly address my concern.

Reply: We appreciate the reviewer’s concern and would like to clarify the methodology behind ***previous Figure R7***. We first detected spindle events using EEG signals between 13-20 Hz from electrodes in the central regions. We then extracted EEG epochs from -0.5 to 0.5 sec, centered on spindle events, and conducted a time-frequency analysis spanning 1 to 40 Hz. Finally, we plotted the amplitude of different frequencies at the spindle peak.
Our results indicated that the average sleep spindle peaks at 15.17 Hz. To test that this peak frequency is not due to 13–20 Hz detection range, we ran the same analysis again applying

the 10–16 Hz frequency range as suggested by the reviewer. The results are shown in the new figure ***Figure R2***. In this figure, we compare spindle activity from our sleep study using both the 10–16 Hz range and our 13–20 Hz range. Peak frequencies do not differ between the different frequency bands applied. These findings indicate that finding a peak around 16Hz is not the result of the chosen frequency band.

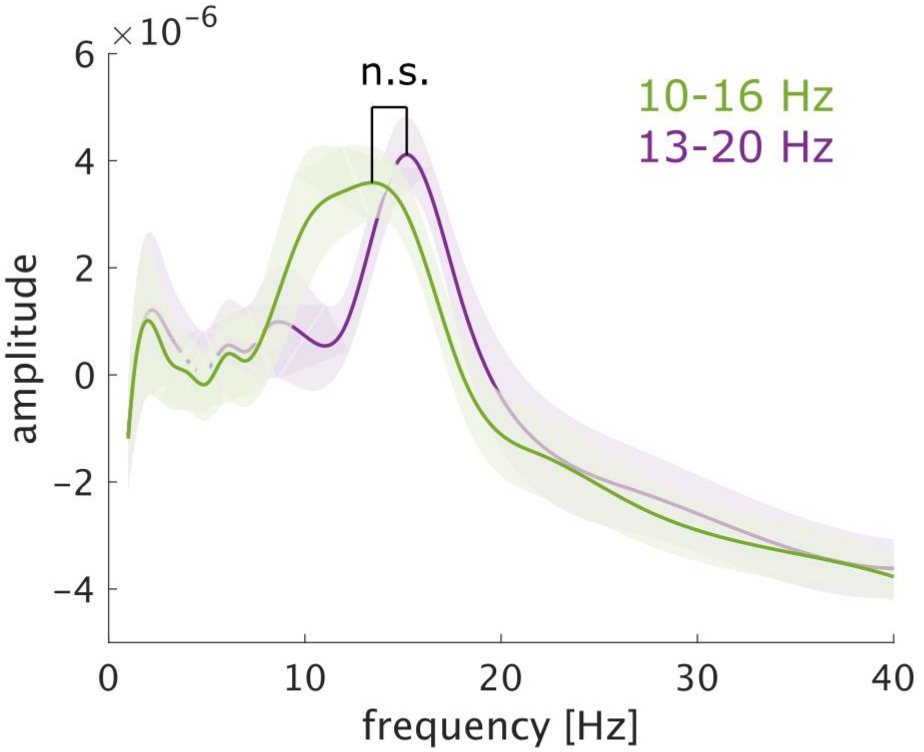

***Figure R2* | The frequency amplitude at the sleep spindle peak under different band-pass filters.**

For spindle-ripple coupling, the authors considered a wide range of frequencies from 1 to 25 Hz. To address this concern, in the response letter, the authors wrote: "Our analysis, which includes a broader frequency range, highlights the robustness of spindle activity despite higher amplitudes at lower frequencies. Notably, oscillations in the spindle band emerge as significant.". However, the peak and through of the modulated low frequency activity around ripples are of the order of 10 ms apart and there are no oscillations in the spindle frequency band.

Reply: In ***Fig. 5A***, we show coupling of EEG ongoing activity to ripple peaks. Around ripples, we observe a clear oscillatory pattern. This oscillation demonstrated a significant positive amplitude modulation with a peak at -6 ms and a significant trough at 24 ms around the ripple peaks as outlined in our results. The time between peak and trough is approximately 30 ms, meaning the entire cycle lasts 60 ms. This corresponds to a frequency of 16 Hz, indicating a 16 Hz oscillation around ripples. This is in line with the spindle band found in previous studies (Ayoub et al., 2012; Schreiner et al., 2021). It seemed reasonable to analyze this activity within a frequency band around 16 Hz. Our analysis of spindle frequency in the sleep study, using both 10–16 Hz and 13–20 Hz bands, reveals a peak around 16 Hz for spindles during sleep independently of the chosen frequency band.

We merged the ***Figure R3*** into ***Fig. 5A***.

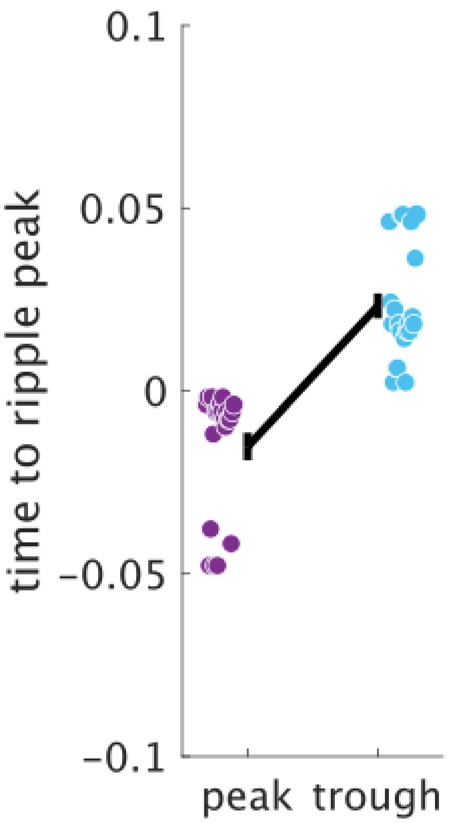

***Figure R3* | Grand-averaged slow oscillations around the ripple peak.** The slow oscillation amplitude peaks and troughs from individual subjects. The purple dots represent amplitude peaks in the EEG relative to ripple peak, while the cyan dots represent troughs in the EEG relative to ripple peak.

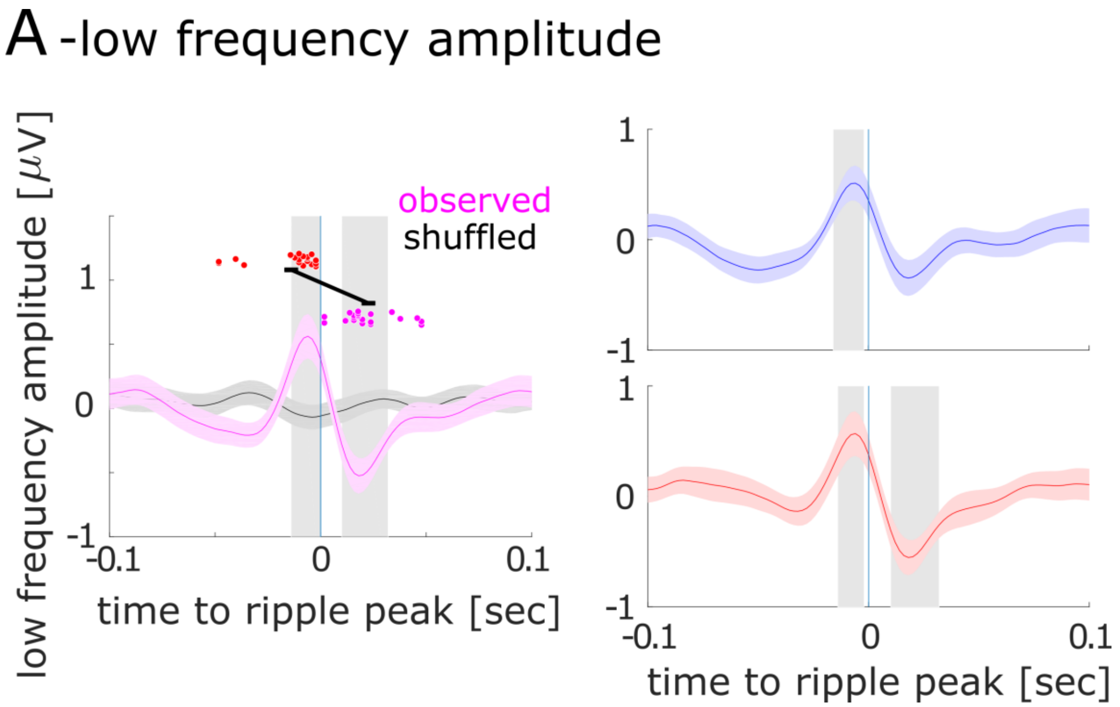

Fig. 5 | MEG-Ripple and EEG low-frequency coupling results.

A (left): Grand-averaged slow oscillations around the ripple peak compared to the shuffled peak. The dots at the top represent slow oscillation amplitude peaks and troughs from individual subjects. The red dots indicate amplitude peaks, while the pink dots indicate troughs. (right): The slow oscillations around the ripple peak from different sessions. The blue color represents the rest session, and the red color represents the PA session.

In figure R5 where they used the spindle frequency range to determine the spindle modulation around the ripples, it seems that there is no significant difference between the PA and rest conditions.

Reply: In the ***previous Figure R5*** and ***Fig. 5*** of the manuscript, we demonstrate an oscillatory EEG pattern around the MEG ripples, with a peak for the ripples and a significant trough following the ripples. We also show that this trough is solely due to a modulation following physical activity. Additionally, ***Fig. 5B and C*** and the results section in the manuscript provide further evidence of stronger coupling between ripples and spindles following physical activity compared to rest.

2- Spindle duration: It is very important to consider the minimum duration of 0.5 s for the spindle detection (similar to almost all the previous articles that detected spindles), otherwise, as is also observed in figures R3 & R4 A, a large portion of the events would be very short ones which are not necessarily related to the spindle activity. To address this previously mentioned comment, the authors stated in the response letter: “To address this, we analyzed the frequency distribution of spindle lengths and found that the events averaged over 500 ms in duration”. However, an average value over 0.5 s is not sufficient and there should be no event below 0.5 s.

Reply: A minimum duration of 500 ms is not always assumed (see for example Staresina et al., 2023) and depends on the cutoff criterion applied. When a burst is assessed based on how long it exceeds a certain amplitude threshold, the duration above that criterion decreases as the threshold is raised. For example, in their study, Staresina et al. included all spindles that exceeded a 1 SD threshold for at least 400 ms. In contrast, we applied a more stringent SD criterion, which naturally results in shorter events. Had we used a 1 SD criterion, the events would likely have been longer. We acknowledge that the typical duration of sleep spindles is generally longer than 0.5 sec. In our analysis, the majority of detected sleep spindles exceeded 0.5 sec, with a mean duration of 0.945 sec, suggesting that our detection method aligns with established criteria.

3- Spindle topographical distribution: The authors claimed that (fast) spindle activity was typically seen in fronto-central regions similar to the previous observations and they referred to only one reference (12). However, in this paper (reference 12), consistent with many other papers (for example see doi:10.1523/JNEUROSCI.2604-11.2011 and 10.1152/physrev.00042.2018) the authors clearly stated that “At the group level, spindles and SOs showed the established prevalence over centro-parietal and frontal areas, respectively”.

Reply: In the paper noted, invasive EEG recordings were obtained using microwires and depth electrodes. The EEG was monitored through four surface electrodes—C3, C4, Pz, and Fz—to track sleep stages. However, the location and timing of the signals were primarily based on data from the depth electrodes. It is important to note that the EEG topography cannot be clearly determined from these depth electrodes alone, as deep sources do not directly project to surface EEG sensors. For instance, the direction of an auditory dipole typically results in stronger modulation at Cz. Therefore, the terms "posterior" and "frontal" refer to the activity observed at the depth electrode level, rather than at the surface EEG

electrode locations.
Petzka et al. demonstrate that spindles are most prevalent over centroparietal areas, while slow oscillations are more common in frontal regions, as shown in their Supplementary Figure 2 (Petzka et al., 2022). While their findings confirm that spindles predominantly occur at centroparietal sensors, they also indicate that high-amplitude spindles are more frequently observed at frontocentral electrodes (see supplementary Figure 2).
Our analysis focused on these high-amplitude events for two reasons. First, we identify spindles based on ripple-initiated EEG bursts. Since spindle events can exhibit slight temporal shifts, high-amplitude events are less affected by this variability, making them more reliably detectable—particularly in frontal regions, where high amplitude spindles are more prevalent. Second, we applied a high-amplitude threshold, further refining our analysis to regions with strong spindle activity. This approach ensures a targeted investigation of high-amplitude spindle dynamics.

4- In figure S2 of this paper it has been shown that the fast spindle activity (density) is dominant across centro-parietal regions. The "slow" spindles occur mostly at fronto-central regions; however, the frequency range that the authors have considered for the spindle (13-20 Hz) is very different from the slow spindle frequency range (8-12 Hz).

Reply: As detailed above, the reviewer noted a study based on invasive depth electrodes. However, it is important to note that scalp EEG alone cannot unambiguously resolve activity patterns in deep regions. Unfortunately, the study also did not provide EEG patterns demonstrating whether frontal slow spindles and fast centro-parietal spindles can be dissociated in the EEG. Consequently, it cannot be ruled out that the centro-parietal deep regions may contribute to the EEG patterns we observed.
We appreciate the reviewer's comments and have carefully re-examined the referenced study. This study provides a detailed topographical distribution of spindle density, and our findings align more closely with the distribution of fast spindles, which can reach frequencies up to 17 Hz. As mentioned previously, we believe there may be variations between wake and sleep spindles, and even sleep spindles can exhibit distinct topographical variations across different sleep stages and among different subjects (Fernandez and Lüthi, 2020; Zeitlhofer et al., 1997). In response to the reviewer's feedback, we have revised our manuscript to specify that the electrode used for spindle analysis is located in the central region.

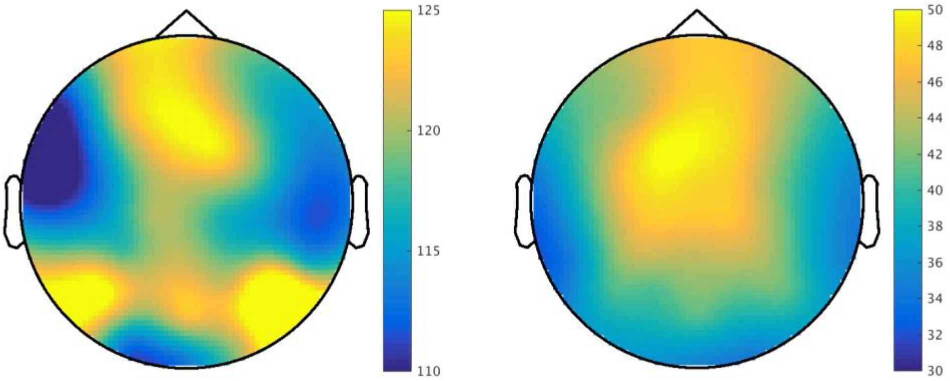

***Figure R1* | *t*opographical distribution of spindle events.** Left: Results from the current study. Right: Results from a sleep deprivation study.

-The authors found no direct evidence for different strengths of spindle-ripple coupling in the PA condition compared to the rest condition and hence it cannot be concluded that the PA is

associated with enhanced spindle-ripple coupling. However, they stated this claim in several sections of the manuscript:
abstract: “These findings reveal that PA enhances WM by coordinating ripple-spindle Interaction”, introduction: “Critically, PA increased coupling of MEG-ripples to EEG-spindle activity and also enhanced WM performance.”, discussion: “Our results indicate that short-term PA enhances spindle-ripple coupling, contributing to improved memory formation.”

Reply: The ***Fig. 5B and C*** in the manuscript provide evidence for coupling differences between the PA and rest conditions. Based on the reviewer’s feedback, we revised the abstract, introduction, and discussion to ensure that our conclusions more accurately reflect the strength of the evidence. Instead of stating that PA definitively enhances spindle-ripple coupling, we indicate that PA is associated with a modulation of spindle-ripple interactions, rather than a confirmed enhancement.
For example:
Neurophysiologically, PA scaled the ripple rate with the number of items to be kept in WM and strengthened the coupling between ripple events and spindle oscillations. These findings reveal that PA enhances WM and modulates ripple-spindle interaction.

-The authors investigated the ripple activity after the onset of stimulus. However, ripples are believed to occur mostly during the rest state not during the stimulus. Hence the decrease in the ripple activity they found after the stimulus onset is not novel.

Reply: We appreciate the reviewer’s comment and would like to clarify our rationale. Our study specifically examines the temporal evolution of ripple activity in response to stimulus onset. While it is well established that ripples predominantly occur during rest, it is important to characterize how ripple activity is modulated by external stimuli. The observed decrease in ripple activity post-stimulus provides a baseline for analyzing subsequent rebound effects and for comparing PA and rest conditions. We acknowledge that ripple suppression after stimulus onset may not be a novel finding, but its precise temporal dynamics in the context of PA have not been well characterized. Our analysis contributes to a more comprehensive understanding of how ripples evolve following a stimulus, particularly in relation to PA.

-In the discussion, it has been written: “We observed spindle activity in both PA and rest sessions, topographically overlapping with sleep spindle findings in previous studies12. Notably, the amplitude of sleep spindles increased after the working memory task facilitating memory consolidation”. However, no measure of memory consolidation/retention has been conducted in this study and the behavioral performance has measured only once.

Reply: The reviewer is correct that in this study, we focused on working memory and did not assess memory consolidation across a retention interval. Hence, we removed the link to memory consolidation in that sentence.

The new sentence now reads:
We observed spindle activity in both PA and rest sessions, topographically overlapping with sleep spindle findings in previous studies[12]. Notably, spindle band amplitude increased after the working memory task.

-I am not convinced with authors explanation about using correlation analyses between only 3 data points instead of ANOVA to find the association between ripple density and different N-back conditions.

Reply: We initially excluded this analysis from the manuscript because it did not provide insights into the underlying model and did not inform about the concept of scaling.

In response to the reviewers concerns, we refined the analysis in two ways. First, we demonstrate that the ANOVA reveals a significant main effect of the N-back condition. We performed a one-way ANOVA to examine the differences in the number of ripples between the N-back conditions. The ANOVA revealed a significant difference in ripple density between different N-back conditions following PA ($F = 3.24$, $p = 0.046$), but not in the rest session ($F = 0.84$, $p = 0.4371$). Second, we introduce a novel approach to directly examine scaling. Specifically, we ask whether inter-individual differences remain consistent across N-back conditions indicating that subjects with a higher number of ripples in one condition also have a higher number of ripples in another condition. To test this, we computed Pearson correlation of ripple counts across participants between different N-back blocks (2 vs. 3, 2 vs. 4, and 3 vs. 4), applying Bonferroni correction for multiple comparisons. Scaling was observed only after PA.

Additionally, we conducted a correlation analyses to assess the relationship between ripple density from different N-back conditions (see ***Figure R4***). Our results indicate that ripple density exhibited a stronger positive correlation following PA compared to rest, suggesting a tighter association between ripple density and memory load after PA.

	2-3 back	2-4 back	3-4 back
rest	R=0.5468; p=0.0115	R=0.2922; p=0.1981	R=0.5468; p=0.0115
PA	R=0.7156; P<0.001	R=0.6740; P=0.0011	R=0.6208; P=0.0033

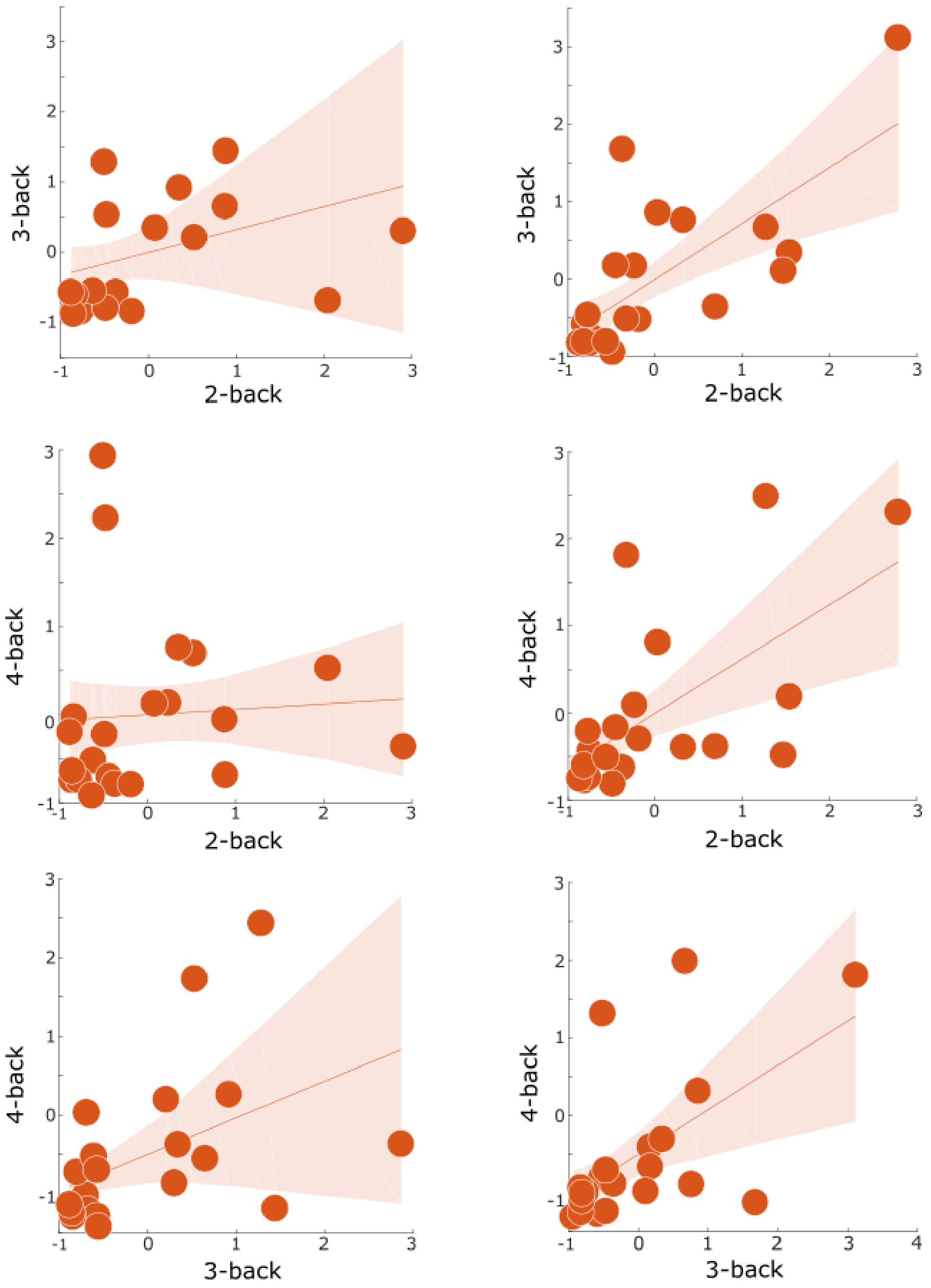

Figure R4 **| The correlation of ripple density across different N-back conditions.** The left column represents the correlation during the rest session, while the right column represents the correlation during the PA session.

Here we added the ANOVA and correlation results to the results and methods section.

Result:

We then tested whether the ripple frequency predicted individual hit rate. We found no correlation in the first sec after stimulus presentation (r = 0.17; p = 0.46). However, ripple rate between 1.5-2.5 sec predicted individual target detection rate (r = 0.49; p = .021) (see ***Fig. 4B***). In the first step we compared ripple rate in this interval between conditions but did not find a difference (t_{20} = .43; p = .67). <u>When we compared the ripple density between conditions we found a significant difference between N-back conditions following PA (F = 3.24, p = 0.046)</u>

but not following rest (F = 0.84, p = 0.437). Furthermore, we found that the ripple density across participants was strongly correlated between blocks following PA ($p_{corrected}$ = .05/6 = .0083; 2 vs 3-back: r = 0.7156; 2 vs 4-back: r = 0.6740; 3 vs 4-back: r = 0.620; all p-values < .0034) but not following rest (2 vs 3-back: r = 0.546; 2 vs 4-back: r = 0.2922; 3 vs 4-back: r = 0.5468; all p-values > .01). This result indicates that inter-individual differences remain consistent across N-back conditions following PA but not rest. Next, we tested whether the number of ripples is scaled with the number of items to be held in working memory (see ***Fig. 4C***). We conducted a correlation analysis between N-back and ripple rate for each participant leading to a correlation value for each subject. Using directed t-tests under the assumption that r-values are higher than zero (more ripples when more items are held in WM) we found a positive linear scaling of N-back and ripples in the PA session (r_{PA} = 0.77; t_{20} = 1.86; p = 0.038). No such effect was observed in the rest session (r_{Rest} = -0.05; t_{20} = 0.12; p = 0.452). The N-back ripple correlation in the PA session was stronger than in the rest session (t_{20} = 2.12; p = 0.024, see ***Fig. 4D***).

Method:
First, we performed a one-way ANOVA to examine the differences in the number of ripples between the N-back conditions. Second, we asked whether inter-individual differences remain consistent across N-back conditions. That assesses if subjects with a higher number of ripples in one condition also have a higher number of ripples in another condition. To test this, we computed Pearson correlations of ripple counts across participants between different N-back blocks (2 vs. 3, 2 vs. 4, and 3 vs. 4), applying Bonferroni correction for multiple comparisons. Additionally, we correlated the ripple frequency in different N-back conditions with N-back (2-3-4, see ***Fig. 4C, D***), to estimate the linear association between ripple frequency and increasing task demands.

- The time scale in figure 5A is missing.
Reply: We thank the reviewer for point out this error, and changed the figure accordingly:

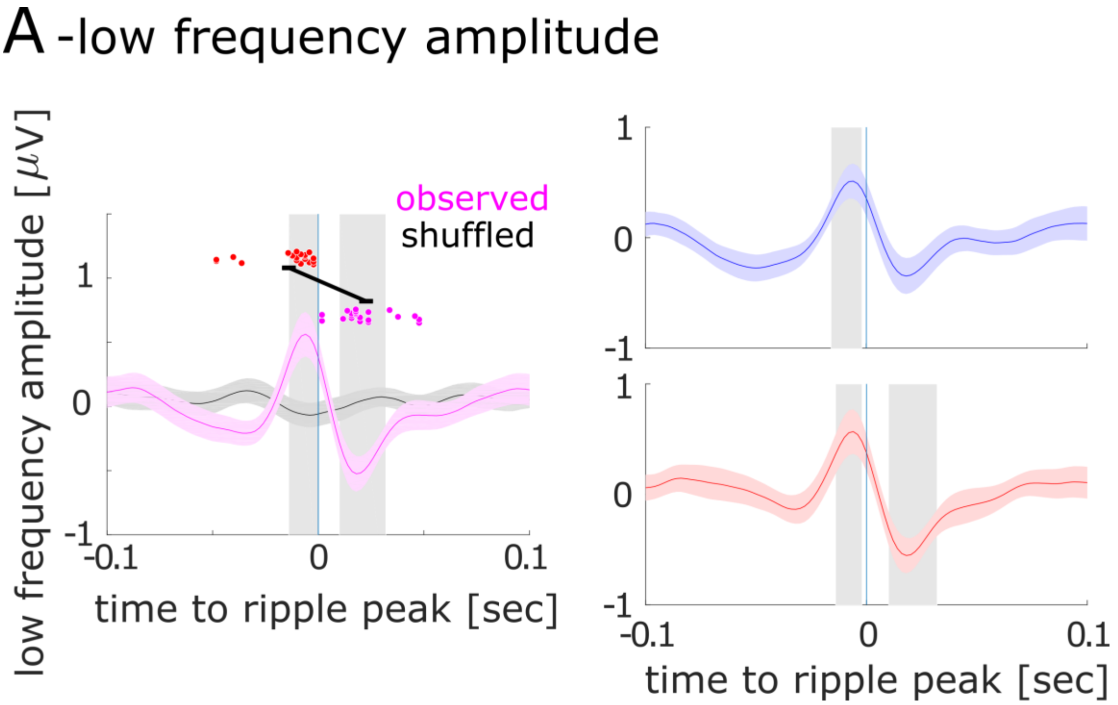

-Figure 6 was not cited in the text.

Reply: We thank the reviewer for highlighting this point and changed the text accordingly: We examined whether ripples were temporally locked to wake spindle events by comparing the frequency of ripples outside and within wake spindle events (see ***Fig. 6***, and Methods for a definition of wake spindle events). In the PA session, we observed more ripples within than outside wake spindle events (see ***Fig. 5C,*** $N_{in} = 0.45$; $N_{out} = 0.24$; $t_{20} = 3.25$; $p < .01$), but only a trend in the rest session ($N_{in} = 0.40$; $N_{out} = 0.27$; $t_{20} = 1.93$; $p = .068$).

References：

Andrillon, T., Nir, Y., Staba, R.J., Ferrarelli, F., Cirelli, C., Tononi, G., Fried, I., 2011. Sleep Spindles in Humans: Insights from Intracranial EEG and Unit Recordings. J. Neurosci. 31, 17821–17834. https://doi.org/10.1523/JNEUROSCI.2604-11.2011

Ayoub, A., Mölle, M., Preissl, H., Born, J., 2012. Grouping of MEG gamma oscillations by EEG sleep spindles. NeuroImage 59, 1491–1500. https://doi.org/10.1016/j.neuroimage.2011.08.023

Fernandez, L.M.J., Lüthi, A., 2020. Sleep Spindles: Mechanisms and Functions. Physiological Reviews 100, 805–868. https://doi.org/10.1152/physrev.00042.2018

Petzka, M., Chatburn, A., Charest, I., Balanos, G.M., Staresina, B.P., 2022. Sleep spindles track cortical learning patterns for memory consolidation. Current Biology 32, 2349-2356.e4. https://doi.org/10.1016/j.cub.2022.04.045

Schreiner, T., Petzka, M., Staudigl, T., Staresina, B.P., 2021. Endogenous memory reactivation during sleep in humans is clocked by slow oscillation-spindle complexes. Nat Commun 12, 3112. https://doi.org/10.1038/s41467-021-23520-2

Staresina, B.P., Niediek, J., Borger, V., Surges, R., Mormann, F., 2023. How coupled slow oscillations, spindles and ripples coordinate neuronal processing and communication during human sleep. Nat Neurosci 26, 1429–1437. https://doi.org/10.1038/s41593-023-01381-w

Zeitlhofer, J., Gruber, G., Anderer, P., Asenbaum, S., Schimicek, P., Saletu, B., 1997. Topographic distribution of sleep spindles in young healthy subjects. Journal of Sleep Research 6, 149–155. https://doi.org/10.1046/j.1365-2869.1997.00046.x
